# Meta-analysis of genome-wide DNA methylation identifies shared associations across neurodegenerative disorders

**DOI:** 10.1186/s13059-021-02275-5

**Published:** 2021-03-26

**Authors:** Marta F. Nabais, Simon M. Laws, Tian Lin, Costanza L. Vallerga, Nicola J. Armstrong, Ian P. Blair, John B. Kwok, Karen A. Mather, George D. Mellick, Perminder S. Sachdev, Leanne Wallace, Anjali K. Henders, Ramona A. J. Zwamborn, Paul J. Hop, Katie Lunnon, Ehsan Pishva, Janou A. Y. Roubroeks, Hilkka Soininen, Magda Tsolaki, Patrizia Mecocci, Simon Lovestone, Iwona Kłoszewska, Bruno Vellas, the Australian Imaging Biomarkers and Lifestyle study, the Alzheimer’s Disease Neuroimaging Initiative, Sarah Furlong, Fleur C. Garton, Robert D. Henderson, Susan Mathers, Pamela A. McCombe, Merrilee Needham, Shyuan T. Ngo, Garth Nicholson, Roger Pamphlett, Dominic B. Rowe, Frederik J. Steyn, Kelly L. Williams, Tim J. Anderson, Steven R. Bentley, John Dalrymple-Alford, Javed Fowder, Jacob Gratten, Glenda Halliday, Ian B. Hickie, Martin Kennedy, Simon J. G. Lewis, Grant W. Montgomery, John Pearson, Toni L. Pitcher, Peter Silburn, Futao Zhang, Peter M. Visscher, Jian Yang, Anna J. Stevenson, Robert F. Hillary, Riccardo E. Marioni, Sarah E. Harris, Ian J. Deary, Ashley R. Jones, Aleksey Shatunov, Alfredo Iacoangeli, Wouter van Rheenen, Leonard H. van den Berg, Pamela J. Shaw, Cristopher E. Shaw, Karen E. Morrison, Ammar Al-Chalabi, Jan H. Veldink, Eilis Hannon, Jonathan Mill, Naomi R. Wray, Allan F. McRae

**Affiliations:** 1 Institute for Molecular Bioscience, The University of Queensland, Brisbane, QLD 4072 Australia; 2 University of Exeter Medical School, RILD Building, RD&E Hospital Wonford, Barrack Road, Exeter, EX2 5DW UK; 3 School of Medical and Health Sciences, Edith Cowan University, 270 Joondalup Dr, Joondalup, WA 6027 Australia; 4 Department of Internal Medicine, Erasmus MC, University Medical Center, 3015GD Rotterdam, The Netherlands; 5 Murdoch University, 90 South St, Murdoch, WA 6150 Australia; 6 Australian Centre for Precision Health, University of South Australia Cancer Research Institute, School of Health Sciences, University of South Australia, Adelaide, SA 5001 Australia; 7 Brain and Mind Centre, Sydney Medical School, The University of Sydney, Sydney, Australia; 8 Centre for Healthy Brain Ageing, School of Psychiatry, University of New South Wales, Sydney, NSW 2031 Australia; 9 Neuroscience Research Australia Institute, Randwick, NSW 2031 Australia; 10 Griffith Institute for Drug Discovery (GRIDD), Griffith University, Brisbane, Australia; 11 Neuropsychiatric Institute, The Prince of Wales Hospital, UNSW, Randwick, NSW 2031 Australia; 12 Department of Neurology, University Medical Center Utrecht Brain Center, Utrecht University, Utrecht, The Netherlands; 13 Institute of Clinical Medicine, Neurology, University of Eastern Finland, Kuopio, Finland; 14 1st Department of Neurology, Memory and Dementia Unit, Aristotle University of Thessaloniki, Thessaloniki, Greece; 15 Department of Medicine, Institute of Gerontology and Geriatrics, University of Perugia, Perugia, Italy; 16 Department of Psychiatry, Warneford Hospital, University of Oxford, Oxford, UK; 17 Medical University of Lodz, Lodz, Poland; 18 INSERM U 558, University of Toulouse, Toulouse, France; 19 Centre for Motor Neuron Disease Research, Macquarie University, Sydney, NSW 2109 Australia; 20 Queensland Brain Institute, The University of Queensland, Brisbane, QLD 4072 Australia; 21 Centre for Clinical Research, The University of Queensland, Brisbane, QLD 4019 Australia; 22 Department of Neurology, Royal Brisbane and Women’s Hospital, Brisbane, QLD 4029 Australia; 23 Calvary Health Care Bethlehem, Parkdale, VIC 3195 Australia; 24 Fiona Stanley Hospital, Perth, WA 6150 Australia; 25 Notre Dame University, Fremantle, WA 6160 Australia; 26 Institute for Immunology and Infectious Diseases, Murdoch University, Perth, WA 6150 Australia; 27 The Australian Institute for Bioengineering and Nanotechnology, The University of Queensland, Brisbane, QLD 4072 Australia; 28 ANZAC Research Institute, Concord Repatriation General Hospital, Sydney, NSW 2139 Australia; 29 Discipline of Pathology and Department of Neuropathology, Brain and Mind Centre, The University of Sydney, Sydney, NSW 2050 Australia; 30 School of Biomedical Sciences, The University of Queensland, Brisbane, QLD 4072 Australia; 31 New Zealand Brain Research Institute, Christchurch, New Zealand; 32 Department of Medicine, University of Otago, Christchurch, New Zealand; 33 Eskitis Institute for Drug Discovery, Griffith University, Brisbane, Australia; 34 School of Psychology, Speech and Hearing, University of Canterbury, Christchurch, New Zealand; 35 Mater Research, Translational Research Institute, Brisbane, Australia; 36 Mater Research Institute, The University of Queensland, Brisbane, Australia; 37 Brain and Mind Research Centre, Sydney Medical School, The University of Sydney, Sydney, Australia; 38 Department of Pathology and Biomedical Science, University of Otago, Christchurch, New Zealand; 39 Department of Pathology, University of Otago, Christchurch, New Zealand; 40 School of Life Sciences, Westlake University, Hangzhou, Zhejiang China; 41 Westlake Laboratory of Life Sciences and Biomedicine, Hangzhou, Zhejiang China; 42 Centre for Genomic and Experimental Medicine, Institute of Genetics and Molecular Medicine, University of Edinburgh, Edinburgh, EH4 2XU UK; 43 Department of Psychology, Lothian Birth Cohorts group, University of Edinburgh, 7 George Square, Edinburgh, EH8 9JZ UK; 44 Department of Basic and Clinical Neuroscience, King’s College London, Institute of Psychiatry, Psychology and Neuroscience, London, SE5 9RX UK; 45 SITraN, University of Sheffield, Sheffield, UK; 46 Queen’s University Belfast, Belfast, Northern Ireland UK; 47 King’s College Hospital, London, SE5 9RS UK; 48 Institute of Psychiatry, Psychology & Neuroscience, King’s College London, London, SE5 8AF UK

**Keywords:** Neurodegenerative disorders, DNA methylation, Mixed-linear models, Methylation profile score, Out-of-sample classification, Inflammatory markers

## Abstract

**Background:**

People with neurodegenerative disorders show diverse clinical syndromes, genetic heterogeneity, and distinct brain pathological changes, but studies report overlap between these features. DNA methylation (DNAm) provides a way to explore this overlap and heterogeneity as it is determined by the combined effects of genetic variation and the environment. In this study, we aim to identify shared blood DNAm differences between controls and people with Alzheimer’s disease, amyotrophic lateral sclerosis, and Parkinson’s disease.

**Results:**

We use a mixed-linear model method (MOMENT) that accounts for the effect of (un)known confounders, to test for the association of each DNAm site with each disorder. While only three probes are found to be genome-wide significant in each MOMENT association analysis of amyotrophic lateral sclerosis and Parkinson’s disease (and none with Alzheimer’s disease), a fixed-effects meta-analysis of the three disorders results in 12 genome-wide significant differentially methylated positions. Predicted immune cell-type proportions are disrupted across all neurodegenerative disorders. Protein inflammatory markers are correlated with profile sum-scores derived from disease-associated immune cell-type proportions in a healthy aging cohort. In contrast, they are not correlated with MOMENT DNAm-derived profile sum-scores, calculated using effect sizes of the 12 differentially methylated positions as weights.

**Conclusions:**

We identify shared differentially methylated positions in whole blood between neurodegenerative disorders that point to shared pathogenic mechanisms. These shared differentially methylated positions may reflect causes or consequences of disease, but they are unlikely to reflect cell-type proportion differences.

**Supplementary Information:**

The online version contains supplementary material available at 10.1186/s13059-021-02275-5.

## Background

Neurodegenerative disorders are a heterogeneous group of disorders that cause progressive disruption of structure and function of the central or peripheral nervous system. Considerable genetic heterogeneity is often observed across patients [1, 2], who can display diverse clinical syndromes that mostly relate to specific brain regions affected by pathology [3]. Nonetheless, studies have also reported overlap between genetic risk factors, mechanisms, and pathological features of these disorders [3, 4]. Common neuronal pathways altered in multiple neurodegenerative disorders include protein quality control, the autophagy–lysosome pathway, mitochondria homeostasis, protein seeding and propagation of stress granules, synaptic toxicity, network dysfunction, and altered immune responses [3]. Importantly, the combination of unique and overlapping clinical and pathological features can lead to difficulty in the diagnosis of individual cases and perhaps calls for an overview of the biological processes that are shared or unique, to allow better classification of disease.

Genetic studies have been widely employed in relation to neurodegenerative disorders, with trait architecture mostly unique to each disease [5–7]. Although the heritability of neurodegenerative disorders ranges from 40 to 80% [8–11], a substantial fraction of the variance in liability is non-genetic, with robust evidence for environmental exposures as important contributors to disease pathogenesis [12]. DNA methylation which in mammals primarily refers to the reversible addition of a methyl group to a cytosine residue at a CpG dinucleotide is the most widely studied chemical modification of DNA. DNA methylation can repress transcription directly, by inhibiting the binding of specific transcription factors, or indirectly, by recruiting methyl-CpG-binding proteins and their repressive chromatin remodeling activities. There has been increasing evidence that alterations in DNA methylation play an important role in neurodegenerative and other brain disorders, with reports of significant associations with Alzheimer’s disease (AD) [13–16], Parkinson’s disease (PD) [17, 18], amyotrophic lateral sclerosis (ALS) [19, 20], and schizophrenia [21, 22], both in the brain and blood. Additionally, DNA methylation data can capture signatures of unmeasured environmental exposures. In this context, DNA methylation changes often show large effects; for example, composite DNA methylation scores explained 61% and 12.5% of the phenotypic variance of smoking status (current/ever/never) and alcohol intake (units per week), respectively [23]. Imputation of unmeasured environmental exposures could therefore help stratify patients across diagnostic boundaries, which may provide stimuli for additional analyses and clinical follow-up.

Using a similar concept to genome-wide association studies, methylome-wide association studies (MWAS) methods have started to emerge in order to address the effect of differentially methylated positions (DMPs) on complex diseases. Recently, the OmicS-data-based Complex trait Analysis (OSCA) software has been developed [24]. OSCA implements two reference-free mixed-linear model approaches that model different genome-wide architecture of DNA methylation: MOA and MOMENT (see the “Methods” section). Both methods have been shown, through extensive simulations [24], to better account for known (cell type proportion, smoking, age, batch effects) and unknown confounders than other methods. We have previously applied both MOA and MOMENT to two independently collected ALS case-control cohorts [20]. Both methods showed higher out-of-sample classification accuracy compared to linear regression, with MOMENT showing the best performance, despite detection of fewer significantly associated probes.

In this study, we investigated blood DNA methylation differences between patients and healthy controls across neurodegenerative disorders, including AD, ALS, and PD. We used both MOA and MOMENT to test for association between each DNA methylation site and the traits. We included schizophrenia, because of the previously reported genetic correlation between schizophrenia and ALS [25], and rheumatoid arthritis, a long-term autoimmune disorder, with a clearly defined pathogenic role of peripheral immune cells [26]. Analyses of schizophrenia and rheumatoid arthritis demonstrate if differences we find are specific to neurodegenerative diseases.

## Results

### Study design

**Fig. 1:**
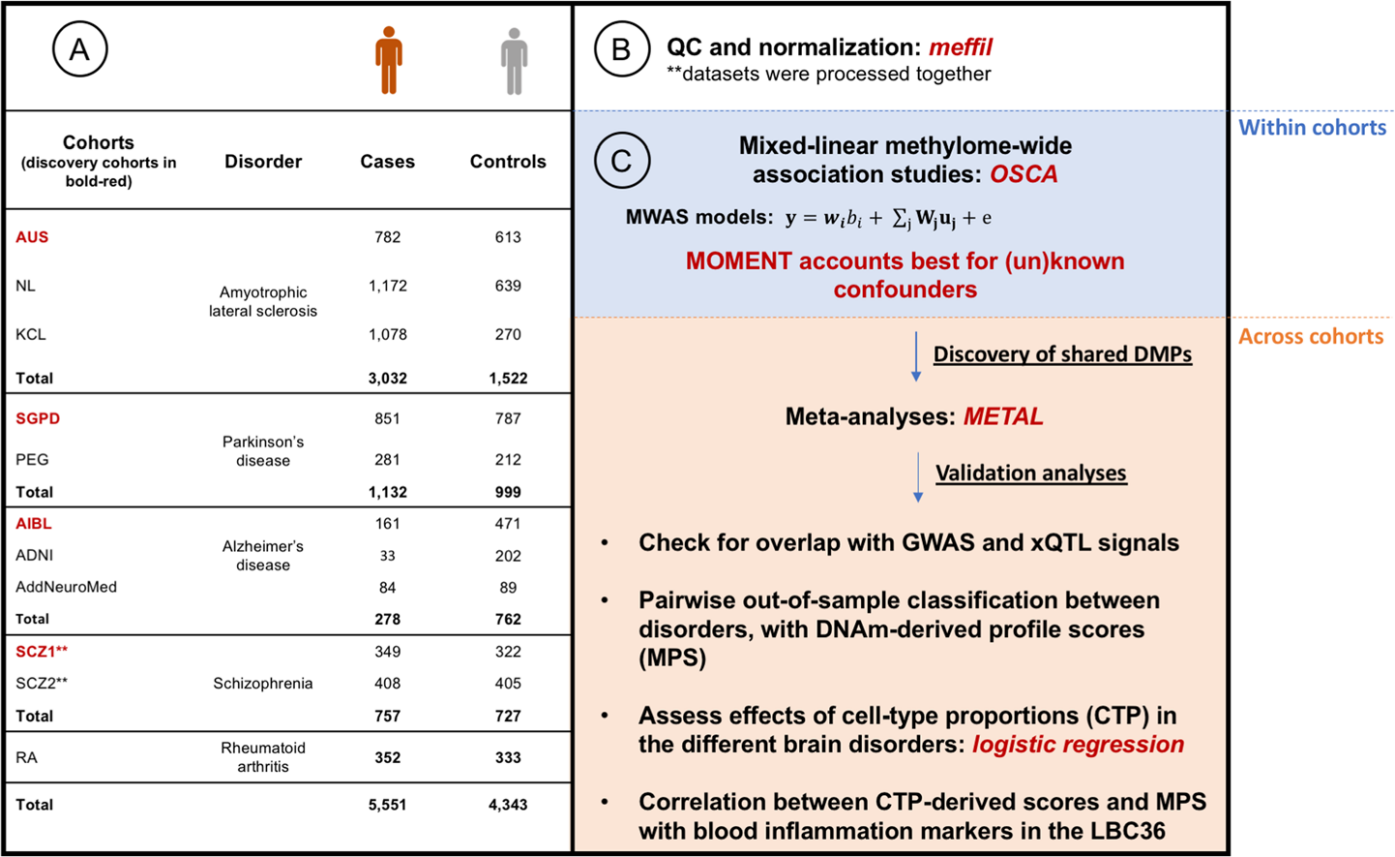
Study design flowchart. (1) Whole-blood DNA methylation (DNA methylation) data was available for three amyotrophic lateral sclerosis (AUS, KCL and NL), two Parkinson’s disease (SGPD and PEG), and three Alzheimer’s disease (AIBL, ADNI and AddNeuroMed), for which a subset of individuals was diagnosed with mild cognitive impairment (MCI). The MCI patients were not included in analyses, due to lack of power. We also had available two schizophrenia (SCZ1 and SCZ2) and one rheumatoid arthritis cohorts, used to check specificity of results to neurodegenerative disorders. In total, 5551 cases and 4343 controls were available for analyses, after quality control (QC). (2) QC and normalization of DNA methylation data were conducted using the R package *meffil* [27], which applies an automated estimation of functional normalization parameters that reduces technical variation in DNA methylation levels, thus reducing false positive rates and improving power. (3) To discover differentially methylated positions (DMPs), we applied mixed-linear model-based association studies of DNA methylation for each of the eight available cohorts, using two different methods: MOA and MOMENT [24]. To discover DMPs shared between neurodegenerative disorders, MOMENT results were meta-analyzed, between AUS, KCL, NL, SGPD, PEG, and AIBL cohort. We also found a similar distribution pattern of predicted immune cell-type proportions (CTP) between cases and controls of all disorders. We then attempted to validate our results using out-of-sample classification between disorders—with profile scores derived from CTP and DNA methylation effect sizes—and checking for overlap with GWAS, eQTL, mQTL, and haQTL (xQTLs) signals. Finally, we investigated the relationship between the CTP and DNA methylation-derived scores and blood inflammatory markers in a healthy aging cohort (Lothian Birth Cohort 1936)

Figure 1 shows an overview of the study design and analyses we used to investigate the shared DNA methylation alterations across brain disorders. After data preprocessing, quality control (QC), and normalization of DNA methylation data conducted in each cohort (see the “Methods” section and Additional file 1: Supplementary Note), 5551 genetically confirmed unrelated (except in the The Alzheimer’s Disease Neuroimaging Initiative (ADNI) and AddNeuroMed cohorts) cases and 4343 controls were available for analyses, across 11 different cohorts (Fig. 1).

DNA methylation was measured in whole blood, with 450k Illumina arrays, except for the Australian Imaging, Biomarkers and Lifestyle (AIBL) and ADNI cohorts, which were measured with EPIC Illumina arrays. Prior to analysis, we removed probes that failed QC, probes linked to sex-chromosomes, probes overlapping with SNPs, and probes with non-unique hybridization and extension, following recommendations described elsewhere [28]. We also excluded remaining probes on a per-cohort basis for which the standard deviation (s.d.) of measurements was < 0.02. This decision is justified, because power to detect an association depends, in part, on the variance between individuals and (standardized) effect sizes. Excluding these DNA methylation sites also reduces the multiple testing burden in association studies. The number of DNA methylation sites used for analyses in each cohort ranged from 206K to 254K, except for the AIBL cohort (EPIC array) for which 373 K sites passed QC (Additional file 2: Table S1). A description of the sample characteristics can be found in Additional file 1: Supplementary Note, Additional file 1: Figure S1 and Additional file 2: Table S2.

### Meta-analysis of MOMENT mixed-linear model association studies identifies differentially methylated positions across neurodegenerative disorders

To discover differentially methylated positions (DMPs) between cases and controls, we conducted MOMENT and MOA MWAS for each available cohort (Additional file 1: Figures S2, and S3, respectively). We have previously applied both MOA and MOMENT to two independently collected ALS case-control cohorts [20]. Both methods showed higher out-of-sample classification accuracy compared to linear regression, with MOMENT showing the best performance, despite detection of fewer significantly associated probes. Thus, our focus is the MOMENT results, but the MOA analyses aid with interpretation given potential confounding factors. We did not find evidence of genomic inflation with either method (i.e., the median of *χ*^2^ test-statistics of all DNA methylation sites divided by its expected value under the null: λ = [0.98–1.04]) (Additional file 1: Figures S2, and S3).

The only probe found to be significantly associated with schizophrenia in the MOMENT MWAS was cg05575921 (annotated to *AHRR*; *p* = 2.79 × 10^−27^) (Additional file 1: Figure S2), a well-replicated DMP that has been previously associated with cigarette smoking [23, 29–31]. Due to extensive epidemiological evidence that showed elevated smoking rates and intensity in patients with schizophrenia [32, 33], we fitted predicted smoking scores in the schizophrenia MWAS, to adjust for its confounding effect in all downstream analyses.

**Fig. 2:**
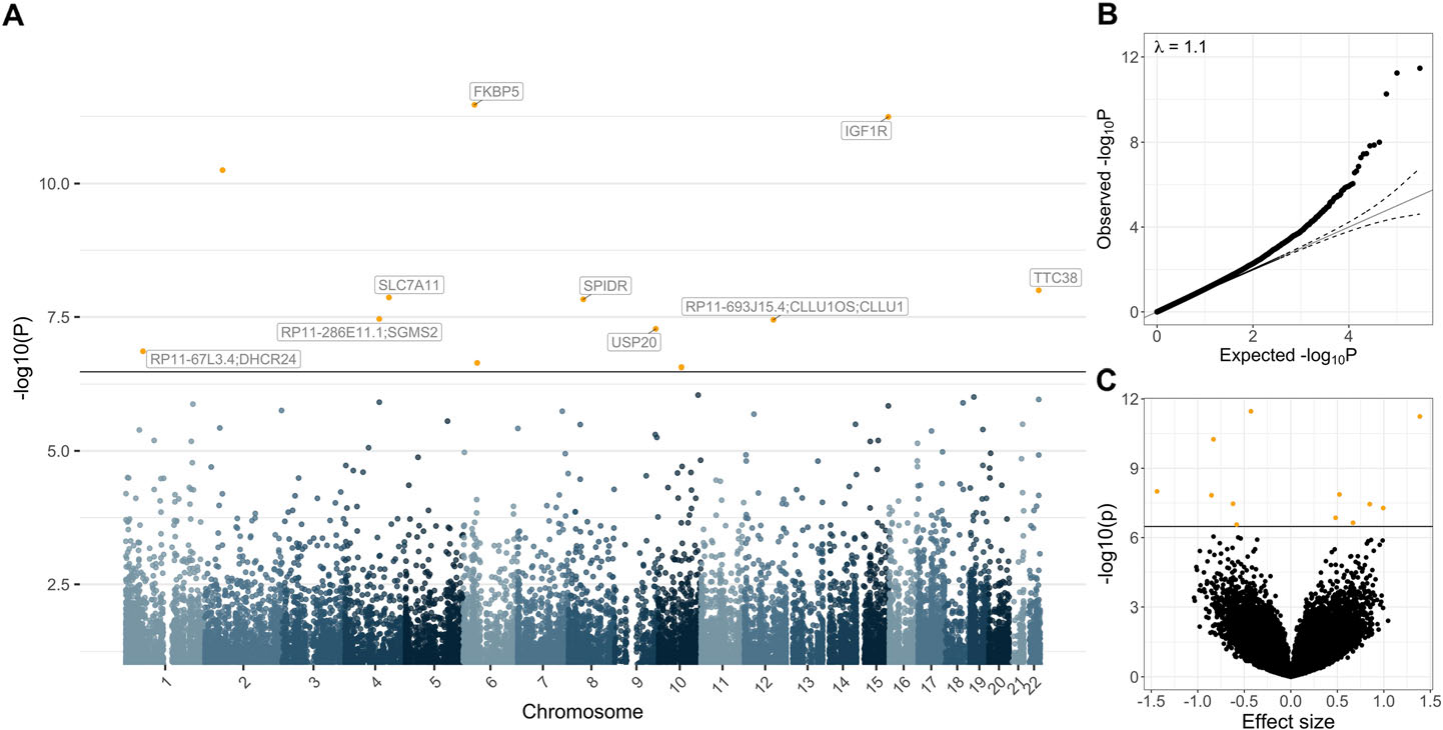
Manhattan (**a**), quantile-quantile (**b**) and volcano plots (**c**) of the MOMENT meta-analysis, of ALS, PD, and AD cohorts (*N*_cases_ = 4328, *N*_controls_ = 2994). The solid black lines in **a** and **c** refer to the genome-wide significant *p* value threshold (*p* = 3.30 × 10^−7^) and the dashed line refers to the suggestive *p* value threshold (*p* = 1 × 10^−5^). The dashed lines in **b** mark the upper and lower confidence intervals at 95%, for the *p* values. *λ* is the genomic inflation factor (the median of *χ*^2^ test-statistics of all DNA methylation sites divided by its expected value under the null)

Next, we applied fixed-effects inverse-variance-weighted [34] meta-analyses to the MOMENT results of the disease-specific cohort MWAS, i.e., within ALS (*N*_cases_ = 3032 and *N*_controls_ = 1522) (Additional file 1: Figure S4) and within Parkinson’s disease (PD) (*N*_cases_ = 1132 and *N*_controls_ = 999) (Additional file 1: Figure S5). We then meta-analyzed results across Alzheimer’s disease (AD), ALS and PD (*N*_cases_ = 4325, *N*_controls_ = 2992). The results for *m* = 151,506 probes (the low number of probes reflects different probes with s.d. < 0.02, between cohorts) included in the meta-analysis of the three neurodegenerative disorders can be found in Fig. 2a and show no evidence of genomic inflation (Fig. 2b, *λ* = 1.1). Compared to the very few genome-wide significant hits in the individual MWAS (Additional file 1: Figure S2), 12 CpGs pass the Bonferroni corrected genome-wide significance threshold (*p* = 3.30 × 10^−7^) (Table 1). In contrast, the MOA meta-analysis shows 41 genome-wide significant results (Additional file 1: Figure S6A), with higher genomic inflation (λ = 1.15) (Additional file 1: Figure S6B). Importantly, only five of the 12 DMPs were genome-wide significant in the ALS and PD MOMENT meta-analyses whereas the AD MOMENT MWAS (Additional file 1: Figure S2) showed no genome-wide significant associations (Additional file 2: Table S3). As expected, the significantly associated probes show the same direction of effect across all cohorts, but three probes show significant heterogeneity in effect size between cohorts [35] (*I*^*2*^ = 60.2%, probe cg06690548 in *SLC7A11*, *I*^*2*^ = 73.2%, for probe cg17901584 in RP11-67 L3.4;DHCR24 and *I*^*2*^ = 81.1% probe cg26033520) (Table 1).

**Table 1:** DNA methylation sites significantly associated with the traits at *p* < 3.3 × 10^−7^, in a MOMENT meta-analyses of AD, ALS and PD. Chr—chromosome number; Probe—probe identification number as provided by Illumina; bp—base pair position in the genome; Gene—closest genes the probe is annotated to, based on distance to transcription starting site, following the method described elsewhere [28]; Orien—DNA strand orientation, F = forward, R = Reverse [28]; *b*_META_—effects sizes (increase (positive sign) or decrease (negative sign) of methylation between cases and control per standard deviation unit) of meta-analysis results; *p*_META_—*p* values of meta-analysis models; s.e._META_—standard errors from meta-analysis; pMETA—*p* values from meta-analysis; Direction—direction of effect sizes, within each cohort (AUS, KCL, NL, SGPD, PEG, AIBL, respectively); *I*^2^—proportion of total variation in study estimates that is due to heterogeneity between the six cohorts in the meta-analysis [35]

Chr	Probe	bp	Gene	Orien	*b*_META_	s.e._META_.	*p*_META_	Direction	*I*^*2*^	*Q*	*p*_Q_
6	cg03546163	35686586	FKBP5	R	− 0.43	0.06	3.42 × 10^−12^	–	46.2	9.29	0.10
15	cg26272088	98900110	IGF1R	F	1.39	0.20	5.74 × 10^−12^	++++++	0	2.11	0.83
2	cg24166814	55840142	–	F	− 0.83	0.13	5.60 × 10^−11^	–	36.8	7.91	0.16
22	cg04431254	46288875	TTC38	F	− 1.44	0.25	1 × 0^−8^	–	10.4	5.58	0.35
4	cg06690548	138241654	SLC7A11	R	0.52	0.09	1.36 × 10^−8^	++++++	60.2	12.58	0.03
8	cg14195992	47353350	SPIDR	R	− 0.85	0.15	1.48 × 10^−8^	–	40.4	8.38	0.14
4	cg17786255	107893233	RP11-286E11.1; SGMS2	R	− 0.62	0.11	3.45 × 10^−8^	–	37.9	8.05	0.15
12	cg11881599	92420308	RP11-693 J15.4; CLLU1OS;CLLU1	R	0.85	0.15	3.57 × 10^−8^	++++++	0	2.25	0.81
9	cg13953978	129838509	USP20	F	0.99	0.18	5.28 × 10^−8^	++++++	0	0.93	0.97
1	cg17901584	54,888033	RP11-67 L3.4; DHCR24	F	0.48	0.09	1.39 × 10^−7^	+++++−	73.2	18.69	2.20 × 10^−3^
6	cg18120259	43926902	–	F	0.67	0.13	2.29 × 10^−7^	++++++	0	1.30	0.94
10	cg26033520	72244313	–	F	− 0.58	0.11	2.74 × 10^−7^	–	81.1	26.40	7.47 × 10^−5^

Adding the schizophrenia cohorts to the analysis results in twelve additional genome-wide significant CpGs (Additional file 1: Figure S7A), with effect sizes highly correlated between analyses (*r*_Pearson_ = 0.89, *p* < 2.2 × 10^−16^, Additional file 1: Figure S7B). Lastly, we added the rheumatoid arthritis cohort to the meta-analysis with AD, ALS, and PD, as a positive control, i.e., to demonstrate the specificity of results to neurodegenerative or brain disorders. Interestingly, we found two additional genome-wide significant probes: cg01447828, annotated to gene *PRX* (p_META_NDs_RA_ = 1.48 × 10^−7^) and cg03785076, annotated to gene *SNED1* (p_META_NDs_RA_ = 3.1 × 10^−7^) (Additional file 1: Figure S7C), with effect sizes highly correlated between analyses (*r*_Pearson_ = 0.95, *p* < 2.2 × 10^−16^, Additional file 1: Figure S7D).

### GWAS signals do not overlap with loci centered at the 12 differentially methylated positions

We next investigated if the 12 DMPs overlapped with brain [36] or blood [37] methylation quantitative trait loci (mQTL) regions (*p* < 5 × 10^−8^) and SNPs (*p* < 5 × 10^−8^) from a sample-size weighted meta-analysis of publicly available GWAS summary statistics for AD (*N* = 368,440) [38], ALS (*N* = 80,610) [39] and PD (minimum *N* = 520, maximum *N* = 482,730, excluding the 23andMe cohort) [40] (Additional file 1: Figure S8). Three of the 12 DMPs overlapped with blood mQTLs (number of mQTLs; *m* = 266), and another four overlapped with brain mQTLs (*m* = 86) with the top mQTLs presented in Additional file 2: Table S4. We found no evidence for mQTL overlap with GWAS hits (Additional file 2: Table S4), which could reflect lack of power in both MWAS and GWAS, as has previously been observed for body-mass index (BMI) [41].

Since lack of power could hide potential causal genetic relationships to disease with marginal signals in GWAS that may be present in the DNA methylation data as well, we further investigated whether the loci located ± 500 kb of each of the 12 DMPs overlapped with the previously mentioned GWAS signals from each disorder and our meta-analysis (Additional file 1: Figures S9-S12). For AD, we additionally compared with GWAS results from clinically diagnosed AD cases, from Kunkle et al. (*N* = 63,926) [42] (Additional file 1: Figure S13). As for the mQTL analysis, we found no evidence for overlap with GWAS signals (i.e., with *p* < 5 × 10^−8^) in the pre-defined loci. The strongest overlapping signal was from our meta-analysis of AD, ALS, and PD GWAS, albeit non-significant: SNP rs112184630 (*p*_META_GWAS_ = 5.91 × 10^−7^), located in chromosome 9q, which has been shown to be a blood eQTL for genes TOR1A (*p* = 6.74 × 10^−16^), FBP1 (*p* = 3.04 × 10^−15^), C9orf78 (*p* = 9.52 × 10^−12^) [43], and also PTGES (*p* = 7.92 × 10^−6^) [44].

Finally, we expanded our query within these loci to brain eQTL (*N* = 1433), mQTL (*N* = 411), and haQTL (*N* = 411) summary statistics from the AMP-AD consortium [45, 46]. Six of the twelve loci showed significant signals across all xQTLs, suggesting these results could be relevant to brain tissue in a panQTL manner (Additional file 1: Figure S14). The top eQTL per gene in the queried 1 Mb window can be found in Additional file 2: Table S5.

### Out-of-sample classification accuracy within- and across-disorders from DNA methylation-derived profile scores

Out-of-sample classification provides independent evidence that differences in DNA methylation between cases and controls reflect differences associated with disease status rather than technical confounding effects, although they could also reflect shared disease-associated confounders (e.g., smoking status and schizophrenia). It can also leverage DNA methylation differences between cases and controls that do not achieve statistical significance. Thus, we performed pairwise out-of-sample classification using DNA methylation-derived profile scores (MPS), with DNA methylation effect sizes as weights multiplied by each corresponding site in the target cohort. MPS were calculated keeping effect sizes that passed different *p* value thresholds in each MOA or MOMENT MWAS. Classification accuracy of the MPS was evaluated by the area under the receiver-operator characteristic (ROC) curves (AUC) (see the “Methods” section). AUC ranges from 0.5 (random classification) to 1 (perfect classification) and can be interpreted as the probability that a case ranks higher than a control (either in the sample or in the population from which the sample was drawn). We use the notation AUC_X:Y_ to denote a predictor based on probes and effect sizes estimated in data set of disorder X and used to classify cases and controls of disorder Y.

**Fig. 3:**
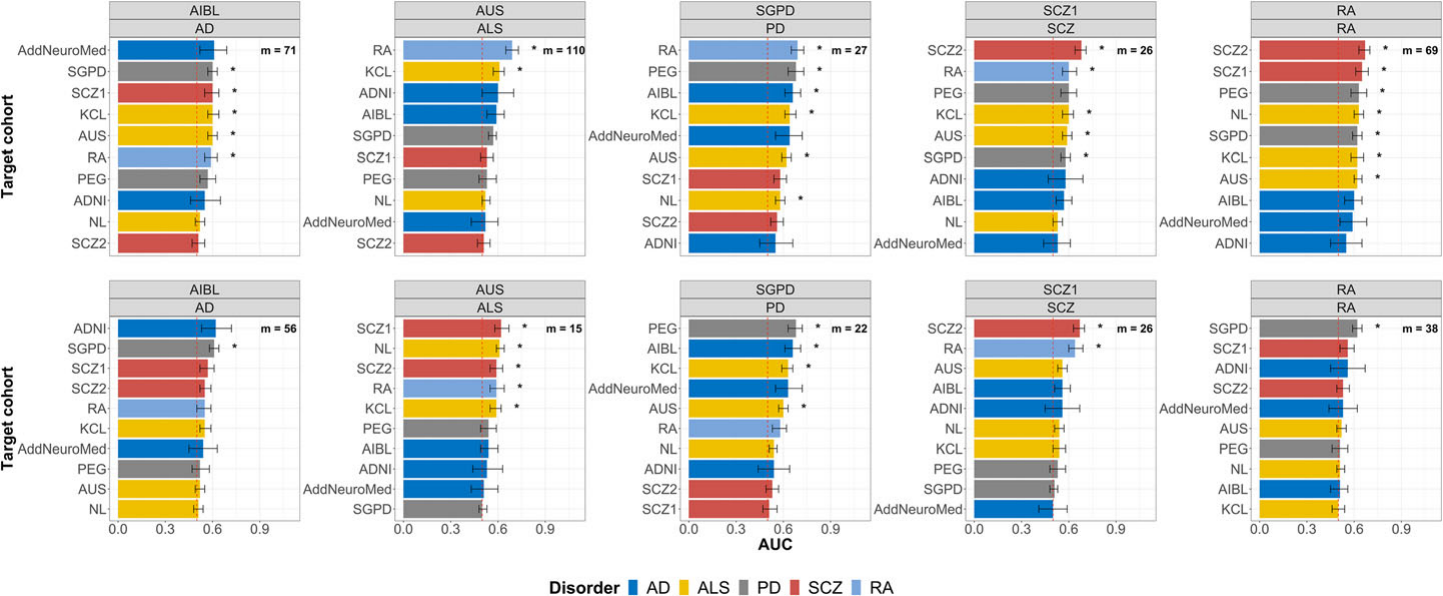
Accuracy of out-of-sample classification in each target cohort, measured by the area under the curve (AUC) statistics obtained from DNA methylation profile scores (MPS), using MOA (top-row) or MOMENT (bottom-row) results at *p* value < 1 × 10^−4^, from each discovery cohort (column). AD, Alzheimer’s disease (dark blue); ALS, amyotrophic lateral sclerosis (yellow); PD, Parkinson’s disease (gray); RA, rheumatoid arthritis (light blue); SCZ, schizophrenia (red). Bars indicate 95% confidence intervals of AUC values; *m* = number of probes used in the classifier; stars represent *p* values lower than Bonferroni threshold (i.e., *p* < 0.05/700 tests), from logistic regression

The maximum AUC obtained with MOA- and MOMENT-MPS is summarized in Additional file 1: Figure S15. The maximum AUC is obtained when classifying rheumatoid arthritis cases from controls with any other brain disorder used as the discovery MWAS, using MOA-MPS calculated from DNA methylation sites associated at less stringent *p* value thresholds. These results may sound surprising, since one would expect brain disorders to share more similarly disrupted DNA methylation patterns with each other than with rheumatoid arthritis. However, in the majority of cases, and particularly obvious at lower *p* value thresholds (Fig. 3), these AUC patterns differed for MOA- and MOMENT-MPS, with MOMENT-MPS giving higher AUC values within-disorders. For instance, when we use the System Genomics of Parkinson’s disease (SGPD) cohort DNA methylation effect sizes with *p* < 1 × 10^−4^ to calculate the MPS, the maximum MOA-MPS AUC is obtained when classifying rheumatoid arthritis patients from controls (AUC_SGPD:RA_ = 0.69, m = 27, *p* = 9.1 × 10^−16^), whereas the maximum MOMENT-MPS AUC is obtained when classifying PD patients from controls (AUC_SGPD:PEG_ = 0.68, *m* = 26, *p* = 1 × 10^−9^) and AUC_SGPD:RA_ is much reduced with MOMENT-MPS (AUC_SGPD:RA_ = 0.58, *m* = 27, *p* = 6.8 × 10^−4^) (Fig. 3). Since rheumatoid arthritis is a long-term autoimmune disorder, with a clearly defined pathogenic role of peripheral immune cells [26], we hypothesized that the effects of cell-type composition, particularly at higher *p* value thresholds, were driving the high AUC values.

### Analysis of DNA methylation-derived immune cell-type proportions

**Fig. 4:**
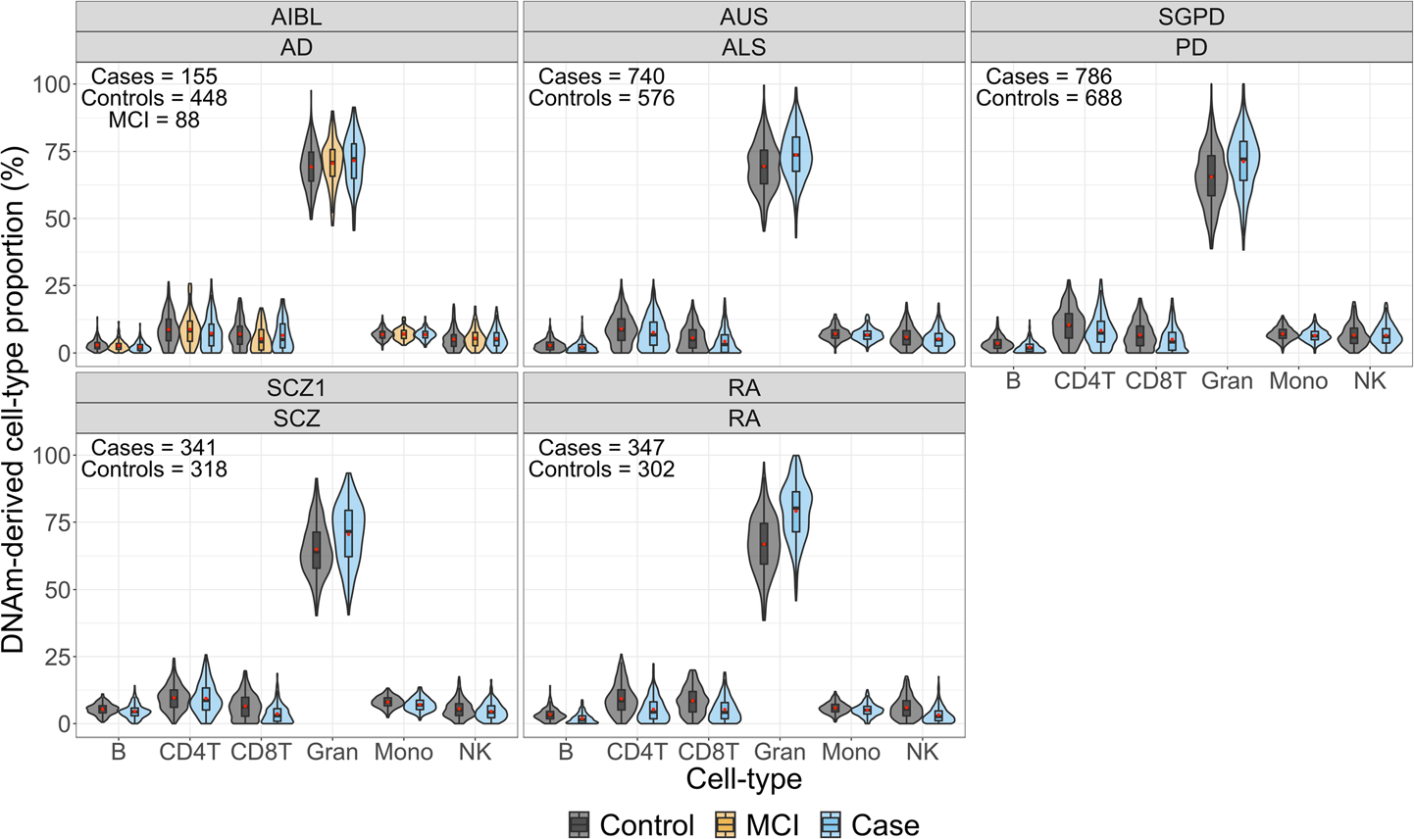
Violin plots of predicted cell-type proportions (CTP) in cases and controls of each discovery cohort. ALS, amyotrophic lateral sclerosis; AD, Alzheimer’s disease; MCI, mild cognitive impairment; PD, Parkinson’s disease; RA, rheumatoid arthritis; SCZ, schizophrenia. The boxplot horizontal black line marks the median CTP value in that group. The red circle inside the boxplots marks the mean CTP value in that group. The lower and upper hinges correspond to the first and third quartiles (the 25th and 75th percentiles). The upper whisker extends from the hinge to the largest value no further than 1.5 * IQR from the hinge (where IQR is the inter-quartile range, or distance between the first and third quartiles). The lower whisker extends from the hinge to the smallest value at most 1.5 * IQR of the hinge

We used the EpiDISH algorithm [47] to predict immune cell-type proportions (CTP) of B lymphocytes, CD4+ T lymphocytes, CD8+ T lymphocytes, granulocytes, monocytes, and natural killer cells, as empirical cell-type measurements were not available to us. Visual inspection of the CTP distributions between cases and controls showed very similar patterns between all disorder (Fig. 4). A subset of individuals in the AD cohorts was diagnosed with mild-cognitive impairment (MCI). Although we did not include these individuals in any analyses due to lack of power, the upwards trend in granulocytes (and in turn a decrease in other CTP) is worthy of note, with MCI patients showing a midway value between controls and AD patients (Fig. 4). These results were replicated across all AD cohorts (Additional file 1: Figure S16), suggesting a potential link with disease progression (that can be cause or consequence of disease).

To assess the association between each CTP and disorder we used multiple logistic regression models, with case-control status of the discovery cohorts fitted as response variable and CTP (excluding CD8+ T cells, due to redundancy in proportion data and their lower abundancy), sex, DNA methylation age [48] and DNA methylation-derived smoking score [23] fitted as covariates. We excluded individuals with outlying CTP values (*N* = 464) prior to fitting the models (see the “Methods” section). We found no significant associations of estimated proportion of cell-types for the AIBL and the Australian ALS cohort (AUS), after Bonferroni correction (i.e., *p* < 0.01) (Additional file 2: Table S6). The odds ratio (OR) of being classified as a case given an increase in granulocytes proportions (per one-point percentage increase) ranged from 1.02–1.12, after controlling for the other covariates. The highest OR were observed for the rheumatoid arthritis (OR = 1.12, CI_95%_ = [1.07–1.18], *p* = 4.4 × 10^−7^) and the SCZ1 cohort (OR = 1.12, CI_95%_ = [1.04–1.19], *p* = 1.67 × 10^−3^) and the SGPD cohort (OR = 1.04, CI_95%_ = [1.01–1.07], *p* = 2.82 × 10^−3^). Notably, we found the direction of effect sizes to be highly consistent between disorders for some of the CTPs (e.g., granulocytes), but in different directions for other cell-types (e.g., B lymphocytes) (Additional file 2: Table S6).

We also assessed the pairwise out-of-sample classification accuracy of CTP-derived scores (sum scores of effect sizes estimated from the previously described logistic regression models, multiplied by the corresponding CTP in the target cohort) (Additional file 1: Figure S17). Similar to the MPS, the maximum AUC using CTP-scores is obtained when classifying rheumatoid arthritis cases from controls, strengthening our hypothesis that these results are mainly driven by CTP. Indeed, if we adjust the MPS that give maximum AUC by the CTP in a linear model and use the residuals to determine the AUC of the resulting ROC curves, the overall maximum accuracy of both MOA and MOMENT MPS goes down (Additional file 1: Figure S18).

### Significant correlation of CTP profile scores with blood protein markers of inflammation in a healthy aging cohort

To determine if CTP-scores and MPS were capturing immune-related signals, we calculated correlations between these scores and blood inflammatory markers in the Lothian Birth Cohort 1936 (LBC1936). The LBC1936 is a healthy aging elderly cohort for whom we had access to whole blood DNA methylation data measured from 450k Illumina arrays (*N* = 980), 92 blood inflammatory protein markers measured with the Olink® inflammation panel (pg/mL) (*N* = 1048), and real cell-counts (10^9^/L), including lymphocytes, monocytes, and granulocytes measured at wave 1 (*N* = 909). After quality control (see the “Methods” section), 823 individuals had blood DNA methylation, measured cell counts and inflammatory markers measures available, and were included in the correlation analyses.

**Fig. 5:**
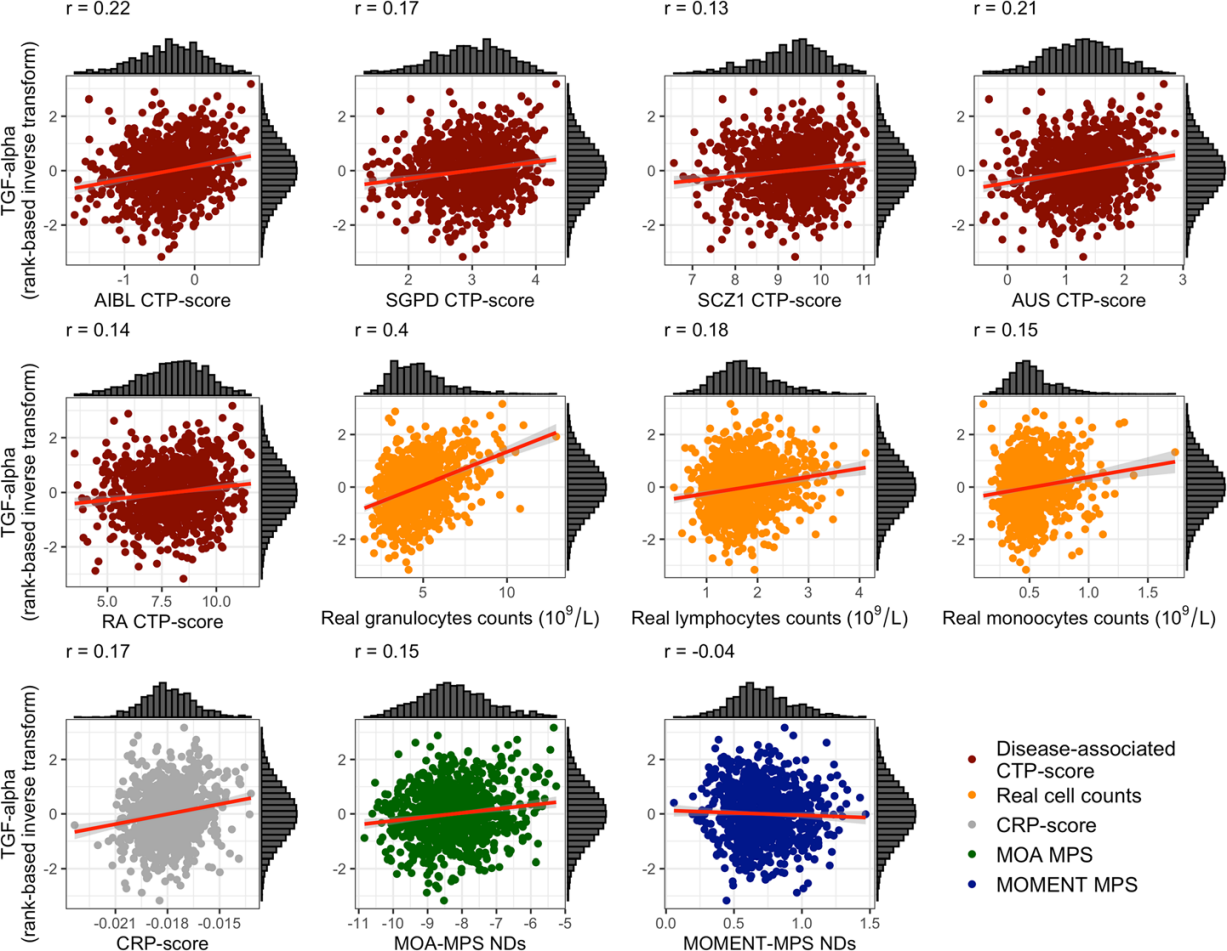
Scatterplot of TGF-alpha and disease-associated CTP-scores, real white blood cell counts, CRP-MPS, MOA-MPS, and MOMENT-MPS in the Lothian Birth Cohort 1936 (*N* = 823). Scatterplots and marginal histograms of TGF-alpha (rank-based inverse transform) vs disease-associated CTP-scores (dark red), real white blood cell counts (10^9^/L, in orange), DNA methylation-derived CRP-scores (gray), MOA- (dark green), and MOMENT-MPS of meta-analyses of three neurodegenerative disorders (dark blue), which included amyotrophic lateral sclerosis, Alzheimer’s disease, and Parkinson’s disease. The red line shows the best linear fit to the data, with gray background representing the s.e.

As expected, many blood inflammation markers are highly correlated with measured white cell counts (Additional file 1: Figure S19), although the observed absolute magnitude of correlation coefficients was low to moderate (|*r*_Pearson_| = 0 to 0.4). Some markers, such as transforming-growth factor alpha (TGF-alpha), showed moderate positive correlations with all disease-associated CTP-scores: AIBL CTP-scores (*r*_Pearson_ = 0.22, *p* = 2.78 × 10^−10^), AUS CTP-scores (*r*_Pearson_ = 0.21, *p* = 1.81 × 10^−9^), SGPD CTP-scores (*r*_Pearson_ = 0.17, *p* = 9.88 × 10^−7^), rheumatoid arthritis CTP-scores (*r*_Pearson_ = 0.14, *p* = 9.41 × 10^−5^), and schizophrenia CTP-scores (*r*_Pearson_ = 0.13, *p* = 2.21 × 10^−4^) (Fig. 5). To the contrary, markers such as TNF-beta were negatively correlated with CTP-scores from neurodegenerative disorders specifically: SGPD CTP-scores (*r*_Pearson_ = − 0.23, *p* = 2.38 × 10^−11^), AIBL CTP-scores (*r*_Pearson_ = − 0.18, *p* = 2.08 × 10^−7^), and AUS CTP-scores (*r*_Pearson_ = − 0.11, *p* = 3.52 × 10^−3^) (Additional file 1: Figure S20). All disease-associated CTP-scores were highly positively correlated with granulocyte counts (*r*_Pearson_ = [0.49–0.62]) and negatively correlated with lymphocyte counts (*r*_Pearson_ = [− 0.32 to − 0.43]) (Additional file 1: Figure S21), with the difference in sign of correlation likely a consequence that the CTP-scores were estimated from proportion data.

Additionally, we investigated the relationship between the protein inflammatory markers, with three DNA methylation-derived scores: a C-reactive protein score (CRP-MPS), derived from a chronic low-grade inflammation MWAS [49, 50] and MOA- and MOMENT-MPS, calculated using genome-wide significant probes that were present in the LBC1936, from the MOMENT (Fig. 2) or MOA (Additional file 1: Figure S3) meta-analyses of neurodegenerative disorders, as weights (*m* = 11 and *m* = 38, respectively). It is important to note that the LBC1936 was used to estimate the effect sizes used as weights in the CRP-MPS and thus these may be slightly inflated. We note both CRP-MPS and MOA-MPS are highly positively correlated with many markers of inflammation, in contrast to MOMENT-MPS (Additional file 1: Figure S22). This is also reflected in the correlation between scores: MOA-MPS are strongly positively correlated with all CTP-scores, CRP-MPS, and granulocytes counts (*r*_Pearson_ = [0.50–0.77]), whereas MOMENT-MPS are strongly negatively correlated (*r*_Pearson_ = [− 0.29 to − 0.59], suggesting MOMENT could be over-correcting for CTPs (Additional file 1: Figure S21).

## Discussion

In this study, we aimed to identify shared effects of blood DNA methylation in three neurodegenerative disorders: ALS, AD, and PD. Here, we used MOA and MOMENT [24], two mixed-linear model methods, to test for association of each DNA methylation site with the traits on a per-cohort basis (Fig. 1). In simulations, MOMENT has been shown to be more powerful and generate fewer false positive associations than standard methods, given known and unknown confounding effects that can generate high correlation of DNA methylation levels across the genome. Our focus is thus the MOMENT results, but the MOA analyses aid with interpretation given potential confounding factors. While only three probes were found to be genome-wide significant in each MOMENT association analyses of ALS and PD (and none with AD), in the MOMENT meta-analysis of the three disorders, 12 DMPs passed the Bonferroni-corrected genome-wide significance threshold (*p* = 3.30 × 10^−7^, Table 1). This result is not expected by chance and implies that neurological disorders may have similarly affected biological mechanisms in blood, although whether this is part of the causal pathway to disease or as a result of disease cannot be shown from these data. We did not find any overlap with the top signals from a previous AD blood MWAS [15]. Five and 6 DMPs from our meta-analysis overlapped with the top signals from a blood MWAS of PD [18], and ALS [20], respectively (Additional file 1: Figure S23), but it is important to note the PD and ALS cohorts from these studies were included in our meta-analysis.

The 12 DMPs were annotated to genes which encoded proteins involved in intracellular trafficking/protein quality control, such as co-chaperone activities involved in glucocorticoid signaling, deubiquitination involved in beta-2 adrenergic receptor recycling and anionic amino-acid transport (*FKBP5*, *USP20*, and *SLC7A11*, respectively), tyrosine kinase activity involved in cell growth and survival control (*IGF-1R*), double-stranded DNA damage repair (*SPIDR*), and lipid metabolism (*SGMS2* and *DHCR24*). Lastly, some of these probes were also annotated to intergenic regions and to a gene with unknown function (*CLLU1*) that has mainly been studied in the context of chronic lymphocytic leukemia [51].

The probe cg03546163, located at the promoter of gene *FKBP5*, showed the strongest association in the MOMENT meta-analysis of neurodegenerative disorders (*p*_META_ = 3.42 × 10^−12^, decreased blood DNA methylation in cases compared to controls). Demethylation at this site has previously been reported in patients with Cushing’s syndrome (marked by chronic excess and attenuation of the endogenous diurnal variation in cortisol secretion) [52] and Behçet’s disease compared to controls, in the same direction of effect as for neurodegenerative disorders. Especially relevant to neurodegenerative disorders is the finding that *FKBP5* expression has been shown to progressively increase with normal aging, concomitant with reduced *FKBP5* DNA methylation [53], which correlated with Braak staging in human brains and increased tau pathology both in vitro and in mouse models of AD [53, 54]. Additionally, a recent study suggested a model whereby aging and stress-related phenotypes synergize to decrease DNA methylation at selected enhancer-related FKBP51 sites, epigenetically upregulating FKBP5 in whole blood and in distinct immune cell subtypes. Higher FKBP5 in turn would promote NF-κB (nuclear factor kappa-light-chain-enhancer of activated B cells)-driven peripheral inflammation [55]. Nonetheless, it is important to note that in all these studies, the authors did not adjust for cell type composition, a widely recognized confounder in DNA methylation studies [56, 57]. Although we find reassurance in these past findings, we also point out a negative correlation between predicted granulocyte proportions and DNA methylation levels (and positive correlations with other cell subtypes due to the nature of proportion data) at cg03546163, which does not exclude confounder effects induced by shifts in CTP between cases and controls (Additional file 1: Figure S24). Due to space restrictions we provide a more thorough discussion of the remaining associated probes in Additional file 1: Supplementary Note.

The addition of schizophrenia and rheumatoid arthritis cohorts to the meta-analysis of neurodegenerative disorders resulted in additional genome-wide significant associations, a result that is also not expected by chance and implies that neurological disorders may have similarly affected biological mechanisms in blood with psychiatric and auto-immune disorders. For example, the association with probe cg01447828 (*p*_META_NDs_ SCZ_ = 1.17 × 10^−7^, *p*_META_NDs_RA_ = 1.48 × 10^−7^, decreased methylation blood DNA methylation in cases compared to controls), annotated to the gene *PRX* encoding periaxin, is significant only when adding schizophrenia or rheumatoid arthritis to the MOMENT meta-analyses. Periaxin is a structural membrane-associated protein required for maintenance of the myelin sheath of peripheral Schwann cells and normal remyelination after nerve injury. We note that mutations in *PRX* have been previously shown to cause peripheral neuropathies [58, 59], which are not uncommon in Parkinson’s disease and rheumatoid arthritis patients, but do not speculate on the relationship between these observations.

Some case-control DNA methylation differences, driven by aging, cell type composition, or environmental exposures, medications, or complications of the disease, could be considered confounders to the trait of primary interest, or alternatively, could be of primary interest themselves depending on the context of the scientific question. While the effects of aging in neurodegenerative disorders are well-recognized and widely studied [60], associations with other risk factors such as smoking or heavy metals are still highly debated. In the context of cell-type composition, there is accumulating evidence for an active role of immune cells, and inflammation in general, in neurodegenerative disorders (both as cause and consequence of disease) [61, 62]. We have found CTP to be major drivers of high MPS classification accuracy (as measured by AUC), given by MPS calculated at lower *p* value thresholds. Although at more stringent *p* value thresholds, AUC values given by MOMENT-MPS were generally higher when classifying within-disorders, in contrast to MOA-MPS, we also note the overlap of confidence intervals between AUC, and thus disease-specificity cannot be completely assumed (Fig. 3).

To help us determine if the associations with the 12 DMPs in the MOMENT meta-analysis were due to confounding with CTP, we assessed the relationship between MPS derived from the 12 DMPs and well-known blood inflammatory markers, in a healthy aging cohort. We then compared the results obtained when utilizing disease-associated scores derived from CTP, low-grade chronic inflammation-related CRP-scores, or with measured cell counts. As expected, we found measured cell counts, CTP-scores, and low-grade chronic inflammation-related CRP-scores to be highly positively correlated with blood protein inflammatory markers, whereas MOMENT-MPS (derived from genome-wide significant results of a meta-analysis of ALS, AD and PD) showed no statistically significant correlations (Additional file 1: Figure S19), suggesting these MPS are not strongly confounded by CTP.

Our study has several limitations. Firstly, although growing in number, the current scarcity of homogeneously collected DNA methylation datasets, largely from small cohorts, makes it difficult to perform replication analyses, particularly in a mixed-linear model setting due to lack of power, as we previously performed for ALS and PD [18, 20]. Secondly, a perceived limitation of our study is that blood is not a relevant tissue for understanding the biological mechanisms underlying brain disorders, due to the tissue-specificity of most DNA methylation patterns. However, in the context of diagnosis/prognosis or biomarker discovery, more accessible peripheral tissues (e.g., whole blood), will always prove more useful, especially if the concordance of genome-wide DNA methylation of the tissue analyzed with DNA methylation in live brain tissue is taken into consideration [36, 63, 64]. This hypothesis is further supported by findings that variation between people in DNA methylation controlled by SNP variation has been shown to have high correlation between brain and blood [36, 64]. Lastly, as real cell-type composition measures were unavailable we studied predicted cell-type proportions from DNA methylation. These CTP are inherently estimated with error (Additional file 1: Figure S25), which also depends on DNA methylation measurement noise that may be cohort-dependent. Furthermore, CTP-scores are highly correlated with granulocyte proportions (Additional file 1: Figure S21), because these are the most abundant cell types in whole blood potentially masking biologically relevant actions from other cell-types.

Although it is economically unfeasible to collect DNA methylation data at single-cell resolution for these sample sizes, these results advocate that it would be good practice to collect single-cell data in at least a subset of individuals to validate results from whole-blood analysis. A similar conclusion is supported by a recent study assessing the co-variability of DNA methylation across peripheral cells and tissue [65]. We annotated our 12 DMPs from the MOMENT meta-analysis to characteristic scores supplied by the authors, to determine which cell types are potentially affected by the significant differences reported (Additional file 2: Table S7). The scores for each DNA methylation site and cell type were calculated by fitting a one-sided Levene’s test comparing the variation of a single cell type against the variation across all samples from the other four cell types, specifically testing for a larger variance in that cell type (i.e., one-tailed test). DNA methylation sites were determined to be characteristic of single cell type if the *P* value from Levene’s test was < 9 × 10^−8^. We have found only cg04431254 and cg11881599 reflected variation in a single cell type, CD8T and B cells, respectively. None of the remaining probes reflected significant variation in any of the cell types, although it is possible that the cell-type driving variation at this site have not been interrogated in this study.

Despite some limitations, taken together these results show the presence of aberrant peripheral DNA methylation differences and CTP distribution patterns that point to shared pathogenic mechanisms between neurodegenerative disorders, which are likely a reflection of neuroinflammatory dysregulation. Larger samples with blood collected prior to diagnosis and with deep clinical phenotyping are needed to allow investigation of the potential predictive ability of DNA methylation/CTP-based biomarkers in neurodegenerative disorders and to distinguish which of these mechanisms are cause or consequence of disease.

## Conclusions

In this study, we identified shared DMPs, in whole blood, and similar CTP distribution patterns between neurodegenerative disorders that point to shared pathogenic mechanisms, which are likely a reflection of neuroinflammatory dysregulation. Larger samples with blood collected prior to diagnosis and with deep clinical phenotyping are needed to allow investigation of the potential predictive ability of DNA methylation/CTP-based biomarkers in neurodegenerative disorders and to distinguish which of these mechanisms are cause or consequence of disease.

## Methods

### Cohorts description

#### Amyotrophic lateral sclerosis (ALS) cohorts

##### Australian ALS cohort

The Australian ALS cohort (AUS) and DNA methylation assays have been previously described elsewhere [20]. Part of the Australian sample comprised patients and controls that were ascertained from the University of Sydney as part of the Australian MND DNA bank, which recruited participants from April 2000 to June 2011. Cases were white Australians older than 25 years recruited from around Australia via state-based MND associations with diagnosis verified by a neurologist. Control individuals were either partners or friends of patients with ALS or community volunteers. The remainder of Australian cases were recruited from clinics across Australia between 2015 and 2017 diagnosed with definite or probable ALS according to the revised El Escorial criteria [66]. Control subjects were healthy individuals free of neuromuscular diseases, recruited as either partners or friends of patients with ALS or community volunteers or from the Older Australian Twin Study (OATS) [67]. ALS cases with a recorded family history of ALS were excluded. The DNA methylation was measured using Illumina Infinium HumanMethylation450 BeadChip.

##### Netherlands ALS cohort—Project MinE

The Netherlands ALS cohort was collected under Project MinE [68]. The participants of this study consisted of 1866 Netherlands individuals (1222 ALS cases and 644 control individuals) [69]. All ALS cases were diagnosed with definite or probable ALS according to the revised El Escorial criteria [66], and those with a recorded family history for ALS were excluded. All participants gave written informed consent, and the institutional review board of the University Medical Center Utrecht approved this study. DNA methylation data for the NL sample were measured using Illumina Infinium HumanMethylation450 BeadChip and generated under similar protocols to the AUS cohort.

##### King’s College London ALS cohorts

The King’s College London (KCL) ALS cohort was collected under Project MinE [68]. The participants of this study consisted of a subset of 1433 individuals of UK nationality from the UK National DNA Bank for MND Research who were put forward for DNA methylation profiling. Cases were diagnosed with ALS in one of 20 UK hospitals by neurologists specialized in motor neuron diseases; patients had no family history for ALS and were of self-reported European descent. All cases and controls gave written informed consent. The national Integrated Research Approval System (IRAS) approved the study, reference: 08/H0405/60. DNA was extracted by use of standard methods at three centers within 1 week of the blood being drawn (usually on the same day) and was stored centrally at the UK DNA banking network in Manchester. We used a barcode-based sample tracking system to minimize the risk of clerical error. DNA methylation status of the participants was extracted from whole blood samples using Illumina Infinium HumanMethylation450 BeadChip array following manufacturer’s protocol. These samples were run in two separate batches at two different time points (batch1 *N* = 666; batch2 *N* = 767). Both batches followed the same quality control pipeline and DNA methylation data were normalized together. Since we did not observe major batch effects, we analyze them as a unique cohort.

#### Parkinson’s disease (PD) cohorts

##### System Genomics of Parkinson’s Disease (SGPD) cohort

The SGPD case-control cohort comprises genotype, phenotype and DNA methylation data for a total of 2333 participants (1292 PD cases, 1041 controls) recruited from three different studies across Australia and New Zealand: (1) the Queensland Parkinson’s Project (QPP), (2) the New Zealand Brain Research Institute PD case-control cohort (NZBRI), and (3) the Sydney PD case-control cohort (SYD). The study design, diagnostic criteria, and DNA methylation assays have been described elsewhere [18], but briefly, the QPP cohort includes 1791 participants (867 PD cases, 924 controls), mostly of European ancestry (individuals with genetically confirmed non-European ancestry were excluded prior to analysis). The NZBRI cohort comprises 210 participants (151 PD cases, 59 matched-controls) recruited by the NZBRI. Exclusion criteria for PD patients were prior history of learning disability, severe head injury, stroke, or other neurological impairment and major psychiatric complications at the point of study entry. Whole-blood samples were collected at the same time as phenotypic measurements, which included demographic, medical, and environmental exposure information for all participants. In the SYD cohort, 332 participants (274 PD cases, 57 matched-controls) were recruited from the Parkinson’s Disease Research Clinic, Brain and Mind Research Centre at the University of Sydney. The DNA methylation data were measured using Illumina Infinium HumanMethylation450 BeadChip.

##### Parkinson’s disease, environment, and genes (PEG) cohort

The PEG study is a large population-based study of PD of mostly rural and township residents of California’s central valley [70]. The PEG study comprises of 508 European (289 PD cases, 219 controls) and 63 Hispanic individuals (45 PD cases, 18 controls) for a total of 334 PD cases and 237 controls. Study design, diagnostic criteria, and DNA methylation assays have been described elsewhere [70, 71]. The DNA methylation data measured using Illumina Infinium HumanMethylation450 BeadChip.

#### Alzheimer’s disease (AD) cohorts

##### The Australian Imaging, Biomarkers and Lifestyle (AIBL) cohort

The AIBL cohort is a prospective longitudinal study of aging aimed to recruit 1000 individuals aged over 60 to assist with prospective research into AD. Data was collected by the AIBL study group. AIBL study methodology has been reported previously [72]. Participants with AD (*N* = 211) had neuropsychological profiles which were consistent with AD and were more impaired than participants with mild cognitive impairment (MCI) (*N* = 133) or healthy controls (*N* = 768), who performed within expected norms for age on neuropsychological testing. A subset of the AIBL cohort (*N* = 162 AD cases, *N* = 94 MCI cases, and *N* = 485 controls) were subjected to a similar DNA methylation assay protocol as the AUS ALS cohort [20], but bisulfite DNA samples were hybridized to the 8 sample, HumanMethylationEPIC BeadChip Array.

##### The Alzheimer’s Disease Neuroimaging Initiative (ADNI)

ADNI is a consortium of universities and medical centers in the United States and Canada. They launched in 2003 as a public-private partnership, led by principal investigator Michael W. Weiner, MD. ADNI was established to develop standardized imaging techniques and biomarker procedures in normal subjects, subjects with MCI, and subjects with mild AD [73]. The main goal of ADNI is to characterize cross-sectionally and longitudinally clinical measures in normal controls, subjects with MCI, and subjects with mild Alzheimer disease (AD) to enable the assessment of the utility of neuroimaging and chemical biomarker measures. The study design, enrolment process, neuropsychological assessments, and diagnostic criteria have been previously described elsewhere [73]. Briefly, a total of 819 subjects (229 cognitively normal, 398 with MCI, and 192 with AD) were enrolled at baseline and followed for 12 months using standard cognitive and functional measures typical of clinical trials [73]. Whole-genome DNA methylation profiling was done from blood samples of ADNI participants. DNA was isolated and plated out at NCRAD and DNA methylation profiling was performed at AbbVie Inc. for a total of 1920 samples, including 1719 unique samples and 201 technical replicates (653 unique individuals). Longitudinal DNA samples at baseline, + 1 and + 2 years were obtained from all subjects. The Illumina Infinium HumanMethylationEPIC BeadChip Array was used for methylation profiling. Samples were randomized using a modified incomplete balanced block design, whereby all samples from a subject were placed on the same chip, with remaining chip space occupied by age- and sex-matched samples. Subjects from different diagnosis groups were placed on the same chip to avoid confounding. Unused chip space was leveraged for technical reproducibility assessment via replicated DNA samples.

##### AddNeuroMed—the European collaboration for the discovery of novel biomarkers for Alzheimer’s disease

AddNeuroMed is part of the Innovative Medicines in Europe initiative (InnoMed), and was established with the goal of biomarker discovery for Alzheimer’s disease (AD) [74, 75]. Participants were recruited at six centers throughout Europe (Kuopio, Finland; Łódź, Poland; London, UK; Perugia, Italy; Thessaloniki, Greece; Toulouse, France), following standardized procedures. Ethical approval was obtained at each site, and informed consent was obtained according to the declaration of Helsinki (1991). Recruited participants included individuals with AD diagnosed according to the NINCDS-ADRDA criteria [76], individuals with mild cognitive impairment (MCI) according to Petersen’s criteria [77], and controls who showed no symptoms of dementia and had a mini mental state examination (MMSE) score of 28 or higher. Participants were excluded if they had depression or any other neurological syndrome.

A subset of 301 samples were selected from the cohort for DNA methylation profiling, including 96 controls, 111 individuals with MCI, and 94 AD cases. DNA extraction methods have previously been described elsewhere [15, 78]. DNA methylation was measured using Illumina HumanMethylation450 BeadChip arrays and the Illumina HiScan System. Samples were randomized by sex, diagnostic status, and recruitment center. Clinical data for this cohort can be accessed according to the data terms of use for AddNeuroMed https://www.synapse.org/#!Synapse:syn2790911/wiki/235389.

#### Schizophrenia cohorts

##### University College London schizophrenia cohort (SCZ1)

The University College London schizophrenia case–control sample study design, diagnostic criteria, DNA methylation collection details, and assay characteristics have been thoroughly described elsewhere have been described elsewhere [21, 79], but briefly comprises of unrelated ancestrally matched cases (*N* = 353) and controls (*N* = 322) from the UK. Case participants were recruited from UK National Health Service (NHS) mental health services with a clinical International Classification of Diseases 10th edition (ICD-10) diagnosis of schizophrenia. The DNA methylation data were measured using Illumina Infinium HumanMethylation450 BeadChip.

##### Aberdeen schizophrenia cohort (SCZ2)

The Aberdeen schizophrenia case–control sample study design, diagnostic criteria, DNA methylation collection details and assay characteristics have been thoroughly described elsewhere have been described elsewhere [21, 80], but briefly contains patients with schizophrenia (*N* = 414) and controls (*N* = 433) who have self-identified as born in the British Isles (95% in Scotland). All cases met the Diagnostic and Statistical Manual for Mental Disorders fourth edition (DSM-IV) and ICD-10 criteria for schizophrenia. The DNA methylation data were measured using Illumina Infinium HumanMethylation450 BeadChip.

#### Rheumatoid arthritis cohort

The raw methylation data from Illumina Infinium HumanMethylation450 BeadChip arrays and phenotypic data for rheumatoid arthritis cases and controls were obtained from the publicly available Gene Expression Omnibus submitted dataset GSE42861, which was part of the Epidemiological Investigation of Rheumatoid Arthritis (EIRA) study [81, 82]. Only incident rheumatoid arthritis cases were invited for the study within the years 1996–2009 from middle Sweden. The controls matched by sex, age, smoking status, and residence area were selected from the same population. The cohort and DNA methylation collection details and assay characteristics have been thoroughly described elsewhere [81].

#### Lothian Birth Cohort 1936 (LBC1936)

LBC1936 [83, 84] was used for out-of-sample DNA methylation classification and correlation analyses with protein inflammatory markers. LBC1936 is a cohort comprising individuals born in 1936, who were aged approximately 70 years at recruitment. Whole blood DNA methylation was measured using the measured using Illumina Infinium HumanMethylation450 BeadChip arrays in 1004 participants from samples collected at mean age 70 years. The DNA methylation collection details and assay characteristics have been thoroughly described elsewhere [85, 86].

### Quality control (QC) and normalization of DNA methylation data

Data QC and normalization were conducted using the *meffil* R package [27]. The same pipeline for DNA methylation data processing and QC was applied to all samples. QC threshold parameters (Additional file 1: Supplementary Note) determined samples and DNA methylation sites to exclude prior to normalization. Functional normalization was performed to remove technical variation, as described elsewhere [87]. Briefly, probe intensity quantiles were normalized between samples by fitting linear models with these quantiles to the top principal components of the control probe matrix. After normalization, the most variable probes (*m* = 20,000) were extracted, decomposed into principal components, and each component regressed against slide, chip column, chip row, and sex to test for batch effects. The association detection *p* value threshold was set to 0.01. Sex-chromosome linked probes, probes overlapping with SNPs, and probes with non-unique hybridization and extension were also removed prior analysis, following general masking recommendations described elsewhere [28]. Afterwards, we removed remaining probes with s.d. < 0.02. This decision is justified, because power to detect an association depends in part on the variance between individuals and (standardized) effect sizes. Excluding these DNA methylation sites also reduces the multiple testing burden in MWAS.

### Protein measurements with Olink® inflammation panel, measured at wave 1 of the Lothian Birth Cohort 1936 (LBC1936) and subsequent quality control measures

Plasma was extracted from 1047 blood samples and collected in lithium heparin tubes at mean age 69.8 ± 0.8 years (wave 1). Plasma samples were analyzed using a 92-plex proximity extension assay (Olink® Bioscience, Uppsala Sweden). The proteins assayed comprise the Olink® inflammatory biomarker panel. Briefly, 1 μL of sample was incubated in the presence of proximity antibody pairs linked to DNA reporter molecules. Upon appropriate antigen-antibody recognition, the DNA tails form an amplicon by proximity extension which is quantified by real-time PCR. Data pre-processing was performed by Olink® using NPX Manager software. We retained all protein measures with at least 80% of individuals above the limit of detection (below 3 times standard deviation over background). The remaining 73 proteins were transformed by rank-based inverse normalization prior to analysis, to ensure normally distributed values. One protein from the panel, BDNF, failed quality control and was removed from the study.

### Statistical analyses

Mixed linear model-based omics association (MOA) and multi-component mixed linear model-based omics association excluding the target (MOMENT) MWAS analyses.

One of the most well-recognized challenge in MWAS (and other omics-based analyses) is how to better control the false-positive rate (FPR) in the presence of confounding factors (e.g., cell type proportion, smoking, age, batch effects), since failing to account for their effects may lead to spurious associations [57, 88]. To address this issue, the software OSCA has been recently developed [24]. OSCA implements two reference-free mixed-linear model approaches: MOA and MOMENT. Briefly, the MOA method fits a random genome-wide DNA methylation factor per person with variance-covariance matrix between individuals built from genome-wide DNA methylation sites (equivalent to a model of fitting all DNA methylation sites as random effects); this model is analogous to the MLM association method implemented in EMMAX [89] and GCTA [90] for SNP data. The MOMENT method fits an MLM with two random-effect components for each probe tested with the DNA methylation sites grouped by their associations with the trait (leaving out the DNA methylation sites in a window around the target probe being tested for association) [24], allowing a different genome-wide architecture of DNA methylation compared to MOA; in MOMENT DNA methylation effect sizes genome-wide are drawn from two distributions with the variances of the distributions estimated from the data. Both methods have been shown, through extensive simulations [24], to have lower FPR than other methods. MOMENT has slightly less power compared to MOA when a single distribution of effect sizes is appropriate for the trait under study. We conducted mixed-linear model MWAS using both MOA and MOMENT [24]. The MOA MWAS model is:

1 $$ y={w}_i{b}_i+ Wu+\mathrm{e} $$

where *y* is an *n* × 1 vector of phenotype values of *n* individuals, w_i_ (a *n × 1* vector of DNA methylation measures (β values) of a probe *i*, i.e., the target probe) and *b*_*i*_ (the effect of probe *i* on the phenotype; fixed effect), *W* is an *n x m* matrix of m standardized DNA methylation values, where *m* is the number of DNA methylation sites, *u* is an *m × 1* vector of the joint random probe effects on the phenotype, and *e* is an *n × 1* vector of residuals. The variance of y is var.(y) = $\mathrm{V}={\mathrm{WW}}^{\prime }{\upsigma}_{\mathrm{u}}^2+\mathrm{I}{\upsigma}_{\mathrm{e}}^2$ . We can re-write this equation as $V=A{\upsigma}_o^2+\mathrm{I}{\upsigma}_{\mathrm{e}}^2$ with *A* = WW^′^/*m* and ${\upsigma}_o^2=\mathrm{m}{\upsigma}_u^2$, where *A* is then the omics-data-based relationship matrix and ${\upsigma}_u^2$ is the variance between individuals attributed to genome-wide DNA methylation differences. The null hypothesis (*H*_0_: *b*_*i*_ = 0) can then be tested by a two-sided t test given ${\hat{b}}_i$ and its s.e. The variance components can be estimated by REML.

In this model, the probe being tested is fitted twice, once as a fixed and also as a random effect, which results in slightly reduced power compared to a (hypothetical) model in which the focal probe is excluded from W, but this would be computationally very demanding. It is also assumed that all probe effects follow a single distribution, which may not reflect the true distribution. In the MOMENT model, DNA methylation probe effect sizes are drawn from two effect size distributions for different probes sets, selected according to their association statistics in an initial linear regression model, with each group then fitted as a random-effect:

2 $$ \mathrm{y}={w}_i{b}_i+\sum \limits_{\mathrm{j}}{\mathrm{W}}_{\mathrm{j}}{\mathrm{u}}_{\mathrm{j}}+\mathrm{e} $$

where *W*_j_ is an *n x m*_*j*_ matrix of standardized DNA methylation probe values in the *j*th group, and *m*_*j*_ is the number of DNA methylation sites in the group (excluding the DNA methylation sites in a 100 Kb region of centered at the probe to be tested). When the number of probes in the first group is too large, the analysis becomes slow because the program needs to re-estimate the variance components whenever one or more probes are removed from the first group (to avoid proximity contamination) [24]. This may also cause convergence problem because of too much variation explained by the first random-effect component. However, OSCA implements a version of MOMENT where an additional stepwise selection procedure to reduce the number of probes in the first group. Simulation shows that this method has approximately the same level of false positive rate, but slightly higher power compared to the initial MOMENT implementation (https://cnsgenomics.com/software/osca/#EWAS). We thus performed MWAS for the AUS, NL, KCL, SGPD, PEG, AIBL, SCZ1, SCZ2, and rheumatoid arthritis cohorts separately and for consistency always used the *--moment2-beta* function for MOMENT analyses, as implemented in the OSCA software. DNA methylation sites were then mapped to the latest GRCh38/hg38 genome build [28] and annotated to genes, based on GENCODE v22.

#### Meta-analyses of MOMENT MWAS within and between neurodegenerative disorders to identify DMPs

We conducted inverse-variance weighted meta-analyses using the MOMENT results from each individual cohort, using METAL [91]. We only kept probes in common between all datasets in the analyses. We performed meta-analyses within ALS (AUS, NL, and KCL cohorts, *N*_cases_ = 3035, *N*_controls_ = 1524), within PD (SGPD and PEG, *N*_cases_ = 1133, *N*_*controls*_ = 998), and between neurodegenerative disorders (ALS, PD, and AD, *N*_cases_ = 4329, *N*_controls_ = 2993). We conducted meta-analyses to identify shared DMPs using MOMENT results, since this method as shown to be more robust to (un)observed confounders [24].

#### Meta-analyses of publicly available meta-GWAS summary statistics between neurodegenerative disorders

To assess the potential overlap of the MWAS meta-analyses results with methylation quantitative trait loci (mQTL) and GWAS SNPs, we first performed a meta-analysis of three publicly available meta-GWAS summary statistics, for AD (*N* = 368,440) [38], ALS (*N* = 80,610) [39], and PD (minimum *N* = 520, maximum *N* = 482,730, excluding 23andMe SNPs) [40]. We conducted sample-size weighted meta-analyses, using METAL [91], for all SNPs in common between datasets. Sex-linked SNPs, SNPs with minor allele frequency (MAF) < 0.01, and SNPs with incongruent MAF or base pair position between datasets were excluded.

#### IGAP summary statistics

When looking for overlap with GWAS signals, we analyzed summary statistics provided by the International Genomics of Alzheimer’s Project (IGAP), in addition to the summary statistics from the GWAS above. IGAP is a large three-stage study based upon GWAS on individuals of European ancestry. In stage 1, IGAP used genotyped and imputed data on 11,480,632 single nucleotide polymorphisms (SNPs) to meta-analyze GWAS datasets consisting of 21,982 Alzheimer’s disease cases and 41,944 cognitively normal controls from four consortia: The Alzheimer Disease Genetics Consortium (ADGC); The European Alzheimer’s disease Initiative (EADI); The Cohorts for Heart and Aging Research in Genomic Epidemiology Consortium (CHARGE); and The Genetic and Environmental Risk in AD Consortium Genetic and Environmental Risk in AD/Defining Genetic, Polygenic and Environmental Risk for Alzheimer’s Disease Consortium (GERAD/PERADES). In stage 2, 11,632 SNPs were genotyped and tested for association in an independent set of 8362 Alzheimer’s disease cases and 10,483 controls. Meta-analysis of variants selected for analysis in stage 3A (*n* = 11,666) or stage 3B (*n* = 30,511) samples brought the final sample to 35,274 clinical and autopsy-documented Alzheimer’s disease cases and 59,163 controls.

#### Multiple logistic regression to estimate disorder-specific effect sizes of predicted cell-type proportions (CTP)

We used the EpiDISH algorithm [47] to predict DNA methylation-derived proportions of B lymphocytes (Bcell), CD4+ lymphocytes (CD4T), CD8+ T lymphocytes (CD8T), eosinophils (Eosino), monocytes (Mono), neutrophils (Neu), and natural killer cells (NK). We then used multiple logistic regression models with case-control status (excluding MCI) as response variable, to estimate effect sizes of CTPs associated with each disorder. Predicted CTP, predicted DNA methylation age [48], DNA methylation-derived smoking scores [23], and study site were included as covariates in the models. We summed the Neu and Eosino proportions, since these are biologically classified as granular leukocytes. We excluded CD8T proportions from analyses due to redundancy in proportion data. Prior fitting the models, we excluded outlying CTP values that were larger than *mean*(*CTP*) ± 3 × *SD*(*CTP*) (*N* = 524, including MCI), as such extreme CTP may be indicative of current sickness in these elderly participants. A summary of the models can be found in (Additional file 2: Table S7). To fit the regression models, we used the *glm* function in the *stats* R package, with a binomial error distribution and logit link function. We calculated Wald 95% confidence intervals for the exponentiated log odds using the *confint.default* function in the *MASS* R package [92].

#### Out-of-sample classification using cell-type-proportion (CTP) and DNA methylation profile scores (MPS)

A profile score is calculated for each individual in the target sample as the sum of CTP or DNA methylation values (MPS) weighted by their effect sizes, estimated in a discovery sample. Classification efficacy of the profile scores was evaluated by the area under the receiver-operator characteristic curve (AUC) that relates the false positive rate (specificity) to the true positive rate (sensitivity), from logistic regression, with case-control status as dependent variable and MPS or CTP-scores as independent variable. We used the R package *pROC* [93] to plot the receiver-operator characteristic curves and calculate AUC for each profile score. The CI_95%_ for the AUC was calculated using the *ci.auc* function, using the *DeLong* method. We conducted out-of-sample classification using the MOA/MOMENT results of the AUS, SGPD, SCZ1, and AIBL cohorts, as discovery samples. We conducted both within-trait and cross-trait classification. We calculated MPS using DNA methylation probes that passed significance at the following *p* value thresholds: *p* < 0.5, *p* < 0.2, *p* < 0.1, *p* < 1 × 10^−2^, *p* < 1 × 10^−3^, *p* < 1 × 10^−4^, *p* < 1 × 10^−5^. We only kept probes in common between all cohorts in the analyses. The CTP effect sizes, used to calculate the CTP-scores were estimated from multiple logistic regression models, as described above.

#### Correlation of CTP-scores and MPS in the Lothian Birth Cohort 1936 (LBC1936), a healthy aging cohort

To assess if disease associated CTP-scores and MPS were capturing inflammation signals, we calculated correlations with blood protein inflammatory markers, as measured by the Olink® panel, in the LBC1936, a healthy aging cohort (see above). We calculated MPS using DNA methylation probes effect sizes from the MOA/MOMENT meta-analyses of neurodegenerative disorders (AD, ALS, and PD). Additionally, we calculated disease-associated CTP-scores, with effect sizes estimated from multiple logistic regression models, described above. As before, we excluded outlying CTP values in the LBC1936 that were larger than *mean*(*CTP*) ± 3 × *SD*(*CTP*) (*N* = 46). Finally, we calculated an inflammation-related profile score for each individual in the LBC1936, as described by Barker et al. [49]. Briefly, CRP-related probes were selected based on a recent methylome-wide association study by Ligthart et al. [50]. This selection was limited to 7 CpG probes (spanning a total of 9 genes) that showed the strongest evidence for a functional association with CRP levels, including cg06126421 (standardized effect size = − 0.0052), cg06690548 (− 0.0048), cg10636246 (− 0.0069), cg18181703 (− 0.0053), cg19821297 (− 0.0051), cg25325512 (− 0.0031), and cg27023597 (− 0.005). All analyses were conducted in R version 3.6.0, Rstudio v1.2.1335, and OSCA version 0.45.

## Supplementary Information

**Additional file 1.** Supplementary notes and supplementary figures.

**Additional file 2.** Supplementary tables.

**Additional file 3.** Review history.

## Data Availability

All code used for analyses and generate figures in this manuscript can be found at https://github.com/m-nabais/202001_cross_disorder_DNA methylation and Zenodo [94]. The DNA methylation data sets are available as follows: • Australian ALS cohort: The DNA methylation data have been submitted to dbGAP under accession number: phs002068.v1.p1, excluding the OATS samples (*N* = 84) [20]. For OATS samples, data are available to researchers by request to the OATS Governance Committee. • SGDP Parkinson’s cohort: The DNA methylation data are publicly available at GEO under accession number GSE145361 [95]. • PEG Parkinson’s cohort: The DNA methylation data are publicly available at GEO under accession number GSE111629 [96]. • KCL ALS cohort: The DNA methylation data have been submitted to EGA under accession number: EGAC00001000703 [97]. • Netherlands ALS cohort: The DNA methylation data have been submitted to EGA under accession number: EGAC00001000703 [97]. • AIBL cohort: The DNA methylation data are publicly available at GEO under accession number GSE153712 [98]. • ADNI cohort: The DNA methylation data are available to researchers by request as outlined in the ADNI access policy [73]. • AddNeuroMed cohort: The DNA methylation data are publicly available at GEO under accession number GSE144858 [99]. • UCL schizophrenia cohort: The DNA methylation data are publicly available at GEO under accession number GSE80417 [100]. • Aberdeen schizophrenia cohort: The DNA methylation data are publicly available at GEO under accession number GSE84727 [101]. • Epidemiological Investigation of Rheumatoid Arthritis (EIRA): The DNA methylation data are publicly available at GEO under accession number GSE42861 [102]. • Lothian Birth Cohort: The DNA methylation data are available to researchers by request as outlined in the Lothian Birth Cohort access policy [84].
