# Supplementary material for: Meta-analysis of genome-wide DNA methylation identifies shared associations across neurodegenerative disorders
Source: Genome Biol. 2021 Mar 26;22:90. doi: 10.1186/s13059-021-02275-5 (PMC8004462; doi:10.1186/s13059-021-02275-5)
Supplement: Supplementary file 1 — Additional file 1. Supplementary notes and supplementary figures. [file 13059_2021_2275_MOESM1_ESM.docx]

­**Nabais et al -** Meta-analysis of genome-wide DNA methylation identifies shared associations between neurodegenerative disorders

Table of Contents

[Supplementary Note - QC parameters for exclusion of samples and probes. 3](#_Toc53741641)

[Supplementary Note - Sample characteristics: distribution of predicted age, predicted smoking scores and sex in each cohort 4](#_Toc53741642)

[Fig. S1 4](#_Toc53741643)

[Meta-analysis of MOMENT mixed-linear model association studies, identifies differentially methylated positions between neurodegenerative disorders 5](#_Toc53741644)

[Fig. S2 5](#_Toc53741645)

[Fig. S3 6](#_Toc53741646)

[Fig. S4 7](#_Toc53741647)

[Fig. S5 8](#_Toc53741648)

[Fig. S6 9](#_Toc53741649)

[Fig. S7 10](#_Toc53741650)

[GWAS signals do not overlap with loci centered at the 12 differentially methylated positions 11](#_Toc53741651)

[Fig. S8 11](#_Toc53741652)

[Fig. S9 12](#_Toc53741653)

[Fig. S10 13](#_Toc53741654)

[Fig. S11 14](#_Toc53741655)

[Fig. S12 15](#_Toc53741656)

[Fig. S13 16](#_Toc53741657)

[Fig. S14 17](#_Toc53741658)

[Out-of-sample classification accuracy between disorders from DNA methylation-derived profile scores (MPS) 18](#_Toc53741659)

[Fig. S15 18](#_Toc53741660)

[Analysis of DNAm-derived immune cell-type proportions 19](#_Toc53741661)

[Fig. S16 19](#_Toc53741662)

[Fig. S17 20](#_Toc53741663)

[Fig. S18 21](#_Toc53741664)

[Significant correlations of CTP profile scores with blood protein markers of inflammation in the Lothian Birth Cohort 1936 (LBC36), but not with MOMENT profile scores 22](#_Toc53741665)

[Fig. S19 22](#_Toc53741666)

[Fig. S20 23](#_Toc53741667)

[Fig. S21 24](#_Toc53741668)

[Fig. S22 25](#_Toc53741669)

[Fig. S23 26](#_Toc53741670)

[Fig. S24 27](#_Toc53741671)

[Fig. S25 28](#_Toc53741672)

[Supplementary Note - Literature evidence for functional role of 12 DMPs in MOMENT meta-analysis of neurodegenerative disorders 29](#_Toc53741673)

# Supplementary Note - QC parameters for exclusion of samples and probes.

1. Exclude DNAm sites with low bead numbers in high proportion of samples (proportion of samples with bead number < 3 is > 0.1);
2. Exclude DNAm sites with only background signal in high proportion of samples (proportion of samples with detection p > 0.01 is > 0.1);
3. Exclude samples with high proportion of undetected DNAm sites (proportion of DNAm sites with detection p > 0.01 is > 0.1);
4. Exclude samples with high proportion of DNAm sites with low bead number (proportion of DNAm sites with bead number < 3 is > 0.1);
5. Regress median methylated signal on median unmethylated signal and exclude samples whose median methylated signal exceeded 3 standard deviations (SD) from the predicted values;
6. Exclude samples whose control DNAm sites mean value exceeded 5 SD from the mean across all DNAm sites;
7. The median intensity methylated vs unmethylated signal for all control DNAm sites exceeded 3 SD;
8. Calculate difference between median chromosome Y and chromosome X probe intensities (“XY diff”). Cutoff for sex differentiation was “XY diff” = -2. Exclude samples whose XY diff is higher than std = 5 (sex outliers)

# Supplementary Note - Sample characteristics: distribution of predicted age, predicted smoking scores and sex in each cohort

The overall mean age of all participants was 61.4 years (s.d. = 13.4) with 46% of the participants being females. There were statistically significant differences (Mann-Whitney) between the mean DNAm-derived predicted age [1] of cases and controls, for most cohorts (except the Australian ALS cohort (AUS), King’s College ALS cohort (KCL), Aberdeen schizophrenia cohort (SCZ2) and rheumatoid arthritis (RA)), although the density plots do not show major differences between cases and controls, except for the AIBL cohort (**Fig. S1A; Table S2**). We did not find any statistically significant differences between the mean DNAm-derived smoking scores [2] of cases and controls except for the University College London schizophrenia cohort (SCZ1) (U = 19,656, p = 4.84x10^-48^), SCZ2 (U = 43,489, p = 1.47x10^-31^), with SCZ cases showing average higher smoking scores than controls and SGPD (U = 392,404, 1.79x10^-9^), with PD cases showing average lower smoking scores than controls (**Fig. S1B)**.


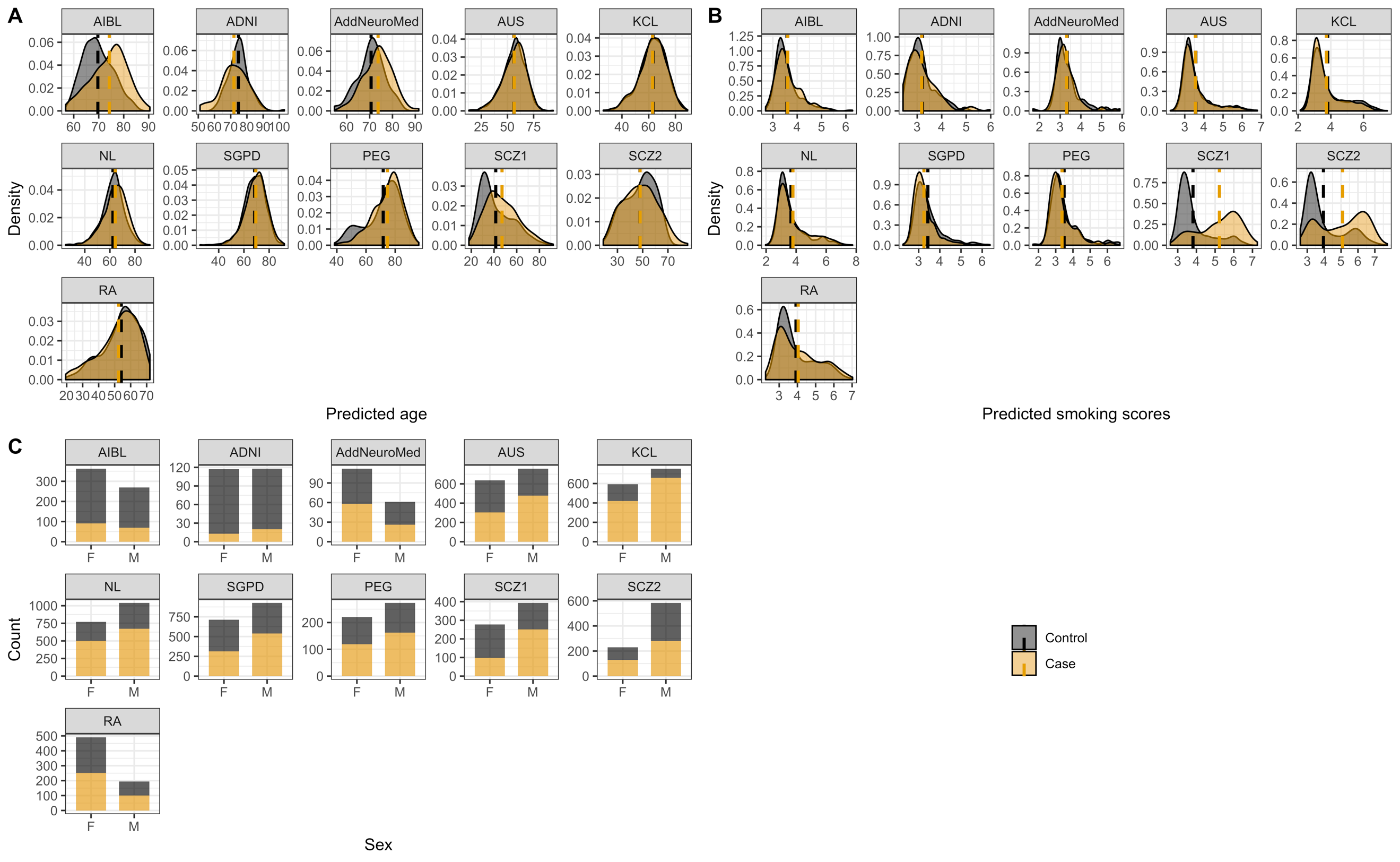


**Fig. S1 - Descriptive statistics for all cohorts available for analysis. A)** Predicted age density plots for cases (orange) and controls (gray); **B)** DNA methylation-derived smoking scores density plots for cases (orange) and controls (gray); **C)** Barplots of individual counts of females (F) and males (M) that were cases (orange) or controls (gray). Orange and gray vertical dashed lines in A) and B) mark the mean value of cases and controls, respectively.

# Meta-analysis of MOMENT mixed-linear model association studies, identifies differentially methylated positions between neurodegenerative disorders


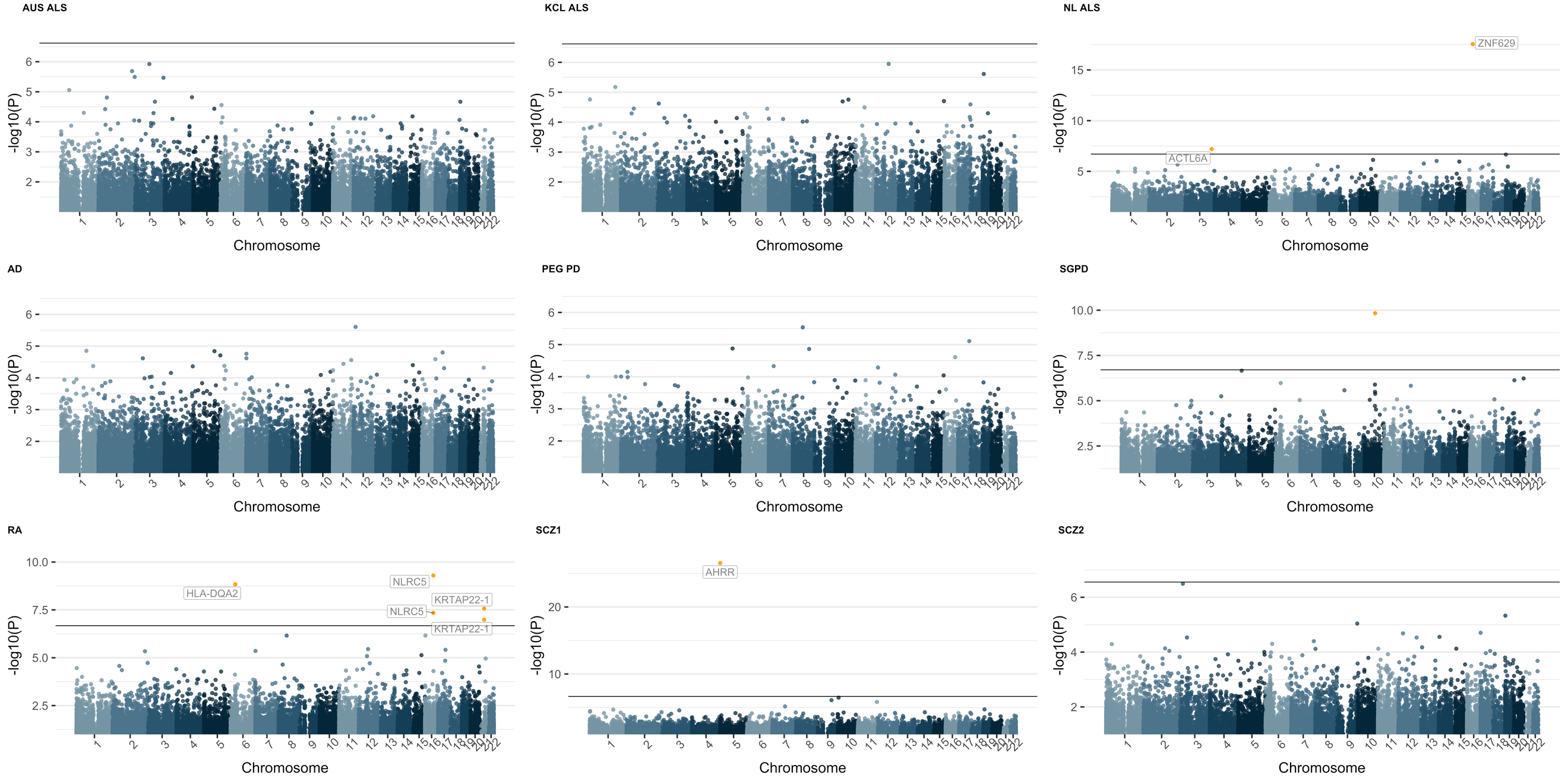


**Fig. S2 - Manhattan plots of MOMENT MWAS for each case-control cohort.** ALS - amyotrophic lateral sclerosis, AD (AIBL) - Alzheimer’s disease, PD - Parkinson’s disease, RA - rheumatoid arthritis, SCZ - schizophrenia.The solid black line refers to the genome-wide significant p-value threshold. For sample sizes, please refer to Figure 1 in the main manuscript. $\lambda$ is the genomic inflation factor (the median of $\chi^{2}$ test-statistics of all DNAm sites divided by its expected value under the null).


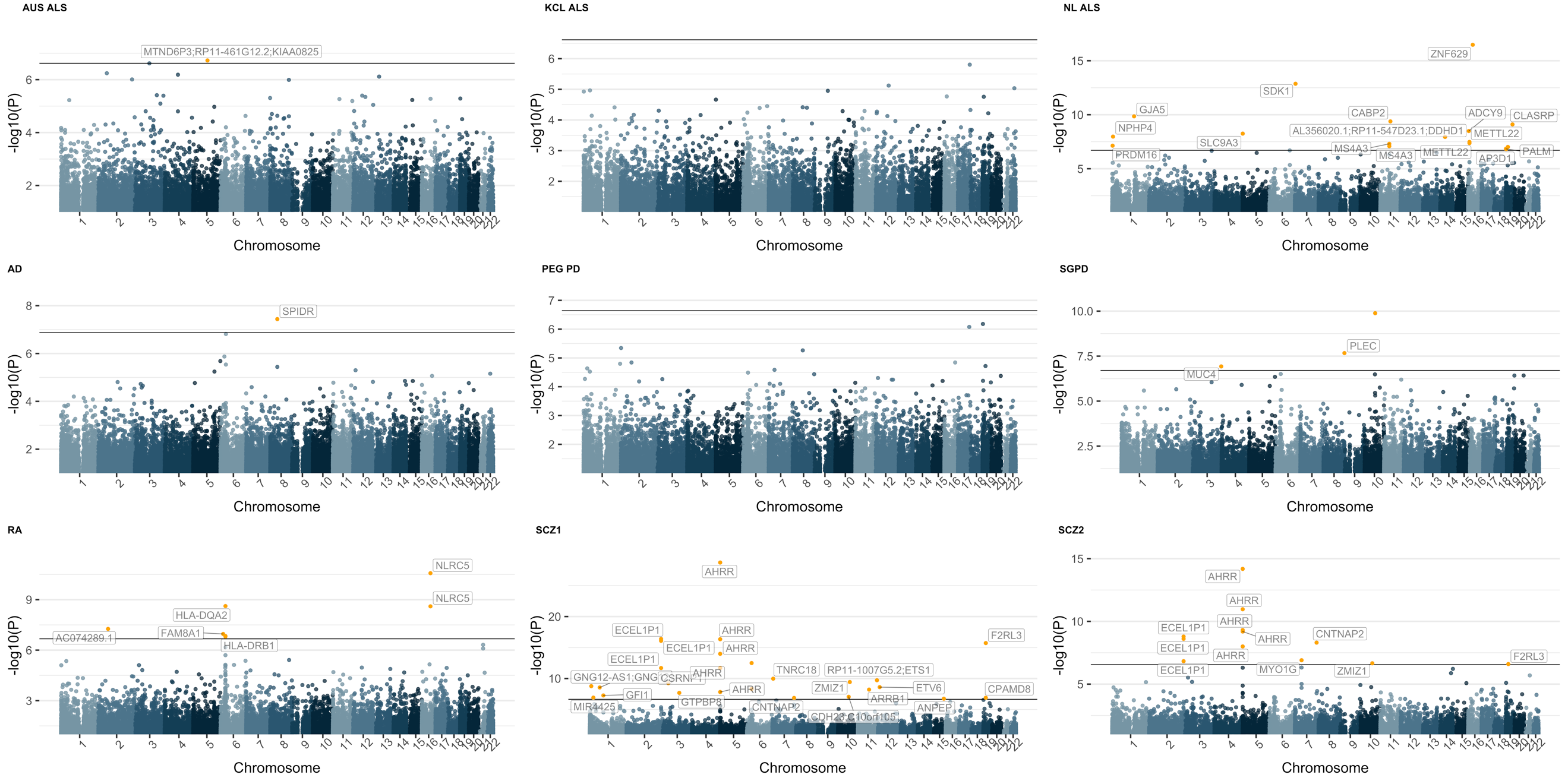


**Fig. S3 - Manhattan plots of MOA MWAS for each case-control cohort.** ALS - amyotrophic lateral sclerosis, AD (AIBL) - Alzheimer’s disease, PD - Parkinson’s disease, RA - rheumatoid arthritis, SCZ - schizophrenia. The solid black line refers to the genome-wide significant p-value threshold. For sample sizes, please refer to Figure 1 in the main manuscript. $\lambda$ is the genomic inflation factor (the median of $\chi^{2}$ test-statistics of all DNAm sites divided by its expected value under the null).


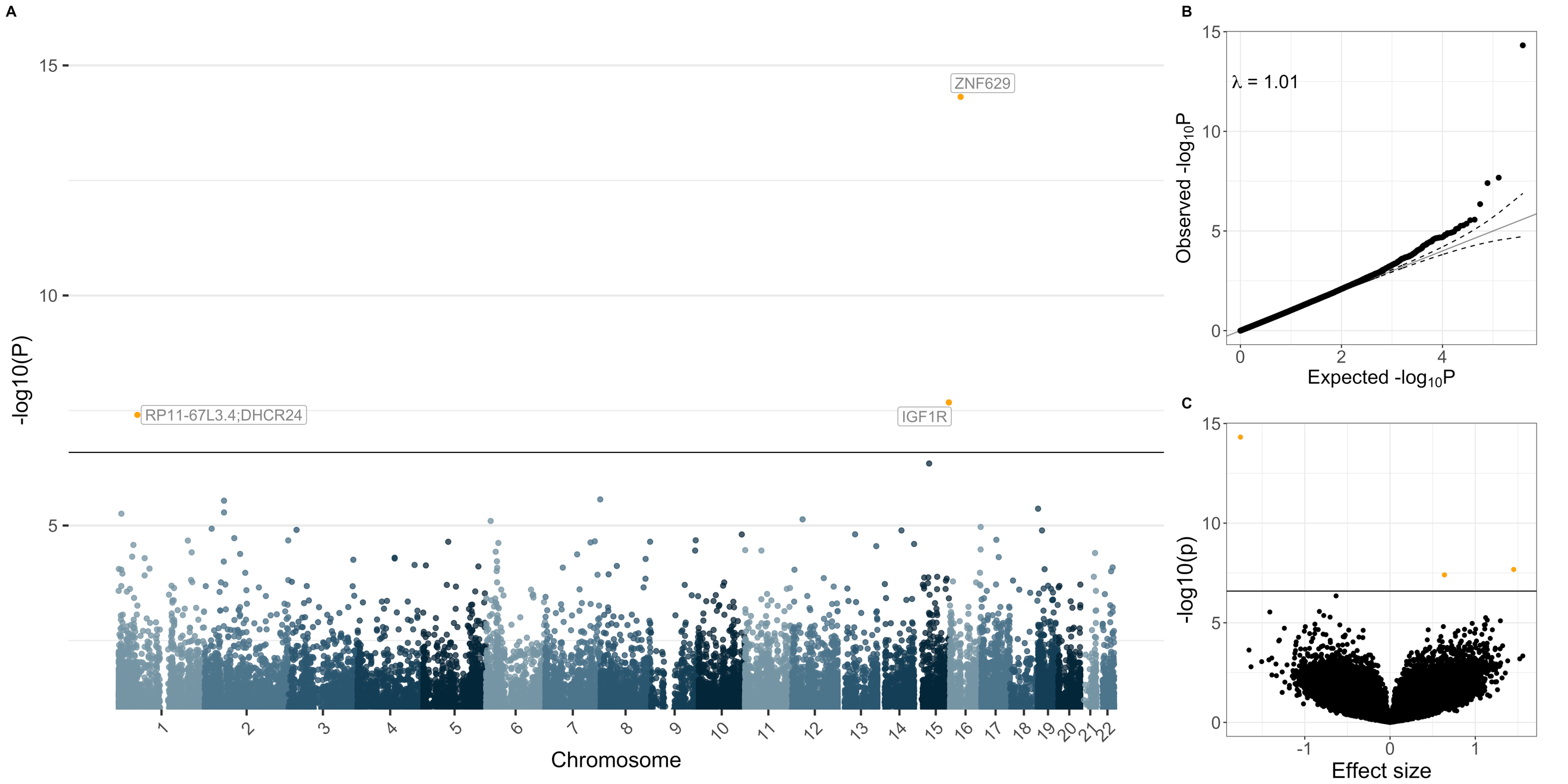


**Fig. S4 - Manhattan (A), Quantile-Quantile (B) and Volcano-plots (C) of the MOMENT meta-analysis of the three amyotophic lateral sclerosis (ALS) cohorts (N_cases_ = 3,032, N_controls_ = 1,522).** The solid black line in A) and C) refers to the genome-wide significant p-value threshold (p = 2.57x10^-7^). The dashed lines in B) mark the upper and lower confidence intervals at 95%, for the p-values. $\lambda$ is the genomic inflation factor (the median of $\chi^{2}$ test-statistics of all DNAm sites divided by its expected value under the null).


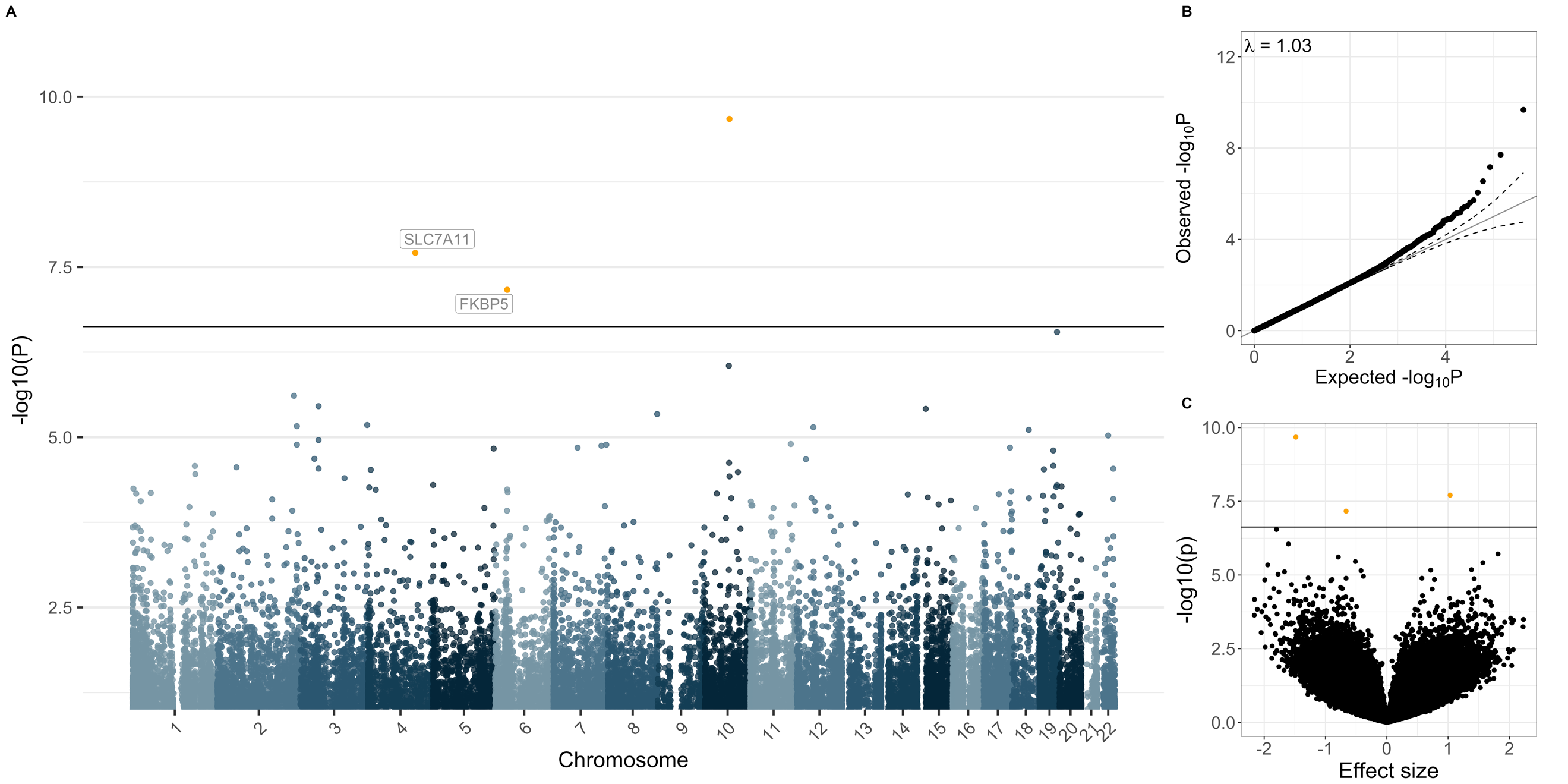


**Fig. S5** - **Manhattan (A), Quantile-Quantile (B) and Volcano-plots (C) of the MOMENT meta-analysis of the three Parkinson’s disease (PD) cohorts (N_cases_ = 1,132, N_controls_ = 999).** The solid black line in A) and C) refers to the genome-wide significant p-value threshold (p = 2.37x10^-7^). The dashed lines in B) mark the upper and lower confidence intervals at 95%, for the p-values. $\lambda$ is the genomic inflation factor (the median of $\chi^{2}$ test-statistics of all DNAm sites divided by its expected value under the null).


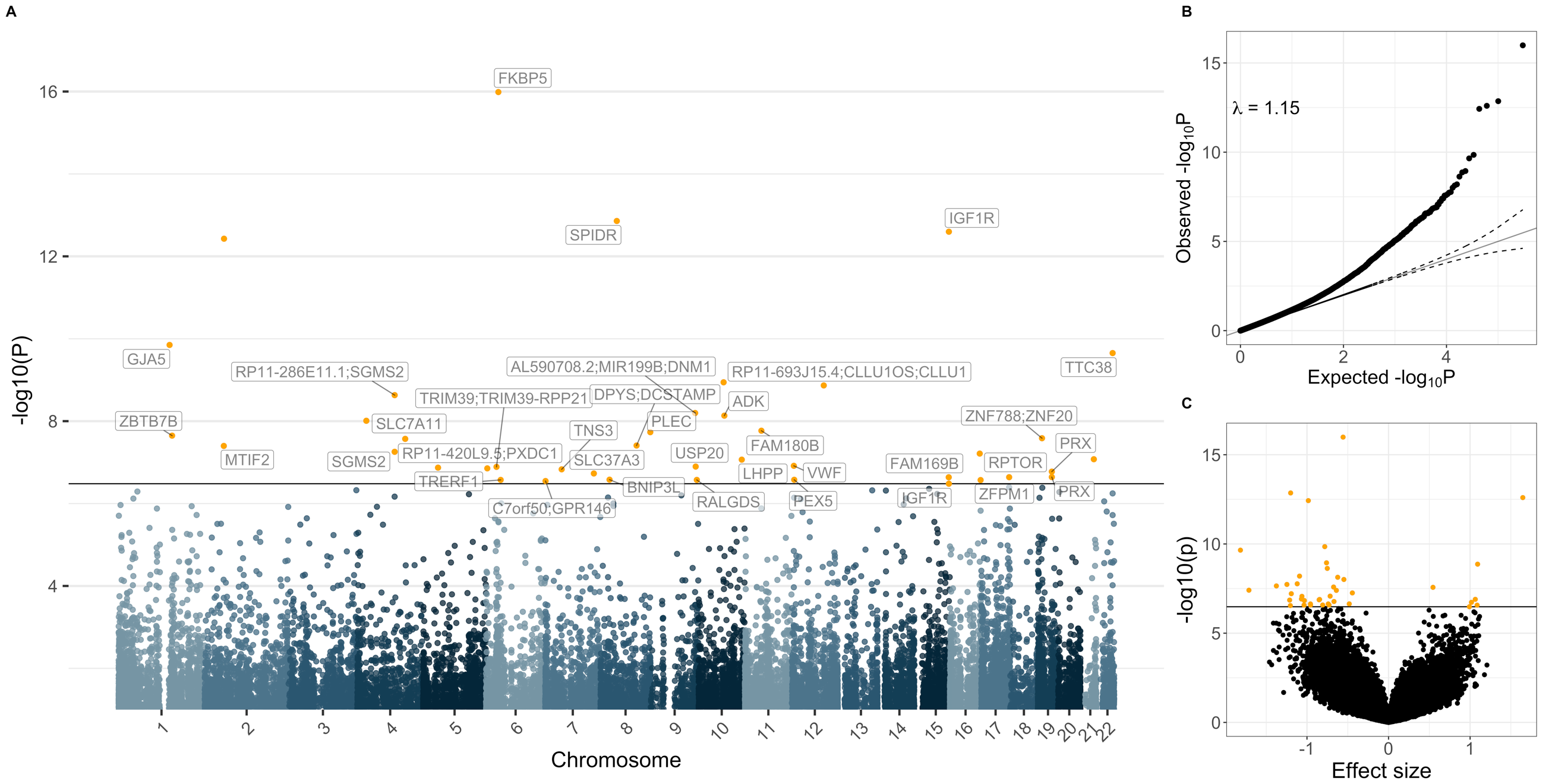


**Fig. S6 - Manhattan (A), Quantile-Quantile (B) and Volcano-plots (C) of the MOA meta-analysis, between ALS, PD and AD cohorts (N_cases_ = 4,325, N_controls_ = 2,992).** The solid black line in A) and C) refers to the genome-wide significant p-value threshold (p = 3.3x10^-7^). The dashed lines in B) mark the upper and lower confidence intervals at 95%, for the p-values. $\lambda$ is the genomic inflation factor (the median of $\chi^{2}$ test-statistics of all DNAm sites divided by its expected value under the null).


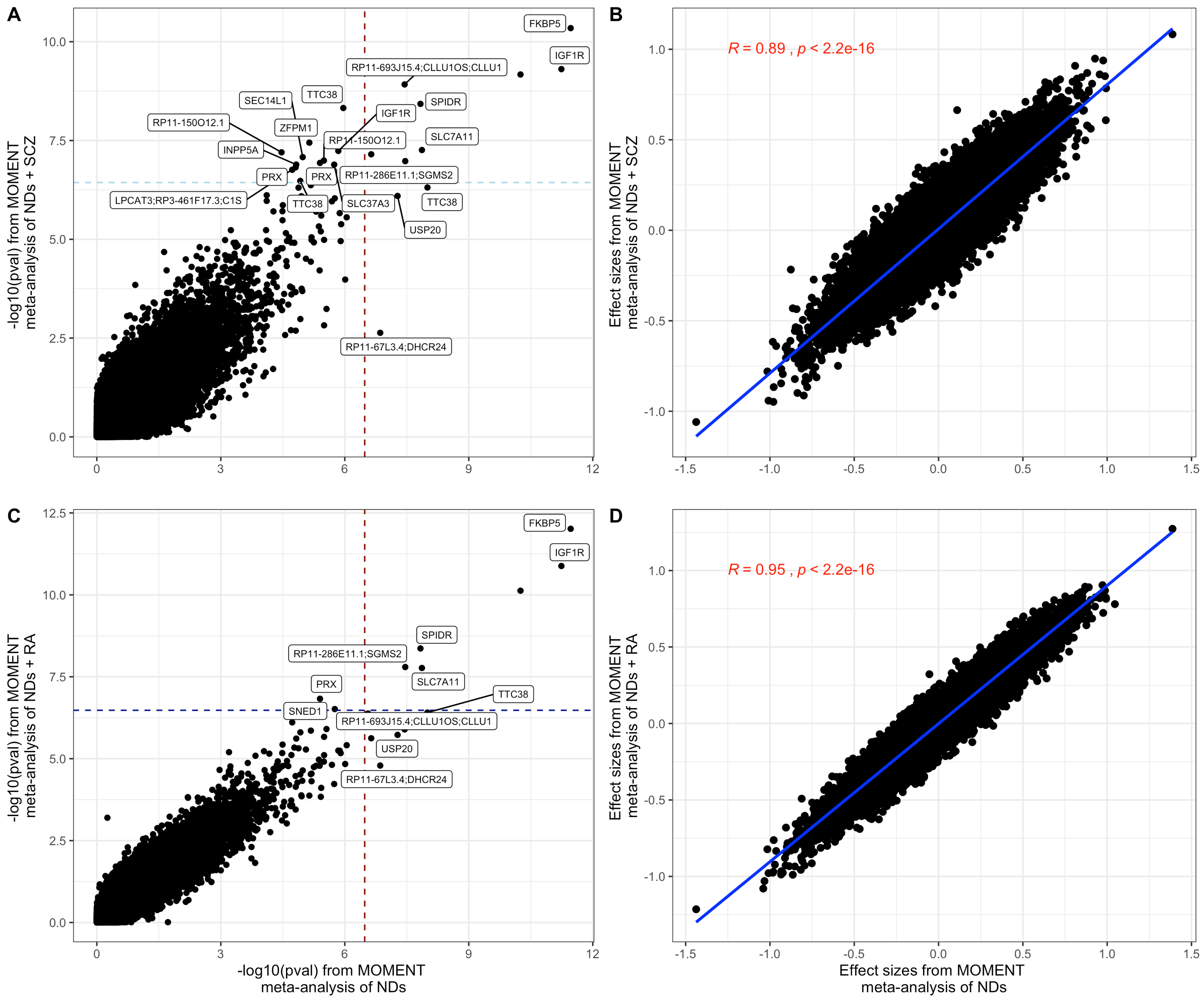


**Fig. S7 - Correlation of -log10(p-values) (left-column) and effect sizes (right-column) of MOMENT meta-analysis results of the three neurodegenerative disorders (x-axis) vs MOMENT meta-analyses adding the two schizophrenia cohorts (A, B) or rheumatoid arthritis (C, D).** The Pearson correlation coefficient and respective p-value is shown in red in B and D. The red, light blue and dark blue dashed lines in B and D mark the Bonferroni corrected genome-wide significance threshold of the MOMENT meta-analysis of the three neurodegenerative disorders (p = 3.30x10^-7^), SCZ (p = 3.66x10^-7^) and RA (p = 3.33x10^-7^), respectively.

# GWAS signals do not overlap with loci centered at the 12 differentially methylated positions

**
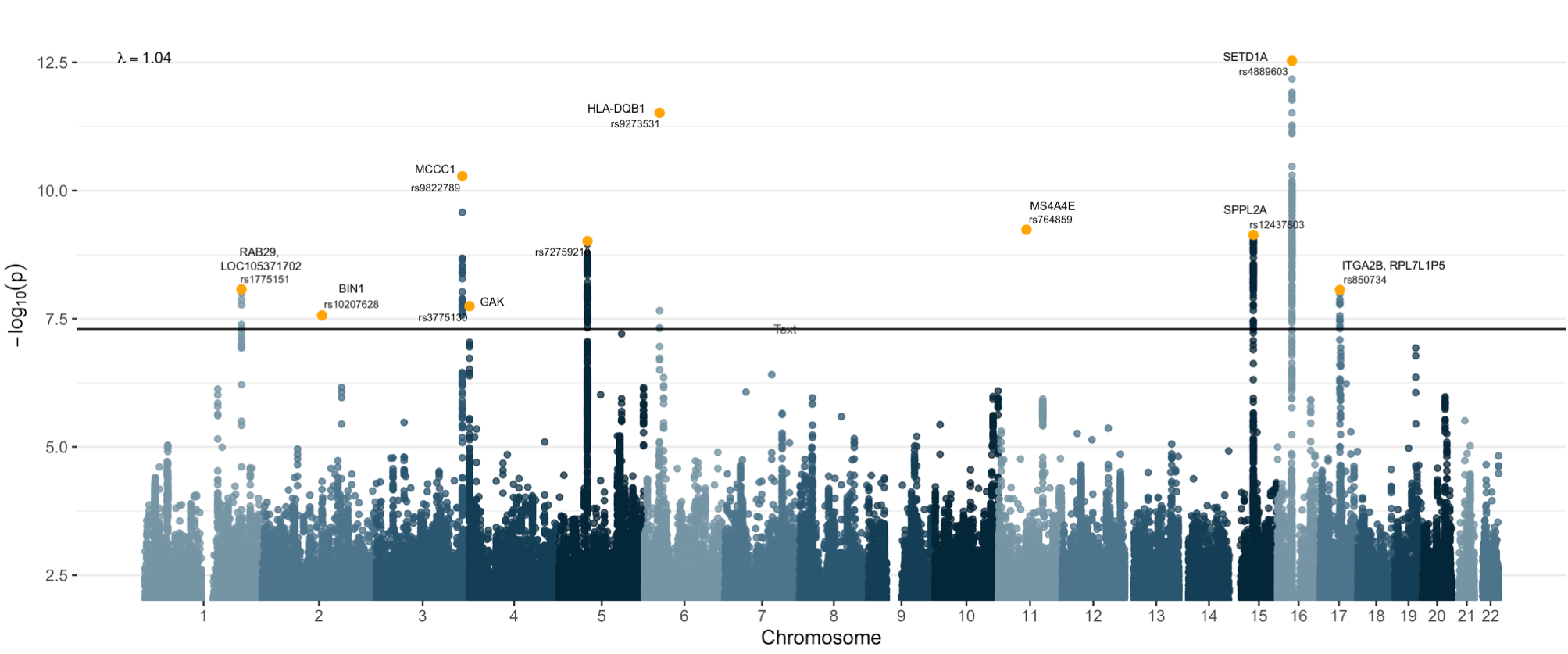
**

**Fig. S8** - Manhattan plot of GWAS results from meta-analysis of publicly available GWAS summary statistics for AD (N = 368,440) [3], ALS (N = 80,610) [4] and PD (minimum N = 520, maximum N = 482,730, excluding 23andMe SNPs) [5]. The solid black line refers to the genome-wide significance p-value threshold (p = 5x10^-8^). $\lambda$ is the genomic inflation factor (the median of $\chi^{2}$ test-statistics of all DNAm sites divided by its expected value under the null). The top SNPs passing genome-wide significance are annotated and highlighted in orange. Depicted in the graph are sites that showed heterogeneity p-values > 0.05 and GWAS -log_10_(p-value) > 2.


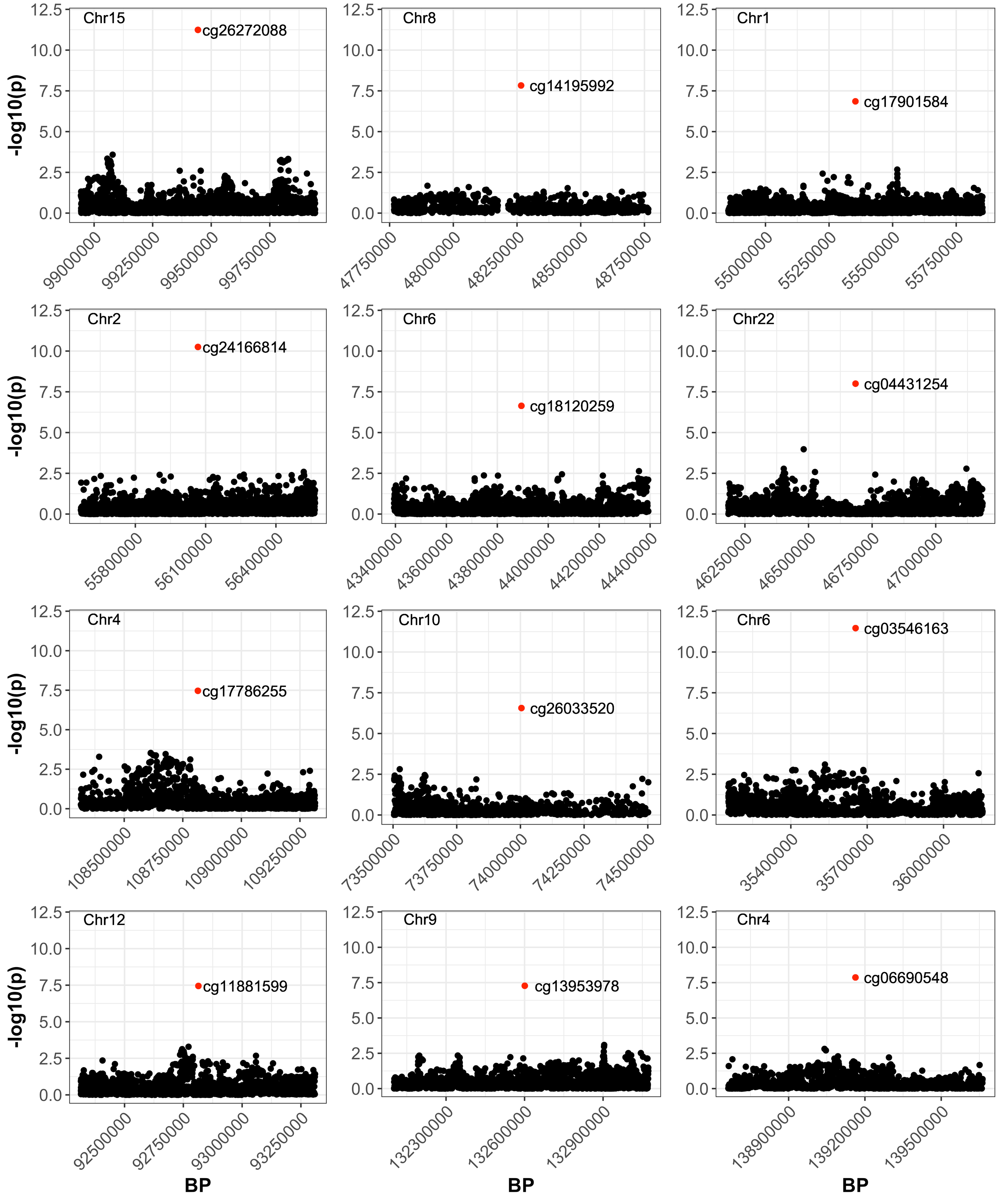


**Fig. S9** - Overlap of the AD GWAS p-values from Marioni et al. 2018 [3] (black dots) and p-values from differentially methylated positions from the DNA methylation meta-analysis of AD, PD and ALS (red dots). Loci shown are ± 500kb from each CpG.


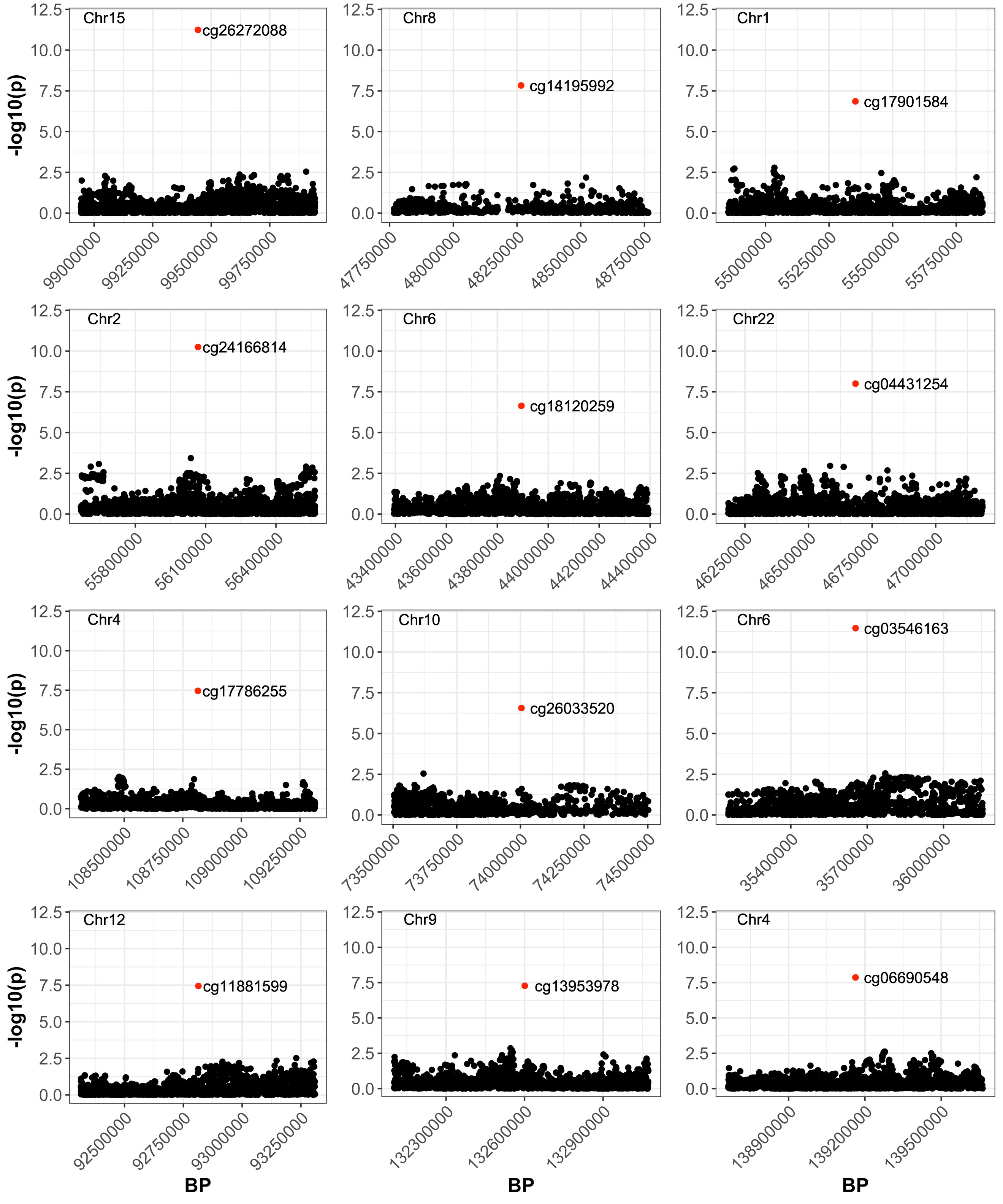


**Fig. S10** - Overlap of the PD GWAS p-values from Nalls et al. 2019 [5] (black dots) and p-values from differentially methylated positions from the DNA methylation meta-analysis of AD, PD and ALS (red dots). Loci shown are ± 500kb from each CpG.


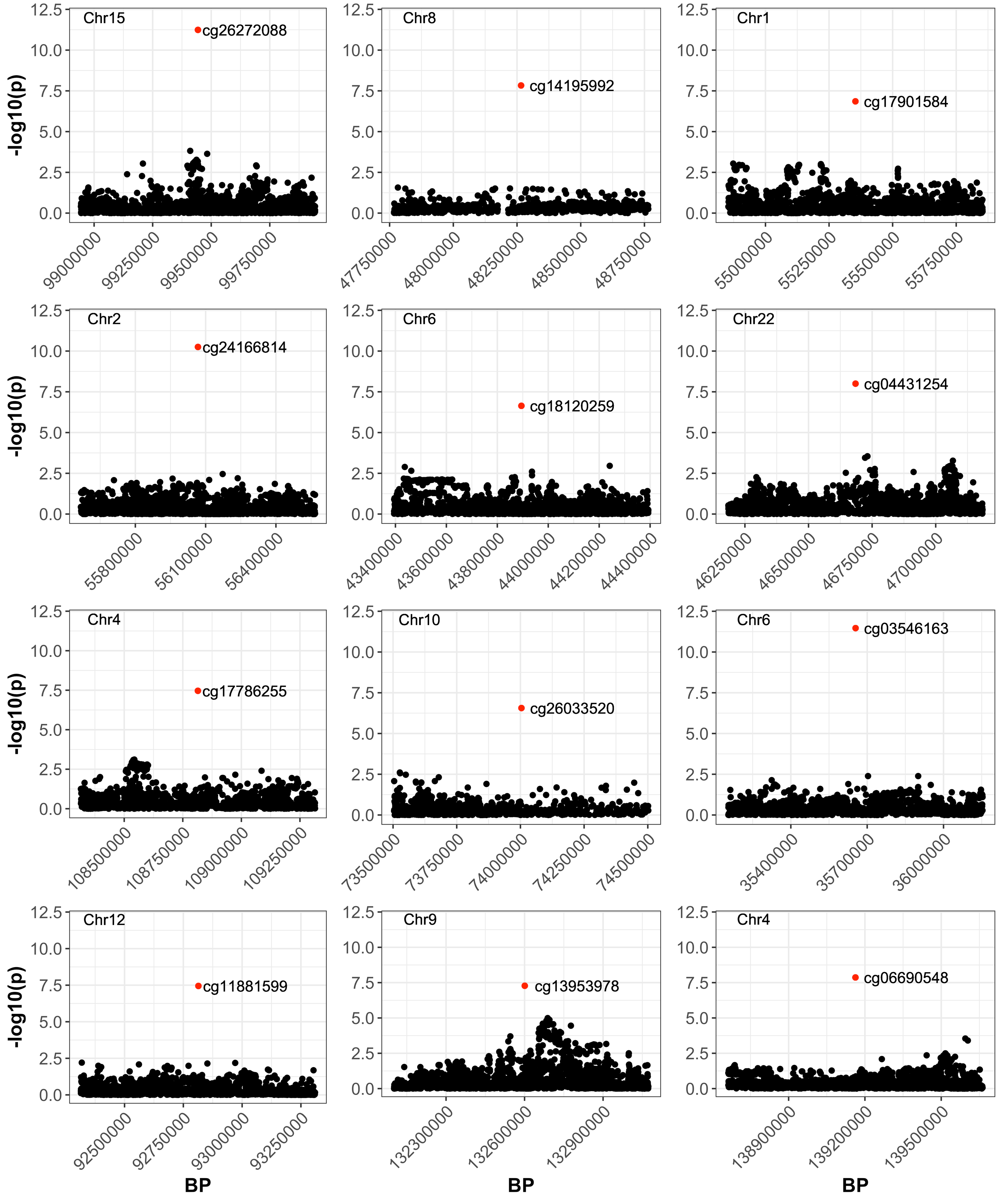


**Fig. S11** - Overlap of the ALS GWAS p-values from Nicolas et al. 2018 [4] (black dots) and p-values from differentially methylated positions from the DNA methylation meta-analysis of AD, PD and ALS (red dots). Loci shown are ± 500kb from each CpG.


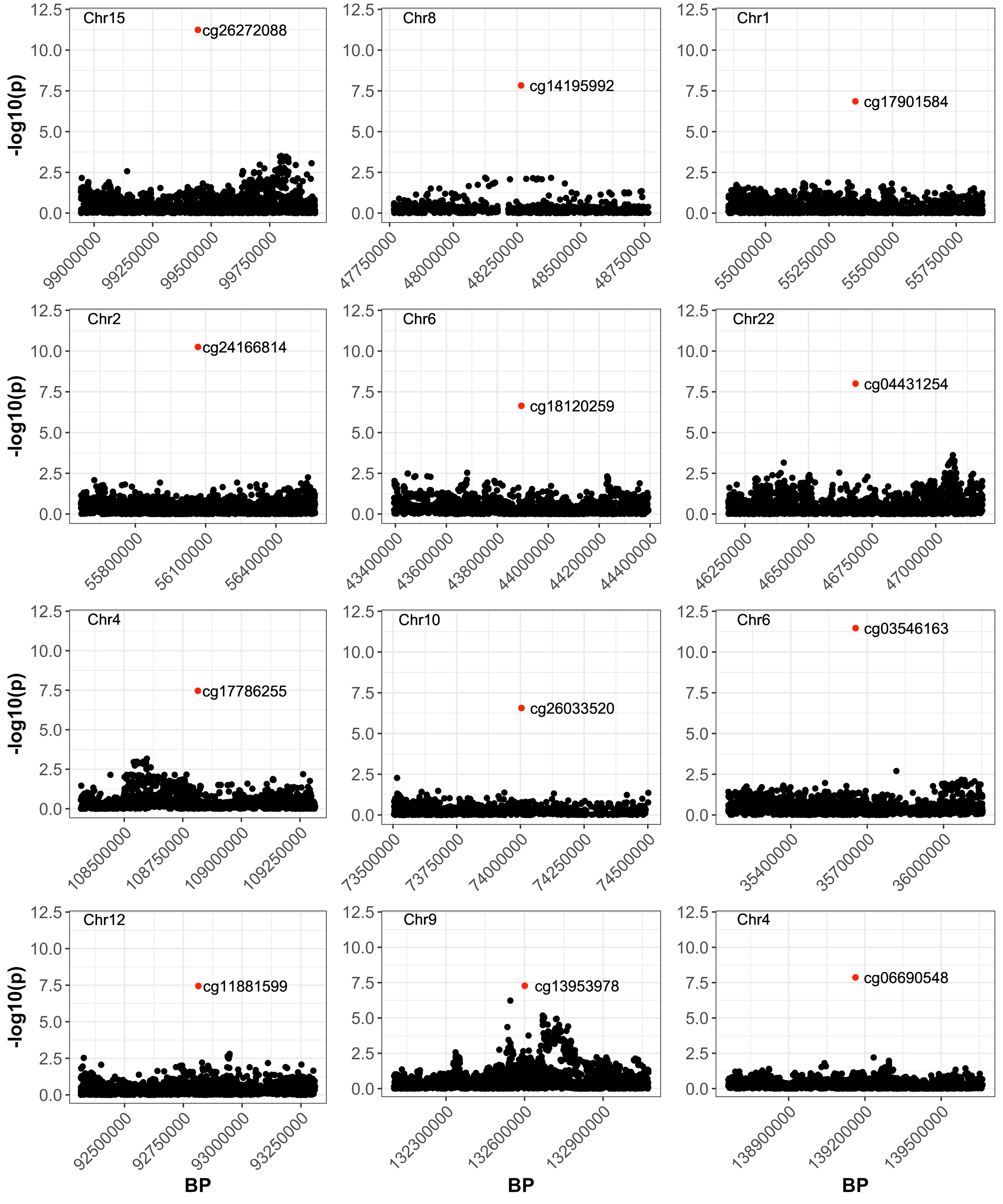


**Fig. S12** - Overlap of GWAS p-values from meta-analysis of Marioni et al. 2019, Nalls et al. 2019 and Nicolas et al. 2018 (black dots) and p-values from differentially methylated positions from the DNA methylation meta-analysis of AD, PD and ALS (red dots). Loci shown are ± 500kb from each CpG.


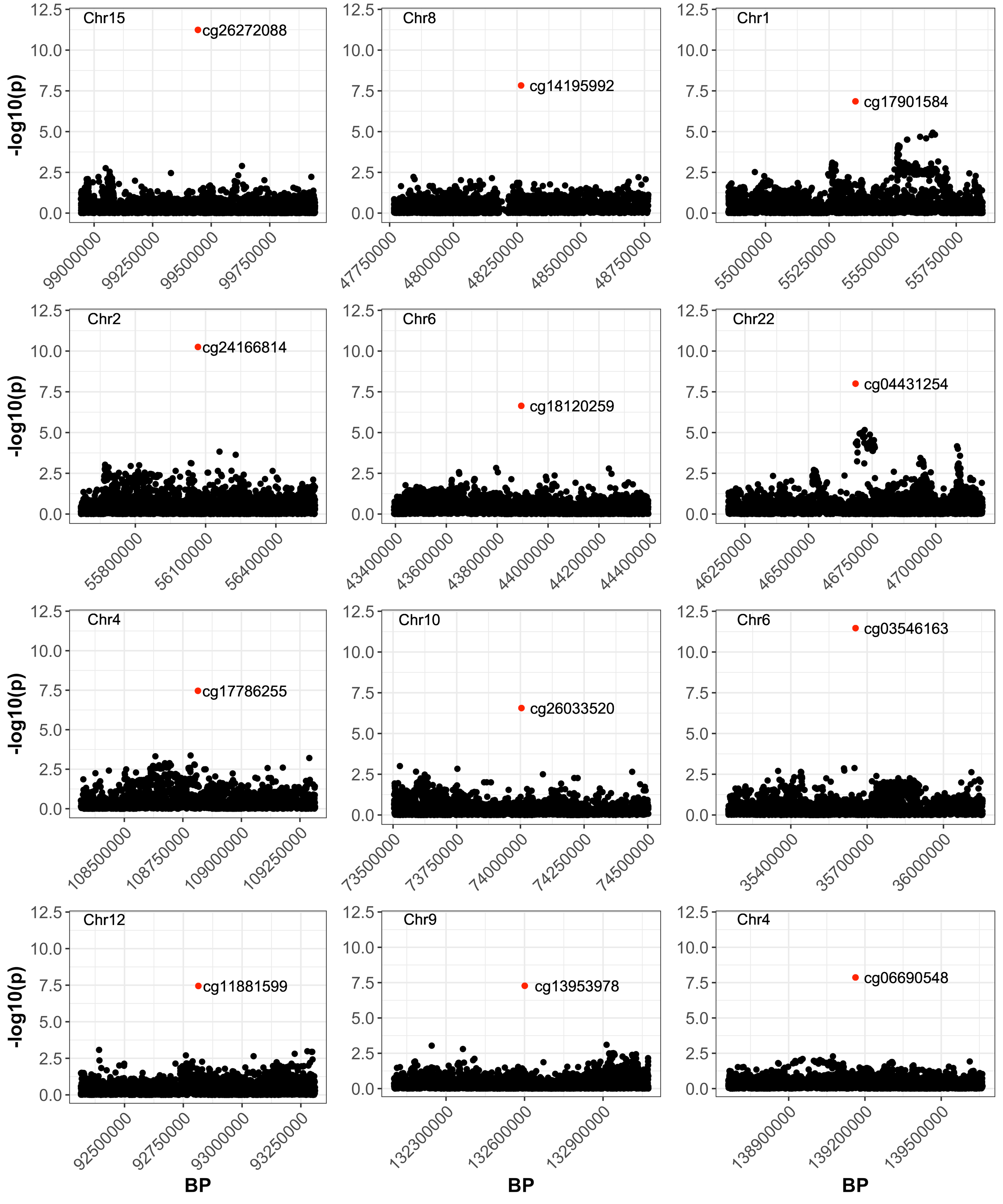


**Fig. S13** - Overlap of the AD GWAS p-values from Kunkle et al. 2019 [6] (black dots) and p-values from differentially methylated positions from the DNA methylation meta-analysis of AD, PD and ALS (red dots). Loci shown are ± 500kb from each CpG.

**
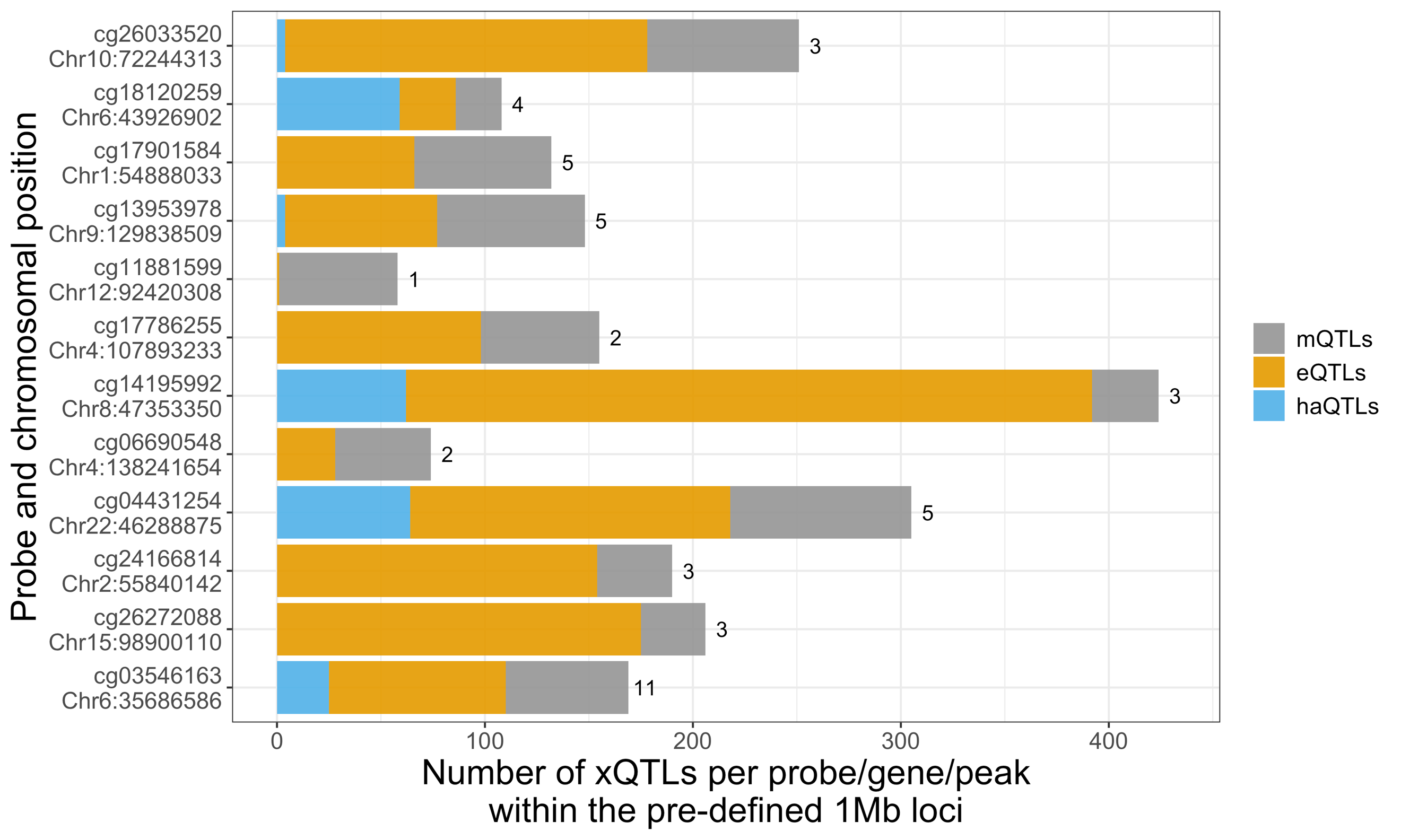
**

**Fig. S14** - Overlap with molecular quantitative trait loci (xQTLs), from AMP-AD consortium [7, 8], at pre-defined loci ±500kb of each of the 12 differentially methylated positions, from the MOMENT meta-analysis of AD, ALS and PD. mQTLs - methylation quantitative trait loci (gray); eQTLs - gene expression quantitative trait loci (orange); haQTLs - histone acetylation quantitative loci (blue). The numbers on top of the bars refer to the number of unique genes within that region.

# Out-of-sample classification accuracy between disorders from DNA methylation-derived profile scores (MPS)


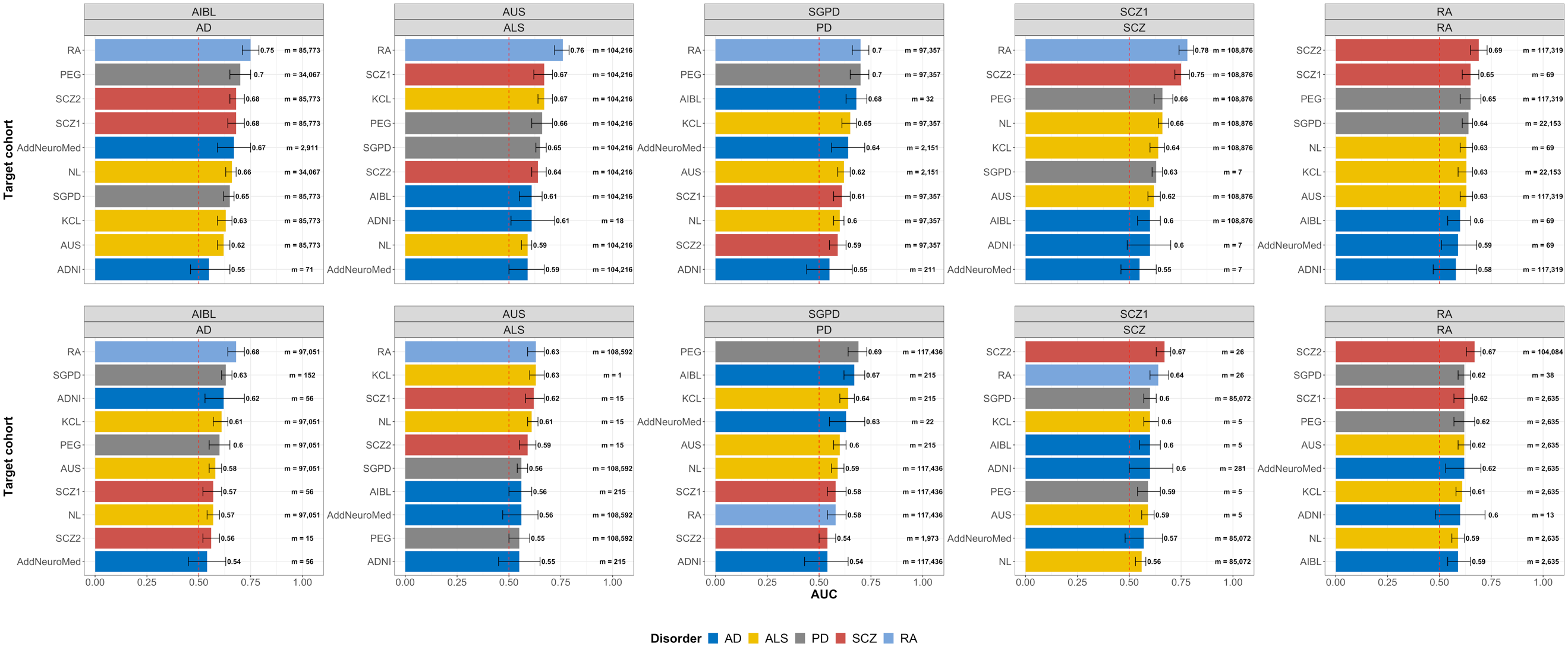


**Fig. S15 -** Maximum accuracy of pairwise out-of-sample classification, within and between disorders, using DNAm-derived profile sum scores (MPS). MPS were calculated keeping effect sizes that passed different p-value thresholds, in each MOA (top-row) or MOMENT (bottom-row) MWAS, of each discovery cohort. Effect sizes of each DNAm site were then multiplied by each DNAm site value in the target cohorts, summed over all sites. Classification accuracy of the MPS was evaluated by the area under the receiver-operator characteristic (ROC) curves (AUC); m = number of probes in the classifier; p = p-values from logistic regression models used to assess AUC.

# Analysis of DNAm-derived immune cell-type proportions


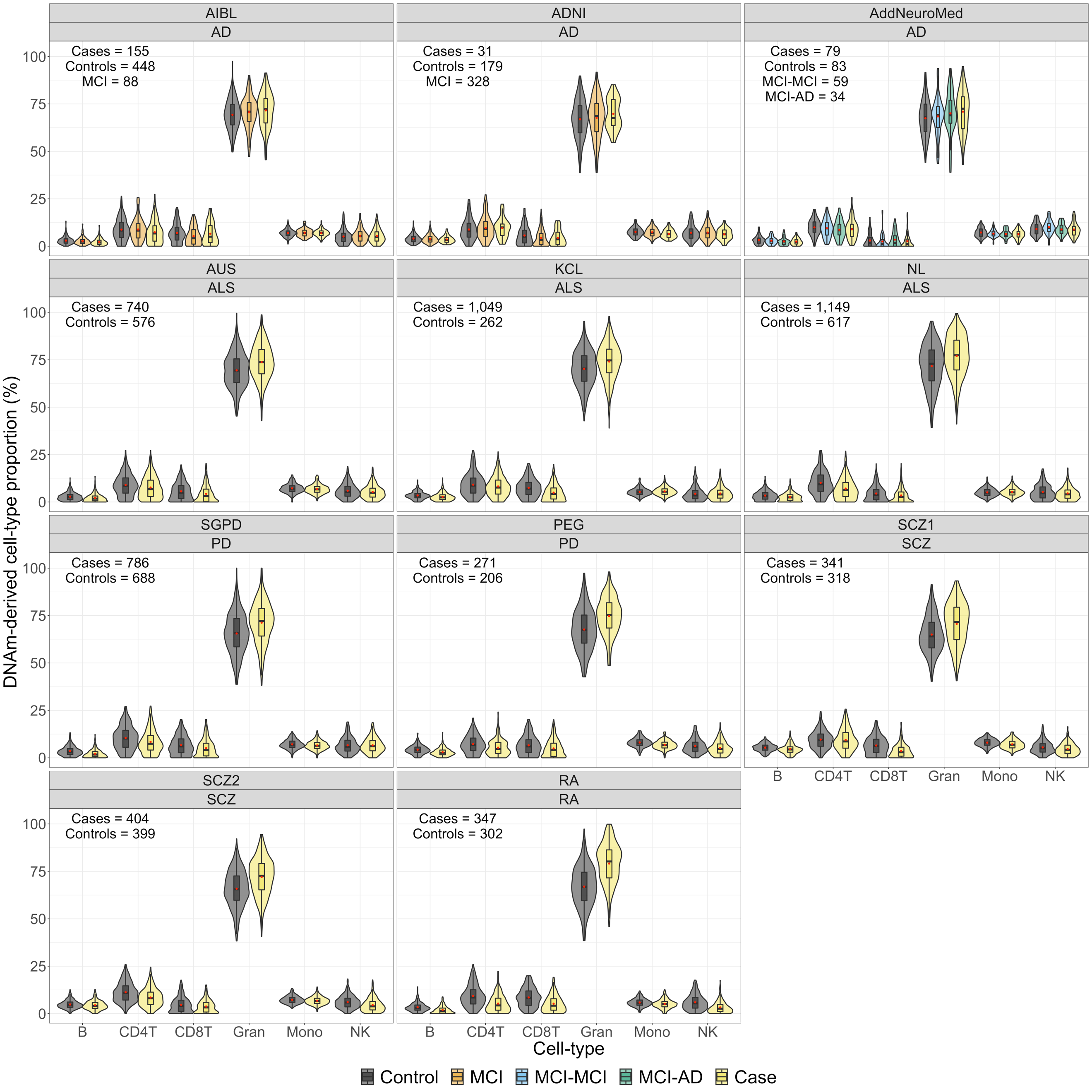


**Fig. S16 - Violinplots of predicted cell-type proportions (CTP) in cases and controls of each cohort.** ALS - amyotrophic lateral sclerosis, AD - Alzheimer’s disease, MCI - Mild cognitive impairment, PD - Parkinson’s disease, RA - Rheumatoid arthritis and SCZ - schizophrenia. The boxplot horizontal black line marks the median CTP value in that group. The red circle inside the boxplots marks the mean CTP value in that group. The lower and upper hinges correspond to the first and third quartiles (the 25th and 75th percentiles). The upper whisker extends from the hinge to the largest value no further than 1.5 * IQR from the hinge (where IQR is the inter-quartile range, or distance between the first and third quartiles). The lower whisker extends from the hinge to the smallest value at most 1.5 * IQR of the hinge.


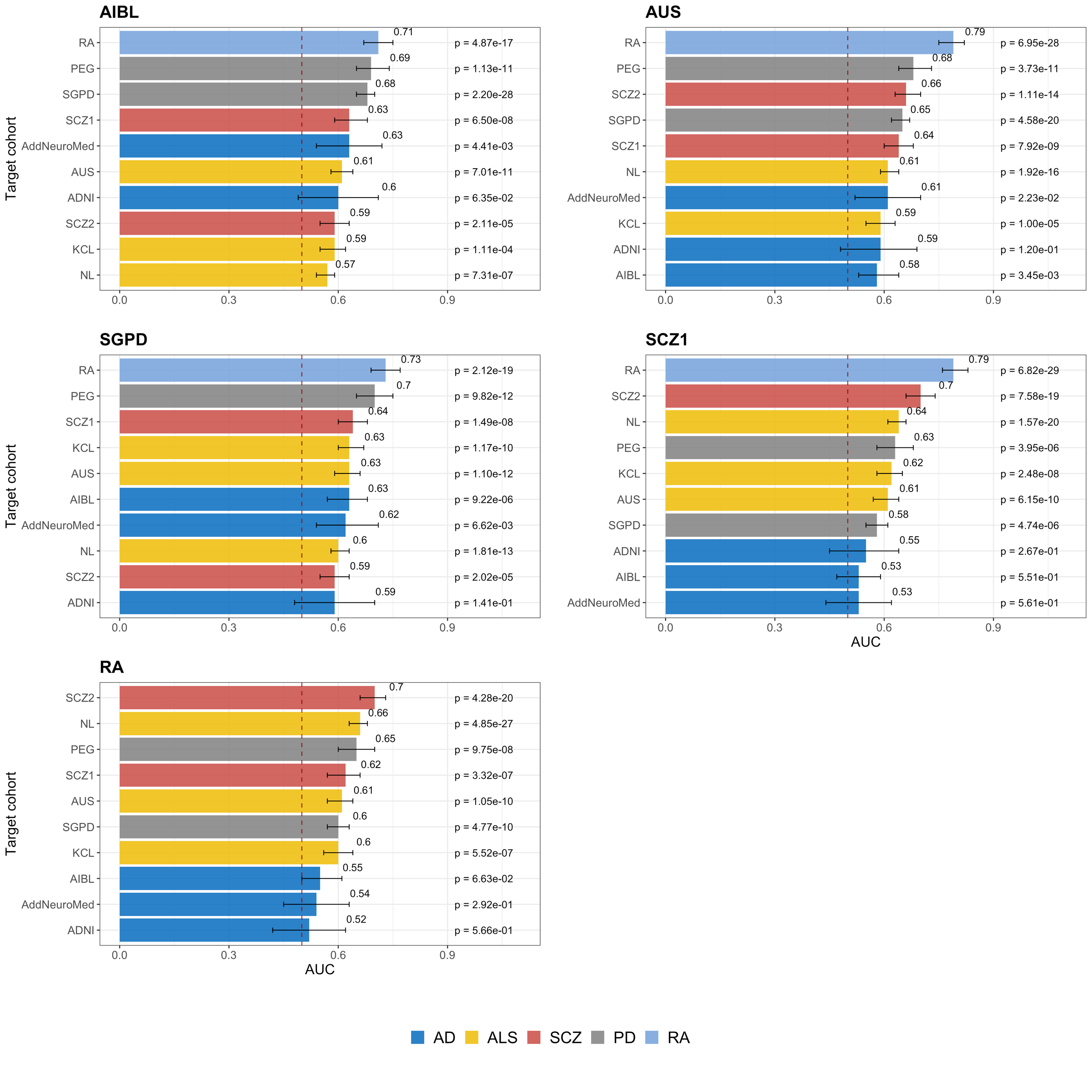


**Fig. S17** - Accuracy of pairwise out-of-sample classification, within and between disorders, using cell-type proportion (CTP) sum-scores. CTP effect sizes were estimated from logistic regression models for each discovery cohort, that included case-control status as response variable and CTP (B cells, CD4+ T cells, monocytes, natural killer cells and granulocytes), sex, predicted age and smoking scores as covariates. Effect sizes of each CTP were then multiplied by each CTP in the target cohorts, summed over all CTP. Classification accuracy of the CTP-scores was evaluated by the area under the receiver-operator characteristic (ROC) curves (AUC); p = p-values from logistic regression models used to assess AUC.


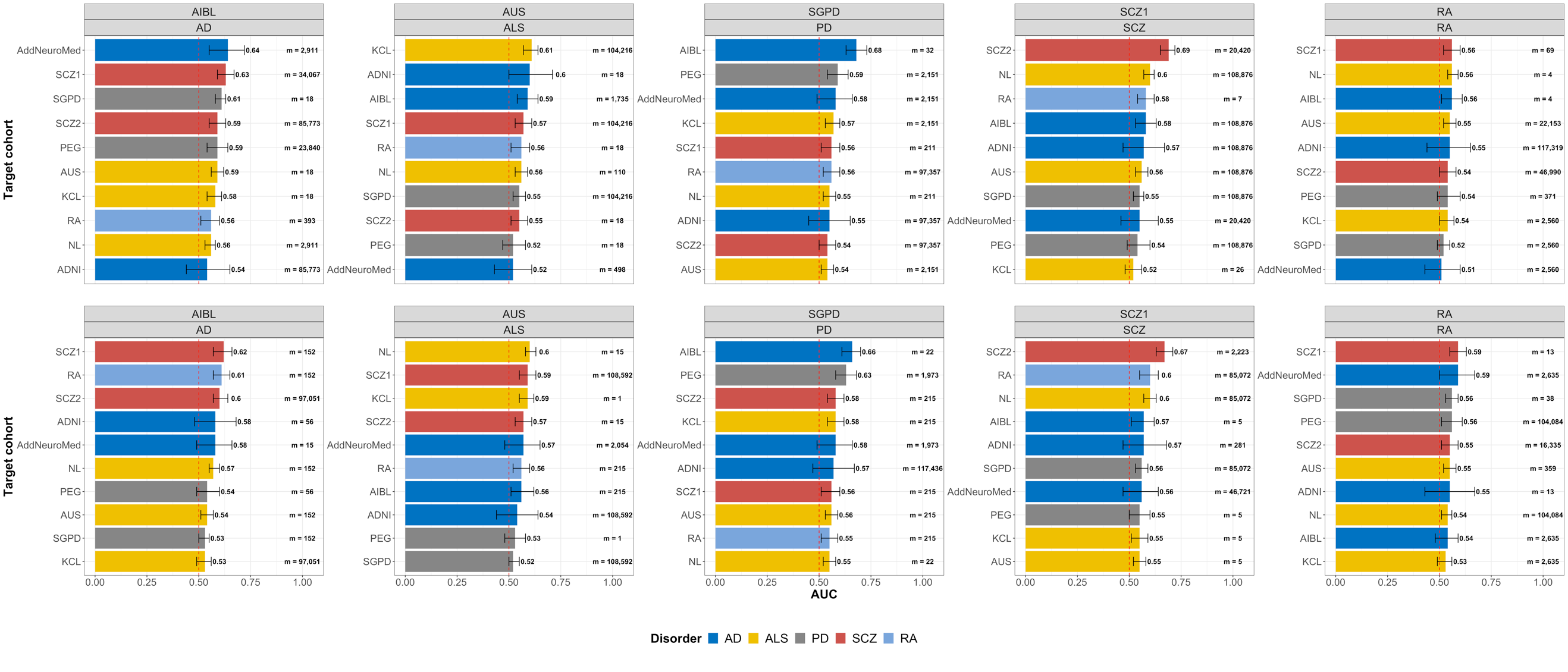


**Fig. S18** - Maximum accuracy of pairwise out-of-sample classification, within and between disorders, using DNAm-derived profile sum scores (MPS), adjusted by cell-type proportions (B cells, CD4+ T lymphocytes, CD8+T lymphocytes, natural killer cells, monocytes and neutrophils). MPS were calculated keeping effect sizes that passed different p-value thresholds, in each MOA (top-row) or MOMENT (bottom-row) MWAS, of each discovery cohort. Effect sizes of each DNAm site were then multiplied by each DNAm site value in the target cohorts, summed over all sites. Classification accuracy of the MPS was evaluated by the area under the receiver-operator characteristic (ROC) curves (AUC); m = number of probes in the classifier; p = p-values from logistic regression models used to assess AUC.

# Significant correlations of CTP profile scores with blood protein markers of inflammation in the Lothian Birth Cohort 1936 (LBC36), but not with MOMENT profile scores

**
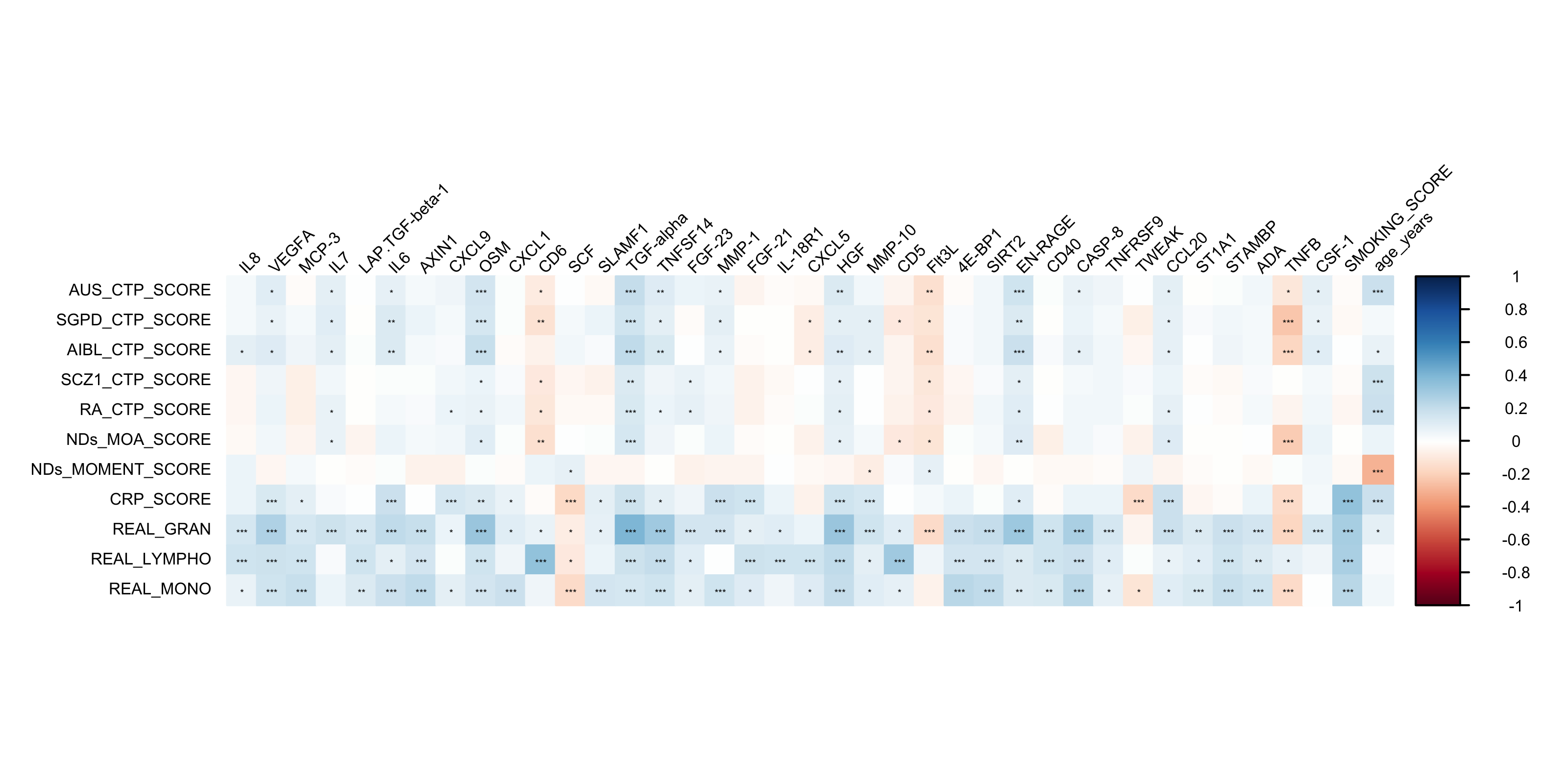
**

**Fig. S19 - Correlogram of blood protein inflammation markers (columns) and disease-associated profile scores (rows) in the Lothian Birth Cohort 1936 (N = 823).** First five rows show correlations with disease-associated cell-type proportion (CTP) scores; next two rows show DNAm profile scores (MPS) derived from MOA and MOMENT meta-analyses of neurodegenerative disorders; CRP-score refers to scores derived from a C-reactive protein MWAS associated with low-grade chronic inflammation and last three rows refer to correlations with real granulocytes, lymphocytes and monocytes, respectively. AUS - amyotrophic lateral sclerosis discovery cohort; SGPD - Parkinson’s disease discovery cohort; AIBL - Alzheimer’s disease discovery cohort; SCZ1 - schizophrenia discovery cohort; RA - rheumatoid arthritis discovery cohort; NDs_MOA_SCORE - MPS from MOA meta-analysis of AIBL, AUS, KCL, NL, SGPD and PEG; NDs_MOMENT_SCORE - MPS from MOMENT meta-analysis of AIBL, AUS, KCL, NL, SGPD and PEG. *** - p-value < 1x10^-4^, ** - p-value < 1x10^-3^, * - p-value < 0.05. For visualization purposes we only show markers with at least one correlation p-value < 1x10^-4^.


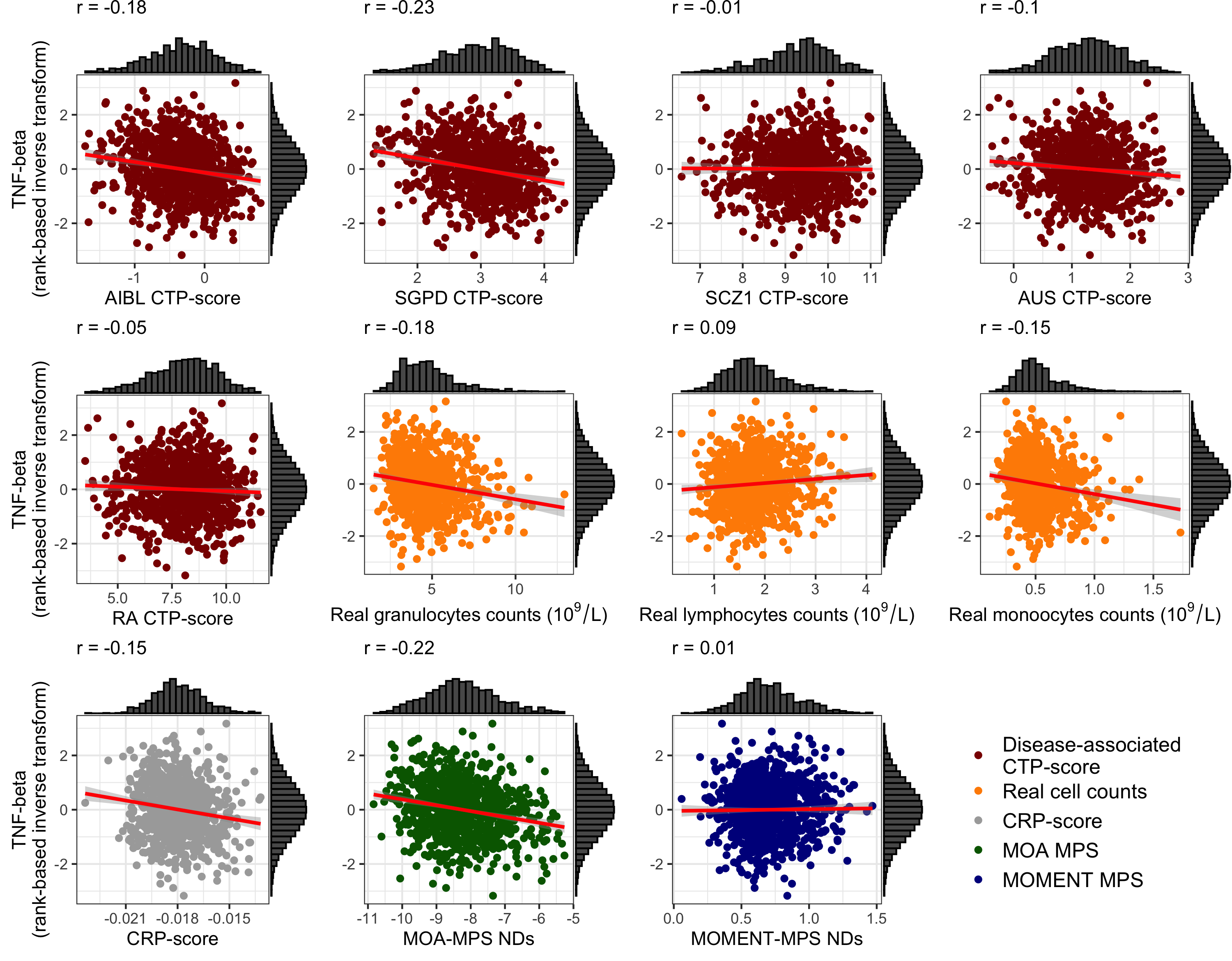


**Fig. S20 - Marginal plots of TNF-beta (rank-based inverse transform) vs disease-associated CTP-scores (dark red), real white blood cell counts (10^9^/L, in orange), DNAm-derived CRP-scores (gray), MOA- (dark green) and MOMENT-MPS (dark blue), in the Lothian Birth Cohort 1936 (N = 823).** The red line shows the best linear fit to the data, with gray background representing the s.e. AUS - amyotrophic lateral sclerosis discovery cohort; SGPD - Parkinson’s disease discovery cohort; AIBL - Alzheimer’s disease discovery cohort; SCZ1 - schizophrenia discovery cohort; RA - rheumatoid arthritis discovery cohort; NDs_MOA_SCORE - MPS from MOA meta-analysis of AIBL, AUS, KCL, NL, SGPD and PEG; NDs_MOMENT_SCORE - MPS from MOMENT meta-analysis of AIBL, AUS, KCL, NL, SGPD and PEG. The red line shows the best linear fit to the data, with gray background representing the s.e.


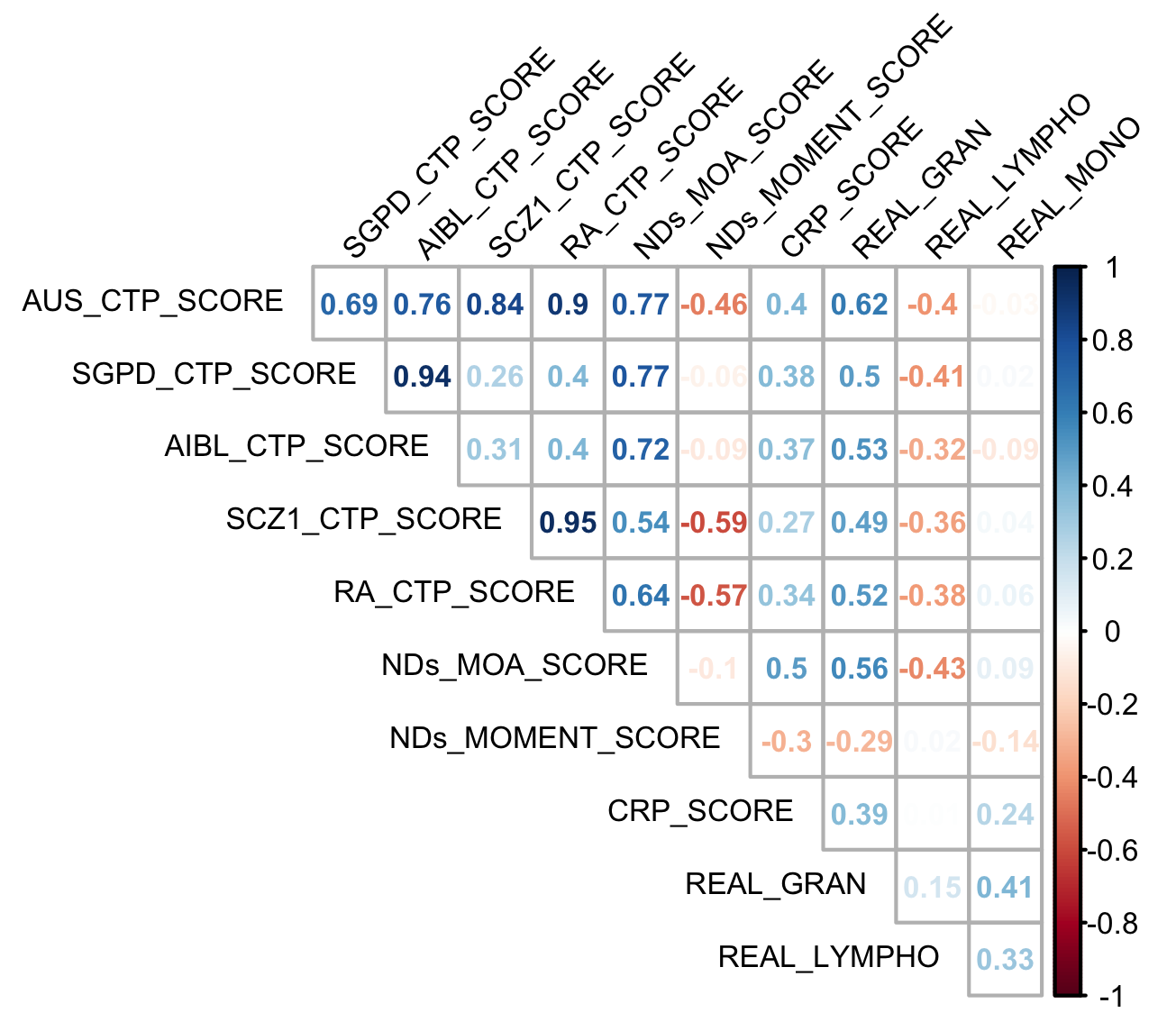


**Fig. S21 - Pearson correlations between disease-associated cell-type proportion scores, blood white cell counts and MOA- and MOMENT DNA methylation derived profile scores (effect sizes estimates from MOA or MOMENT meta-analyses of AD, ALS and PD), in the Lothian Birth Cohort 1936 (N = 820).** AIBL - Alzheimer’s disease discovery cohort; SCZ1 - schizophrenia discovery cohort; RA - rheumatoid arthritis discovery cohort; NDs_MOA_SCORE - MPS from MOA meta-analysis of AIBL, AUS, KCL, NL, SGPD and PEG; NDs_MOMENT_SCORE - MPS from MOMENT meta-analysis of AIBL, AUS, KCL, NL, SGPD and PEG.


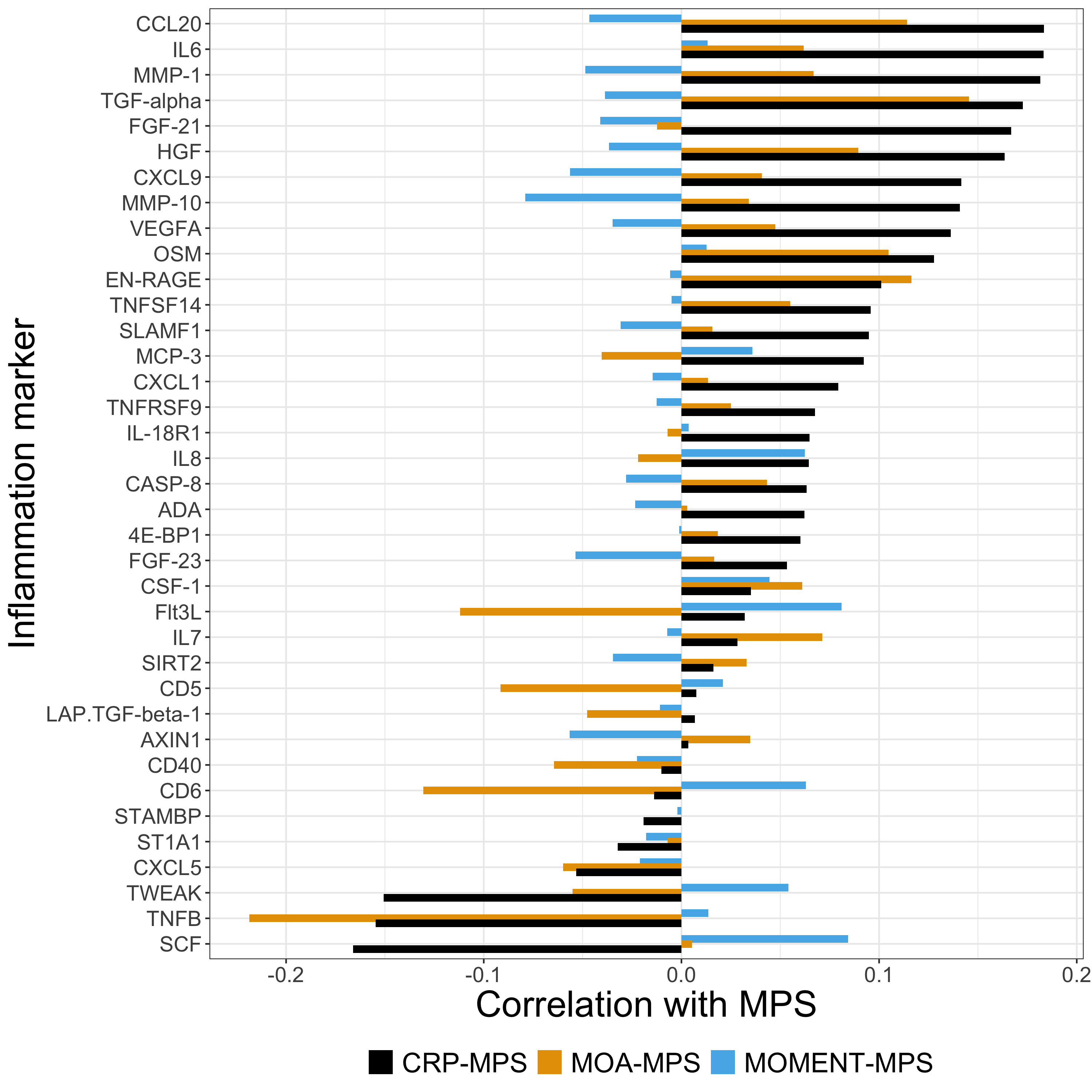


**Fig. S22 - Correlation of blood protein inflammatory markers, CRP-MPS and MOA- and MOMENT-MPS, in the Lothian Birth Cohort 1936 (N = 823).** For visualization purposes we only show markers with at least one correlation p-value < 1x10^-4^, as in Fig. S17.


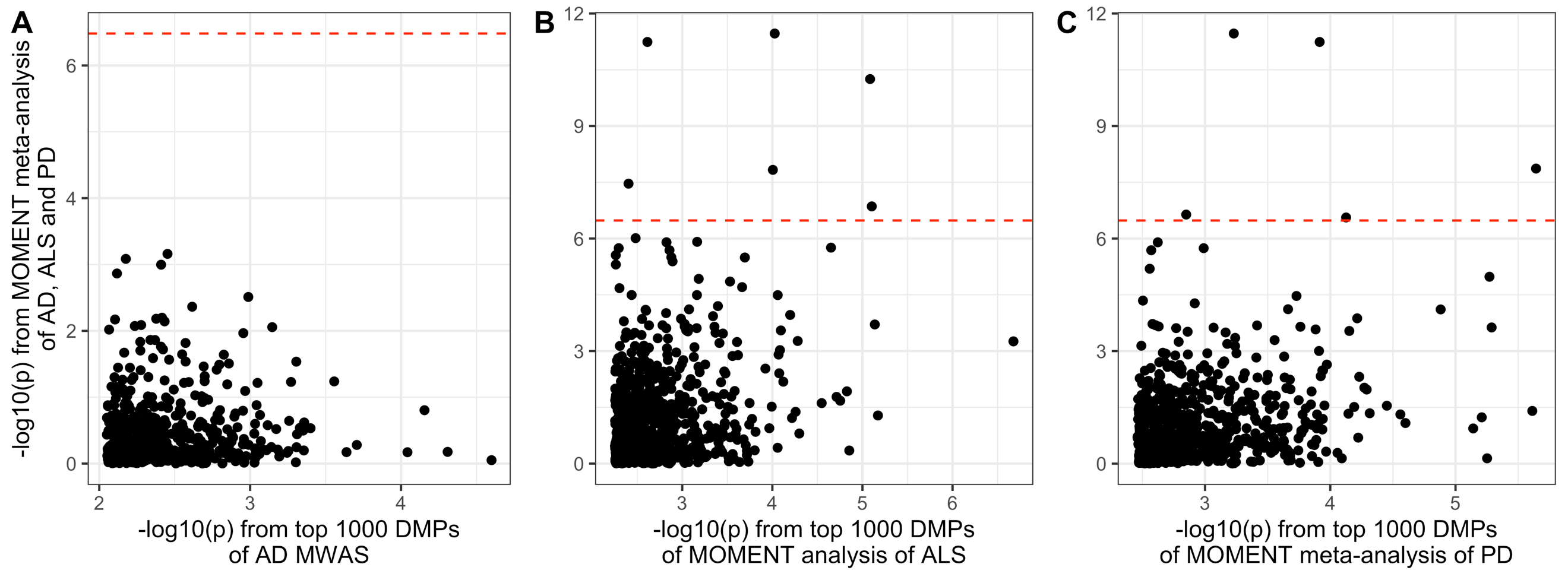


**Fig. S23** - No evidence for overlap of results from our MOMENT meta-analysis of blood DNA methylation data from AD, ALS and PD with previous results from blood MWAS of the individual phenotypes. **A)** -log10(p-values) of MOMENT meta-analysis of AD, PD and ALS (y-axis) vs -log10(p-values) from the Tukey's HSD test of control and Alzheimer's disease blood samples top 1000 DMPs (x-axis) [9], **B)** -log10(p-values) of MOMENT meta-analysis of AD, PD and ALS (y-axis) vs -log10(p-values) from top 1000 DMPs of MOMENT MWAS of ALS (x-axis) [10] and **C)** -log10(p-values) of MOMENT meta-analysis of AD, PD and ALS (y-axis) vs -log10(p-values) from top 1000 DMPs of MOMENT meta-analysis of PD (x-axis) [11]. Red dotted line indicates the Bonferroni corrected p-value from the MOMENT meta-analysis of AD, ALS and PD. Only 482 of the top 1000 DMPs in the previous AD study were present in our meta-analysis. 694 and 809 of the top 1000 DMPs from PD and ALS studies, respectively, were present in our meta-analysis. 5 and 6 DMPs from our meta-analysis overlapped with the top 1000 DMPs from the PD and ALS studies, respectively. No DMP from our meta-analysis overlapped with the top 1000 DMPs from the AD study.


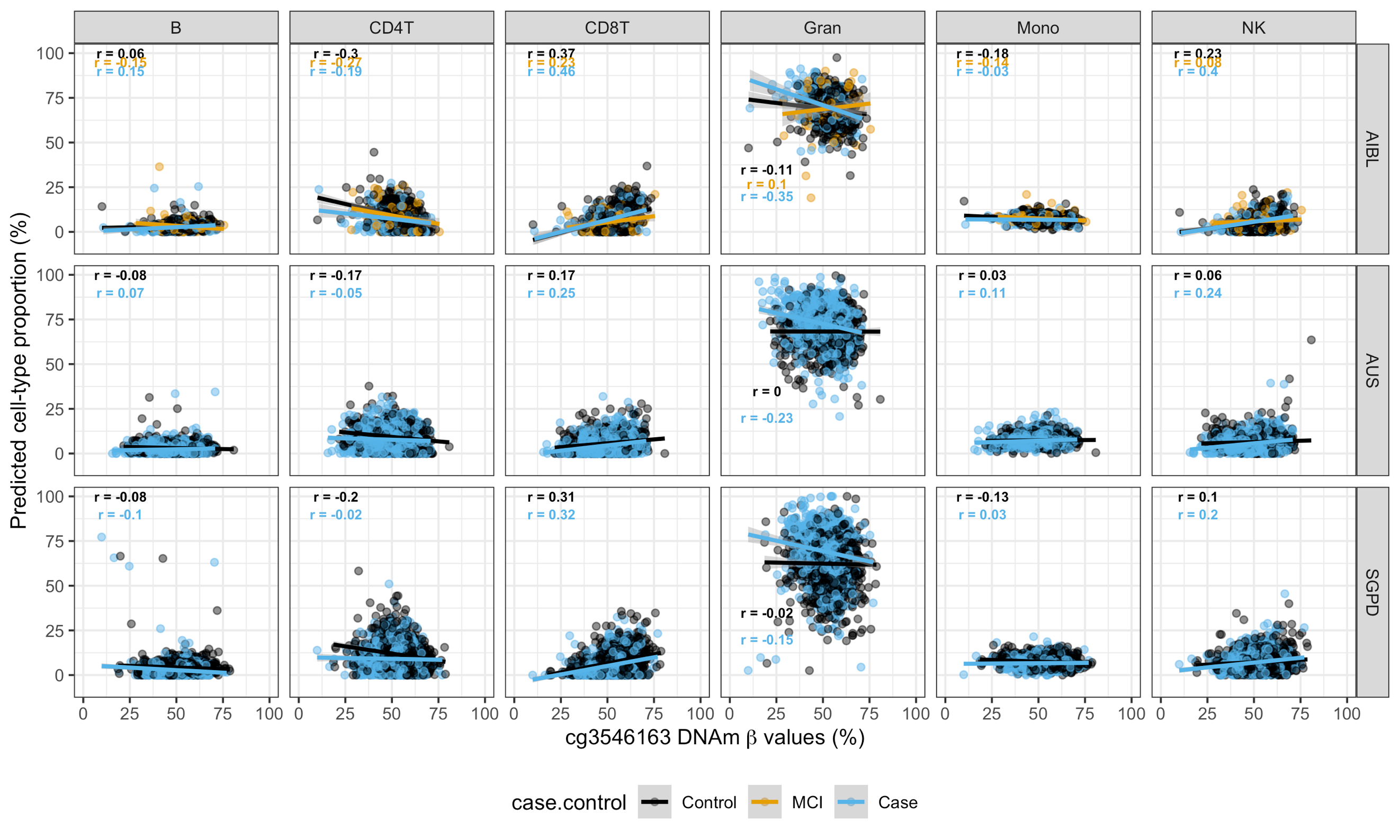


**Fig. S24** - DNA methylation β values (x-axis) of probe cg3546163, annotated to gene FKBP5 vs cell-type proportions (columns) in the discovery cohorts used for MOMENT meta-analyses of neurodegenerative disorders (rows). AIBL - Alzheimer’s disease cohort; AUS - amyotrophic lateral sclerosis cohort; SGPD - Parkinson’s disease cohort. Solid lines represent best linear fit to the data within case-control status.


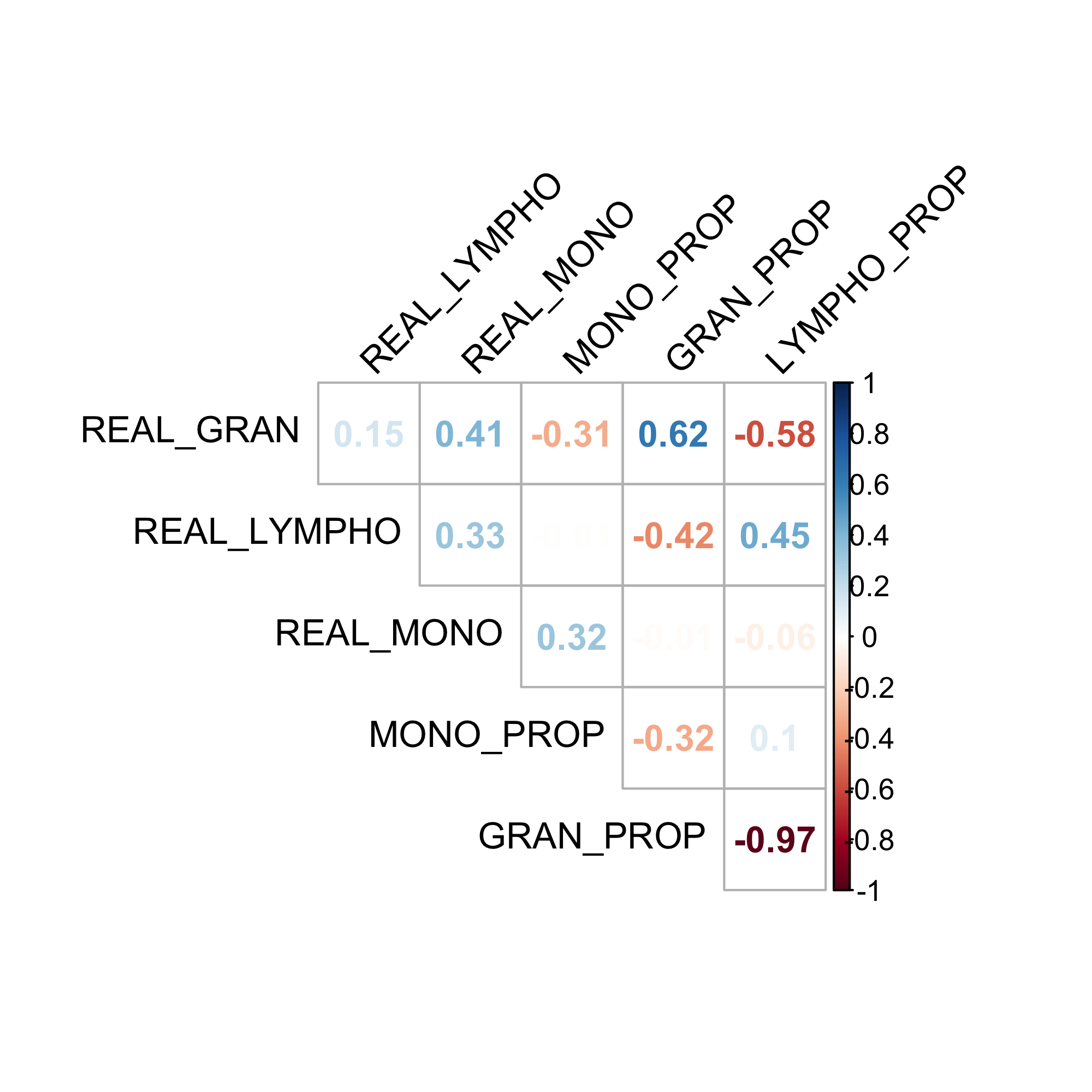


**Fig. S25 - Pearson correlations between real blood cell counts (10^9^/L) and DNAm-derived cell-type proportion, in the Lothian Birth Cohort 1936 (N = 823).** REAL_LYMPHO - real lymphocyte counts, REAL_GRAN - real granulocyte counts, REAL_MONO - real monocyte counts, GRAN_PROP - estimated granulocyte proportion, MONO_PROP - estimated monocyte proportion, LYMPHO_PROP - estimated lymphocyte proportion.

# Supplementary Note - Literature evidence for functional role of 12 DMPs in MOMENT meta-analysis of neurodegenerative disorders

FKBP51, encoded by *FKBP5* is peptidyl-prolyl-cis/trans isomerase, with co-chaperone activities that affect folding and activity of other proteins. Among many other functions, FKBP51 has been shown to modulate glucocorticoid receptor (GR) binding, transcriptional activity and cellular trafficking [12, 13]. In response to stressors, glucocorticoids act as downstream effectors of the hypothalamic-pituitary-adrenal axis and regulate physiological mechanisms, such as increased cardiovascular tone, respiratory rate, and intermediate metabolism, along with inhibition of general vegetative functions such as feeding, digestion, growth, reproduction, and immunity [14, 15]. Importantly, GRs and FKBP51 are also involved in negative feedback loops following stress, with FKBP51 transcription and translation being rapidly induced by GR activation in multiple tissues, with subsequent inhibition of GR-responsiveness [16]. For this reason, *FKBP5* genetic variation and dysregulation have been hypothesized to be implicated in the pathogenesis of stress-related, mood and anxiety disorders [16, 17]. In particular, candidate gene assays of *FKBP5* DNA demethylation have been extensively used in the context of childhood abuse, with data supporting a developmentally restricted effect of early trauma and glucocorticoid exposure on selective *FKBP5* methylation sites that appears to occur across peripheral and neuronal tissues and to depend on susceptible *FKBP5* variants [16, 18].

The second most-associated probe cg26272088 (**Table 1**, p_META_ = 5.74x10^-12^, increased blood DNAm in cases compared to controls), annotated to *IGF1-R*, a receptor for the insulin-growth factor 1 (IGF-1). Although essential for CNS development, the role of IGF-1 in the aging brain remains controversial and opposite concepts can be found in the literature, likely due to highly pleiotropic interactions [19]. These opposing effects can also be found in the periphery, with IGF-1 pro- and anti-inflammatory actions being reported in animal models of disease. In the context of the anti-inflammatory effects of IGF-1, it has been suggested that development of resistance to IGF-1 (for example, impaired IGF-1R signaling) may contribute to neuroinflammation and progression of major brain diseases. In the context of ALS, animal studies have shown that hSOD1^G93A^ mice at “end stage of disease” had normal levels of muscle IGF-I protein expression, but decreased circulating levels of IGF-I and skeletal muscle IGF-IRα protein expression [10, 20, 21]. More importantly, these changes were variable according to disease progression. Interestingly, IGF-I-directed interventions prolong survival in a mouse model of ALS [22-24], whereas Growth-Hormone/IGF-I therapies were of no benefit in slowing disease progression in human ALS patients [25-27]. These seemingly inconsistent results illustrate the importance of acknowledging species-specific differences in disease progression and disease stage-specific or tissue-specific interventions, that may account for improved outcome in mouse models of disease.

The associations of probes cg06690548, annotated to gene *SLC7A11* (**Table 1,** p_META_ = 1.36x10^-9^, increased blood DNAm in cases compared to controls) and cg26033520 (**Table 1,** p_META_ = 2.74x10^-7^, increased blood DNAm in cases compared to controls) had been previously reported using MOA, in a meta-analysis of the same PD cohorts [11]. Despite being significantly associated with NDs with the same direction of effects, these probes also showed significant heterogeneity between studies (**Table 1,** *I^2^* = 60.2%, for cg06690548 and *I^2^* = 81.1% for cg26033520). As for the PD probes, cg17901584 (p_META_ = 1.39x10^-7^, increased blood DNAm in cases compared to controls), annotated to *DHCR24* showed significant heterogeneity between studies (*I^2^* = 73.2%), with different direction of effect in the AIBL cohort (i.e., decreased blood DNAm in cases compared to controls). *DHCR24* encodes a flavin adenine dinucleotide (FAD)-dependent oxidoreductase which catalyzes the reduction of the delta-24 double bond of sterol intermediates during cholesterol biosynthesis. It was initially identified as being expressed at lower levels in the affected as compared to unaffected brain regions in AD patients and has proven protective in different AD-related stress conditions, including Aβ-induced, oxidative and neuroinflammatory stress [28, 29].

Another interesting association was the one for probe cg13953978, annotated to *USP20* (p_META_ = 5.28x10^-8^, increased blood DNAm in cases compared to controls), a well-studied deubiquitinase (DUB). DUBs have key roles in various areas of cell biology of high relevance to pathologies such as autoimmune disorders, chronic inflammation, oncology and neurodegeneration. *USP20*‐mediated deubiquitylation of HIF1α prevents proteasomal degradation, allowing for transcription of hypoxic response genes [30]. Thus, inhibition of *USP20* has potential for suppressing proliferation of hypoxic tumor cells.

1. Zhang Q, Vallerga CL, Walker RM, Lin T, Henders AK, Montgomery GW, He J, Fan D, Fowdar J, Kennedy M, et al: **Improved precision of epigenetic clock estimates across tissues and its implication for biological ageing.** *Genome Medicine* 2019, **11:**54.

2. McCartney DL, Hillary RF, Stevenson AJ, Ritchie SJ, Walker RM, Zhang Q, Morris SW, Bermingham ML, Campbell A, Murray AD, et al: **Epigenetic prediction of complex traits and death.** *Genome Biology* 2018, **19:**136.

3. Marioni RE, Harris SE, Zhang Q, McRae AF, Hagenaars SP, Hill WD, Davies G, Ritchie CW, Gale CR, Starr JM, et al: **GWAS on family history of Alzheimer’s disease.** *Translational Psychiatry* 2018, **8:**99.

4. Nicolas A, Kenna KP, Renton AE, Ticozzi N, Faghri F, Chia R, Dominov JA, Kenna BJ, Nalls MA, Keagle P, et al: **Genome-wide Analyses Identify KIF5A as a Novel ALS Gene.** *Neuron* 2018, **97:**1268-1283.e1266.

5. Nalls MA, Blauwendraat C, Vallerga CL, Heilbron K, Bandres-Ciga S, Chang D, Tan M, Kia DA, Noyce AJ, Xue A, et al: **Identification of novel risk loci, causal insights, and heritable risk for Parkinson's disease: a meta-analysis of genome-wide association studies.** *The Lancet Neurology* 2019, **18:**1091-1102.

6. Kunkle BW, Grenier-Boley B, Sims R, Bis JC, Damotte V, Naj AC, Boland A, Vronskaya M, van der Lee SJ, Amlie-Wolf A, et al: **Genetic meta-analysis of diagnosed Alzheimer’s disease identifies new risk loci and implicates Aβ, tau, immunity and lipid processing.** *Nature Genetics* 2019, **51:**414-430.

7. Ng B, White CC, Klein H-U, Sieberts SK, McCabe C, Patrick E, Xu J, Yu L, Gaiteri C, Bennett DA, et al: **An xQTL map integrates the genetic architecture of the human brain's transcriptome and epigenome.** *Nature neuroscience* 2017, **20:**1418-1426.

8. Sieberts SK, Perumal T, Carrasquillo MM, Allen M, Reddy JS, Hoffman GE, Dang KK, Calley J, Ebert PJ, Eddy J, et al: **Large eQTL meta-analysis reveals differing patterns between cerebral cortical and cerebellar brain regions.** *bioRxiv* 2019**:**638544.

9. Roubroeks JAY, Smith AR, Smith RG, Pishva E, Ibrahim Z, Sattlecker M, Hannon EJ, Kłoszewska I, Mecocci P, Soininen H, et al: **An epigenome-wide association study of Alzheimer's disease blood highlights robust DNA hypermethylation in the HOXB6 gene.** *Neurobiology of Aging* 2020, **95:**26-45.

10. Nabais MF, Lin T, Benyamin B, Williams KL, Garton FC, Vinkhuyzen AAE, Zhang F, Vallerga CL, Restuadi R, Freydenzon A, et al: **Significant out-of-sample classification from methylation profile scoring for amyotrophic lateral sclerosis.** *npj Genomic Medicine* 2020, **5:**10.

11. Vallerga CL, Zhang F, Fowdar J, McRae AF, Qi T, Nabais MF, Zhang Q, Kassam I, Henders AK, Wallace L, et al: **Analysis of DNA methylation associates the cystine–glutamate antiporter SLC7A11 with risk of Parkinson’s disease.** *Nature Communications* 2020, **11:**1238.

12. Davies TH, Ning Y-M, Sánchez ER: **A New First Step in Activation of Steroid Receptors: HORMONE-INDUCED SWITCHING OF FKBP51 AND FKBP52 IMMUNOPHILINS.** *Journal of Biological Chemistry* 2002, **277:**4597-4600.

13. Wochnik GM, Rüegg J, Abel GA, Schmidt U, Holsboer F, Rein T: **FK506-binding Proteins 51 and 52 Differentially Regulate Dynein Interaction and Nuclear Translocation of the Glucocorticoid Receptor in Mammalian Cells.** *Journal of Biological Chemistry* 2005, **280:**4609-4616.

14. Silverman MN, Sternberg EM: **Glucocorticoid regulation of inflammation and its functional correlates: from HPA axis to glucocorticoid receptor dysfunction.** *Annals of the New York Academy of Sciences* 2012, **1261:**55-63.

15. Smith SM, Vale WW: **The role of the hypothalamic-pituitary-adrenal axis in neuroendocrine responses to stress.** *Dialogues in clinical neuroscience* 2006, **8:**383-395.

16. Zannas AS, Wiechmann T, Gassen NC, Binder EB: **Gene–Stress–Epigenetic Regulation of FKBP5: Clinical and Translational Implications.** *Neuropsychopharmacology* 2016, **41:**261-274.

17. Binder EB, Bradley RG, Liu W, Epstein MP, Deveau TC, Mercer KB, Tang Y, Gillespie CF, Heim CM, Nemeroff CB, et al: **Association of FKBP5 Polymorphisms and Childhood Abuse With Risk of Posttraumatic Stress Disorder Symptoms in Adults.** *JAMA* 2008, **299:**1291-1305.

18. Klengel T, Mehta D, Anacker C, Rex-Haffner M, Pruessner JC, Pariante CM, Pace TWW, Mercer KB, Mayberg HS, Bradley B, et al: **Allele-specific FKBP5 DNA demethylation mediates gene–childhood trauma interactions.** *Nature Neuroscience* 2013, **16:**33-41.

19. Sriram G, Gabriela Farias Q, Nir B, Derek MH, Sofiya M: **40 YEARS of IGF1: IGF1: the Jekyll and Hyde of the aging brain.** *Journal of Molecular Endocrinology* 2018, **61:**T171-T185.

20. Steyn FJ, Lee K, Fogarty MJ, Veldhuis JD, McCombe PA, Bellingham MC, Ngo ST, Chen C: **Growth Hormone Secretion Is Correlated With Neuromuscular Innervation Rather Than Motor Neuron Number in Early-Symptomatic Male Amyotrophic Lateral Sclerosis Mice.** *Endocrinology* 2013, **154:**4695-4706.

21. Steyn FJ, Ngo ST, Lee JD, Leong JW, Buckley AJ, Veldhuis JD, McCombe PA, Chen C, Bellingham MC: **Impairments to the GH-IGF-I Axis in hSOD1G93A Mice Give Insight into Possible Mechanisms of GH Dysregulation in Patients with Amyotrophic Lateral Sclerosis.** *Endocrinology* 2012, **153:**3735-3746.

22. Dobrowolny G, Aucello M, Molinaro M, Musarò A: **Local expression of mIgf-1 modulates ubiquitin, caspase and CDK5 expression in skeletal muscle of an ALS mouse model.** *Neurological Research* 2008, **30:**131-136.

23. Dobrowolny G, Giacinti C, Pelosi L, Nicoletti C, Winn N, Barberi L, Molinaro M, Rosenthal N, Musarò A: **Muscle expression of a local Igf-1 isoform protects motor neurons in an ALS mouse model.** *The Journal of Cell Biology* 2005, **168:**193.

24. Palazzolo I, Stack C, Kong L, Musaro A, Adachi H, Katsuno M, Sobue G, Taylor JP, Sumner CJ, Fischbeck KH, Pennuto M: **Overexpression of IGF-1 in muscle attenuates disease in a mouse model of spinal and bulbar muscular atrophy.** *Neuron* 2009, **63:**316-328.

25. Saccà F, Quarantelli M, Rinaldi C, Tucci T, Piro R, Perrotta G, Carotenuto B, Marsili A, Palma V, De Michele G, et al: **A randomized controlled clinical trial of growth hormone in amyotrophic lateral sclerosis: clinical, neuroimaging, and hormonal results.** *Journal of Neurology* 2012, **259:**132-138.

26. Smith RA, Melmed S, Sherman B, France J, Munsat TL, Festoff BW: **Recombinant growth hormone treatment of amyotrophic lateral sclerosis.** *Muscle & Nerve* 1993, **16:**624-633.

27. Sorenson EJ, Windbank AJ, Mandrekar JN, Bamlet WR, Appel SH, Armon C, Barkhaus PE, Bosch P, Boylan K, David WS, et al: **Subcutaneous IGF-1 is not beneficial in 2-year ALS trial.** *Neurology* 2008, **71:**1770.

28. Greeve I, Hermans-Borgmeyer I, Brellinger C, Kasper D, Gomez-Isla T, Behl C, Levkau B, Nitsch RM: **The Human DIMINUTO/DWARF1 Homolog Seladin-1 Confers Resistance to Alzheimer’s Disease-Associated Neurodegeneration and Oxidative Stress.** *The Journal of Neuroscience* 2000, **20:**7345.

29. Martiskainen H, Paldanius KMA, Natunen T, Takalo M, Marttinen M, Leskelä S, Huber N, Mäkinen P, Bertling E, Dhungana H, et al: **DHCR24 exerts neuroprotection upon inflammation-induced neuronal death.** *Journal of Neuroinflammation* 2017, **14:**215.

30. Harrigan JA, Jacq X, Martin NM, Jackson SP: **Deubiquitylating enzymes and drug discovery: emerging opportunities.** *Nature Reviews Drug Discovery* 2018, **17:**57-78.
