# Supplementary material for: Meta-analysis of genome-wide DNA methylation identifies shared associations across neurodegenerative disorders
Source: Genome Biol. 2021 Mar 26;22:90. doi: 10.1186/s13059-021-02275-5 (PMC8004462; doi:10.1186/s13059-021-02275-5)
Supplement: Supplementary file 3 — Additional file 3. Review history. [file 13059_2021_2275_MOESM3_ESM.docx]

Review History

**First round of review**

**Reviewer 1**

**Are you able to assess all statistics in the manuscript, including the appropriateness of statistical tests used?** Yes, and I have assessed the statistics in my report.

**Comments to author:**

The authors performed an epigenome-wide association using Illumina DNA methylation BeadChips and blood samples to test for CpG sites which DNA methylation level is associated with neurodegenerative disorders.

A subsequent meta-analysis of the ALS, PD and AD results revealed 12 significantly associated CpG sites. In subsequent analyses, it was tested whether these results were driven by inflammatory markers including blood cell type composition. Further characterization of the results included a gene-set enrichment analysis.

The results are very interesting and well described. The applied methods are state-of-the-art, the quality control of the array probes was conducted very stringently, and the analyses are sound. The paper is very well written. Thus, I have only a few questions.

1) Why were the sex-chromosomal probes were generally excluded from the analyses (lines 189 and 681), which I could not clarify after briefly checking ref [26] (Zhou et al. 2017)?

2) QC and normalization methods lines 678-681: it seems that a sentence is missing in the paragraph. Were the principal components that were significantly (p<0.01) associated with slide, chip, sex, etc. subsequently included in the functional normalization (ref [83])? If so, did the authors check if these principal components were associated with the cohort-specific trait (e.g. ALS, PD, AD) to avoid a potential over-correction in the functional normalization procedure (which recommends using control probe based principal components instead)?

3) For completeness, please state that the DNAm related association test were two-sided (which I assume).

**Reviewer 2**

**Are you able to assess all statistics in the manuscript, including the appropriateness of statistical tests used?** Yes, and I have assessed the statistics in my report.

**Comments to author:**

The authors perform a meta analysis of blood based differential methylation association in AD, PD, ALS, and SCZ. Through a pan-disease meta analysis they identify 12 genome-wide significantly different DNA methylation probes. In addition they perform extensive sensitivity analyses including cross validation and inferring immune cell type proportions to assess the robustness of their findings, as well as determining if their scores are predictive of relevant inflammatory phenotypes. Their finding of FKBP5 association is encouraging, as that has strong independent evidence of being related to neurodegeneration and aging in other studies. Overall the methods appear quite rigorous, and the analyses well thought out. That being said, there are some weak points that could be addressed to strengthen the manuscript and improve the claim that these differentially methylated sites are truly disease relevant.

Major Concerns

1. A meta-analysis across AD, PD, and ALS is an interesting way to boost power to detect potential shared disease etiology across multiple neurodegenerative diseases, especially in a peripheral tissue modality that historically has had poor signal for identification of disease relevant biomarkers. That being said I think more work could be done to strengthen the case that these loci are meaningful to the etiology of AD, PD, and/or ALS. As far as I can tell, they looked for DNA methylation probes that had corresponding methylation QTL, then looked to see if those that had mQTL overlapped with GWAS association. I am curious as to why they did not consider looking at the GWAS statistics from AD, PD, and ALS for the 12 loci they identified as having differentially expressed DNA methylation in a more comprehensive analysis without requiring the loci to also have an mQTL? There could be reasons why an mQTL would not be detected (e.g. power, appropriate defined disease relevant population cohort) that could hide potential causal genetic relationships to disease with marginal signal in GWAS that may be showing up in the DNA methylation data as well. Also - it would be nice to see a comparison to the Kunkle et al. (2019) IGAP AD GWAS summary statistics, as the Marioni et al. paper uses a much looser definition of AD case status (based on familial status as opposed to neuropathological confirmed case status).

2. The gene set enrichment analysis is underwhelming, and seems to be primarily descriptive, and potentially redundant with the observation that immune cell proportions in blood are confounded with disease status (e.g. TGFb signaling activates SMADs enrichment may be due to overrepresentation of a particular immune cell population). Instead of these type of analyses, I would like to see in addition to the aforementioned expanded comparison to GWAS signal, that the authors look at some of the eQTL (e.g. https://www.biorxiv.org/content/10.1101/638544v1), xQTL (https://www.ncbi.nlm.nih.gov/pmc/articles/PMC5785926/ ), or pQTL work coming out of the AMP-AD consortium to see if the signals they are identifying are showing up in relevant brain tissue in a panQTL fashion

3. Finally, I could find mention of the prior work showing the relevance of DNA methylation in AD/PD/ALS in the intro, but I could not find a formal comparison of their findings with the findings from these previous studies. I would be curious if there is any overlap, and if not they should state so.

Minor Concerns

1. The authors use a lot of abbreviations. I would recommend removing some of the non obvious abbreviations for ease of reading.

2. The text in all the figures is quite small. I would strongly recommend dramatically increasing the text size, especially figure 3. Also the resolution of Figure 3 is quite poor when zooming in, I can't read any of the text.

**Authors Response**

**Point-by-point responses to the reviewers’ comments:**

**Response to reviewers**

**Referee #1:**

[Reviewer] “The authors performed an epigenome-wide association using Illumina DNA methylation BeadChips and blood samples to test for CpG sites which DNA methylation level is associated with neurodegenerative disorders. A subsequent meta-analysis of the ALS, PD and AD results revealed 12 significantly associated CpG sites. In subsequent analyses, it was tested whether these results were driven by inflammatory markers including blood cell type composition. Further characterization of the results included a gene-set enrichment analysis. The results are very interesting and well described. The applied methods are state-of-the-art, the quality control of the array probes was conducted very stringently, and the analyses are sound. The paper is very well written. Thus, I have only a few questions.”

[Author Response] We thank the reviewer for the feedback on our manuscript. This is a very concise and precise description of our findings.

1) [Reviewer] Why were the sex-chromosomal probes were generally excluded from the analyses (lines 189 and 681), which I could not clarify after briefly checking ref [26] (Zhou et al. 2017)?

[Author Response] This is a great question raised by the reviewer. Sex chromosomes have often been excluded from GWAS analyses for convenience, which we agree is not a justifiable reason. In contrast, excluding sex-chromosome probes in DNA methylation studies is justified by biological and statistical reasons. Because there are two copies of X in women and one in men, we know that in women one of the two X-chromosomes, at random, is inactivated. In principle, male and female X chromosome data can be analysed separately and meta-analysed, but necessarily the power is much reduced. Although limited by sample size in the current study we agree it would be ideal to include sex-linked probes in further analyses with larger sample sizes, as we may be missing important biological mechanisms. The DNA methylation data we have released into dbGAP and GEO includes the X-chromosome data to facilitate future analyses on larger sample sizes.

2) [Reviewer] QC and normalization methods lines 678-681: it seems that a sentence is missing in the paragraph. Were the principal components that were significantly (p<0.01) associated with slide, chip, sex, etc. subsequently included in the functional normalization (ref [83])? If so, did the authors check if these principal components were associated with the cohort-specific trait (e.g. ALS, PD, AD) to avoid a potential over-correction in the functional normalization procedure (which recommends using control probe based principal components instead)?

[Author Response] We thank the reviewer for spotting this and agree the description may induce additional confounding to the reader. We have now changed the main text to (lines 696-701): “Functional normalization was performed to remove technical variation, as described elsewhere [90]. Briefly, probe intensity quantiles were normalized between samples by fitting linear models with these quantiles to the top principal components of the control probe matrix. After normalization, the most variable probes (m = 20,000) were extracted, decomposed into principal components and each component regressed against slide, chip column, chip row and sex to test for batch effects. The association detection p-value threshold was set to 0.01.”

3) [Reviewer] For completeness, please state that the DNAm related association test were two-sided (which I assume).

[Author Response] We thank the reviewer for the suggestion. For completeness, we have now added “The null hypothesis (H_0_: *b_i_* = 0) can then be tested by a two-sided t test given $\hat{b}_{i}$ and its s.e.” in lines 756-757, of the Methods section.

**Reviewer #2:**

Major Concerns

1. [Reviewer] A meta-analysis across AD, PD, and ALS is an interesting way to boost power to detect potential shared disease etiology across multiple neurodegenerative diseases, especially in a peripheral tissue modality that historically has had poor signal for identification of disease relevant biomarkers. That being said I think more work could be done to strengthen the case that these loci are meaningful to the etiology of AD, PD, and/or ALS. As far as I can tell, they looked for DNA methylation probes that had corresponding methylation QTL, then looked to see if those that had mQTL overlapped with GWAS association. I am curious as to why they did not consider looking at the GWAS statistics from AD, PD, and ALS for the 12 loci they identified as having differentially expressed DNA methylation in a more comprehensive analysis without requiring the loci to also have an mQTL? There could be reasons why an mQTL would not be detected (e.g. power, appropriate defined disease relevant

population cohort) that could hide potential causal genetic relationships to disease with marginal signal in GWAS that may be showing up in the DNA methylation data as well. Also - it would be nice to see a comparison to the Kunkle et al. (2019) IGAP AD GWAS summary statistics, as the Marioni et al. paper uses a much looser definition of AD case status (based on familial status as opposed to neuropathological confirmed case status).

[Author Response] We thank the reviewer for the comments and we agree with the reasons presented for why an mQTL would not be detected, which we also acknowledge in the revised manuscript in lines 260-262, i.e., “We found no evidence for mQTL overlap with GWAS hits (Supplementary Table 4), which could reflect lack of power in both MWAS and GWAS, as has previously been observed for body-mass index (BMI) [40].” We have thus investigated whether the loci +- 500kb of each of the 12 DMPs overlapped with GWAS signals from: 1) Marioni et al. 2018 [1], 2) Nalls et al. 2019 [2] , 3) Nicolas et al. [3] , 4) meta-analysis of 1, 2 and 3, and 5) Kunkle et al. 2019 [4] (Supplementary Figures 9-13, respectively). As for the mQTL analysis, we found no evidence for overlap with GWAS signals (i.e., with p < 5x10^-8^) in the pre-defined loci. The strongest signal overlapping with the pre-defined loci was from our meta-analysis of AD, ALS and PD, albeit non-significant: SNP rs112184630 (p_META_GWAS_ = 5.91x10^-7^), located on chromosome 9q, which has been shown to be a blood eQTL for genes TOR1A (p = 6.74x10^-16^), FBP1 (p = 3.04x10^-15^), C9orf78(p = 9.52x10^-12^) [5] , and also PTGES (p = 7.92x10^-6^) [6]. We observed no overlap with either the Kunkle et al. GWAS summary statistics nor with Marioni et al. GWAS summary statistics. As requested by the IGAP consortium, we added the cohort description in the methods section (lines 804-819) and updated the acknowledgements section (lines 1035-1051). The changes are highlighted in yellow.

Supplementary Figure 9 - Overlap of the AD GWAS p-values from Marioni et al. 2018 [1] (black dots) and p-values from differentially methylated positions from the DNA methylation meta-analysis of AD, PD and ALS (red dots). Loci shown are +- 500kb from each CpG.

Supplementary Figure 10 - Overlap of the PD GWAS p-values from Nalls et al. 2019 [2] (black dots) and p-values from differentially methylated positions from the DNA methylation meta-analysis of AD, PD and ALS (red dots). Loci shown are +- 500kb from each CpG.

Supplementary Figure 11 - Overlap of the ALS GWAS p-values from Nicolas et al. 2018 [3] (black dots) and p-values from differentially methylated positions from the DNA methylation meta-analysis of AD, PD and ALS (red dots). Loci shown are +- 500kb from each CpG.

Supplementary Figure 12 - Overlap of GWAS p-values from meta-analysis of Marioni et al. 2019, Nalls et al. 2019 and Nicolas et al. 2018 (black dots) and p-values from differentially methylated positions from the DNA methylation meta-analysis of AD, PD and ALS (red dots). Loci shown are +- 500kb from each CpG.

Supplementary Figure 13 - Overlap of the AD GWAS p-values from Kunkle et al. 2019 [4] (black dots) and p-values from differentially methylated positions from the DNA methylation meta-analysis of AD, PD and ALS (red dots). Loci shown are +- 500kb from each CpG.

2. [Reviewer] The gene set enrichment analysis is underwhelming, and seems to be primarily descriptive, and potentially redundant with the observation that immune cell proportions in blood are confounded with disease status (e.g. TGFb signaling activates SMADs enrichment may be due to overrepresentation of a particular immune cell population). Instead of these type of analyses, I would like to see in addition to the aforementioned expanded comparison to GWAS signal, that the authors look at some of the eQTL (e.g. https://www.biorxiv.org/content/10.1101/638544v1), xQTL (https://www.ncbi.nlm.nih.gov/pmc/articles/PMC5785926/ ), or pQTL work coming out of the AMP-AD consortium to see if the signals they are identifying are showing up in relevant brain tissue in a panQTL fashion

[Author Response] We have looked at the suggested resources and found many xQTLs to overlap with the pre-defined loci (i.e., +- 500kb of each of the 12 DMPs) (**Supplementary Table 5**). We agree that the gene-set enrichment analysis is primarily descriptive and could potentially still be confounded by cell-type proportions differences and have replaced these analyses with a new results section, based on the previous comments (lines 249-278):

“GWAS signals do not overlap with loci centered at the 12 differentially methylated positions

We next investigated if the 12 DMPs overlapped with brain [35] or blood [36] methylation quantitative trait loci (mQTL) regions (p < 5x10^-8^) and SNPs (p < 5x10^-8^) from a sample-size weighted meta-analysis of publicly available GWAS summary statistics for AD (N = 368,440) [37], ALS (N = 80,610) [38] and PD (minimum N = 520, maximum N = 482,730, excluding the 23andMe cohort) [39] (Supplementary Figure 8). Three of the 12 DMPs overlapped with blood mQTLs (number of mQTLS; m = 266), and another four overlapped with brain mQTLs (m = 86) with the top mQTLs presented in Supplementary Table 4. We found no evidence for mQTL overlap with GWAS hits (Supplementary Table 4), which could reflect lack of power in both MWAS and GWAS, as has previously been observed for body-mass index (BMI) [40].

Since lack of power could hide potential causal genetic relationships to disease with marginal signals in GWAS that may be present in the DNA methylation data as well, we further investigated whether the loci located +- 500kb of each of the 12 DMPs overlapped with the previously mentioned GWAS signals from each disorder and our meta-analysis (Supplementary Figures 9-12). For AD, we additionally compared with GWAS results from clinically diagnosed AD cases, from Kunkle et al (N = 63,926) [41] (Supplementary Figure 13). As for the mQTL analysis, we found no evidence for overlap with GWAS signals (i.e., with p < 5x10^-8^) in the pre-defined loci. The strongest overlapping signal was from our meta-analysis of AD, ALS and PD GWAS, albeit non-significant: SNP rs112184630 (p_META_GWAS_ = 5.91x10-7), located in chromosome 9q, which has been shown to be a blood eQTL for genes TOR1A (p = 6.74x10^-16^), FBP1 (p = 3.04x10^-15^), C9orf78(p = 9.52x10^-12^) [42], and also PTGES (p = 7.92x10^-6^) [43].

Finally, we expanded our query within these loci to brain eQTL (N = 1,433), mQTL (N = 411) and haQTL (N = 411) summary statistics from the AMP-AD consortium [44, 45]. Six of the twelve loci showed significant signals across all xQTLs, suggesting these results could be relevant to brain tissue in a panQTL manner (Supplementary Figure 14). The top eQTL per gene

in the queried 1Mb window can be found in Supplementary Table 5.”

Supplementary Figure 14 - Overlap with molecular quantitative trait loci (xQTLs), from AMP-AD consortium [7, 8], at pre-defined loci ±500kb of each of the 12 differentially methylated positions, from the MOMENT meta-analysis of AD, ALS and PD. mQTLs - methylation quantitative trait loci (gray); eQTLs - gene expression quantitative trait loci (orange); haQTLs - histone acetylation quantitative loci (blue). The numbers on top of the bars refer to the number of unique genes within that region, with a significant eQTL.

3. [Reviewer] Finally, I could find mention of the prior work showing the relevance of DNA methylation in AD/PD/ALS in the intro, but I could not find a formal comparison of their findings with the findings from these previous studies. I would be curious if there is any overlap, and if not they should state so.

[Author Response] We thank the reviewer for the comment and appreciate the need to compare with previous results. It is important to note that the work the reviewer is referring to has been done utilizing cohorts we analyzed in our meta-analysis, with the same methodology (this is true for PD [9] and ALS [10]). Thus, any correlation analysis would be biased since the cohorts fully overlap. In the case of AD, we had cited two major studies focused on brain DNA methylation differences between cases and controls. However, since our first submission to Genome Biology, a new study has been published analyzing whole blood DNA methylation data collected from 284 individuals in the AddNeuroMed study (which included 89 nondemented controls, 86 patients with Alzheimer's disease, and 109 individuals with mild cognitive impairment) [11, 12].The methodology, questions asked, and sample sizes were very different; they performed an analysis of variance (ANOVA) to test for DNA methylation differences across all 3 groups. A post-hoc Tukey's honest significant difference test was then applied to the results to compare methylation levels between each of the 3 diagnostic groups at baseline (controls, mild cognitive impairment, and AD). In our analyses we focus on differences between controls and AD only, and we make use of mixed-linear methods, that have been shown to reduce false-positive rate in this type of analyses. Nonetheless, we compared with the previous results identified from the Tukey's HSD test of control and Alzheimer's disease blood samples. Only 482 of the top 1000 DMPs in the previous AD study were present in our meta-analysis. 694 and 809 of the top 1000 DMPs from PD and ALS studies, respectively, were present in our meta-analysis. 5 and 6 DMPs from our meta-analysis overlapped with the top 1000 DMPs from the PD and ALS studies, respectively. No DMP from our meta-analysis overlapped with the top 1000 DMPs from the AD study (**Supplementary Figure 23**). For clarification, we have added the following to the discussion section (lines 406-410):

“We did not find any overlap with the top signals from a previous AD blood MWAS [15]. 5 and 6 DMPs from our meta-analysis overlapped with the top signals from a blood MWAS of PD [18], and ALS [20], respectively (Supplementary Figure 23), but it is important to note the PD and ALS cohorts from these studies were included in our meta-analysis.”

Supplementary Figure 23 - No evidence for overlap of results from our MOMENT meta-analysis of blood DNA methylation data from AD, ALS and PD with previous results from blood MWAS of the individual phenotypes. **A)** -log10(p-values) of MOMENT meta-analysis of AD, PD and ALS (y-axis) vs -log10(p-values) from the Tukey's HSD test of control and Alzheimer's disease blood samples top 1000 DMPs (x-axis) [12], **B)** -log10(p-values) of MOMENT meta-analysis of AD, PD and ALS (y-axis) vs -log10(p-values) from top 1000 DMPs of MOMENT MWAS of ALS (x-axis) [10] and **C)** -log10(p-values) of MOMENT meta-analysis of AD, PD and ALS (y-axis) vs -log10(p-values) from top 1000 DMPs of MOMENT meta-analysis of PD (x-axis) [9]. Red dotted line indicates the Bonferroni corrected p-value from the MOMENT meta-analysis of AD, ALS and PD. Only 482 of the top 1000 DMPs in the previous AD study were present in our meta-analysis. 694 and 809 of the top 1000 DMPs from PD and ALS studies, respectively, were present in our meta-analysis. 5 and 6 DMPs from our meta-analysis overlapped with the top 1000 DMPs from the PD and ALS studies, respectively. No DMP from our meta-analysis overlapped with the top 1000 DMPs from the AD study.

Minor Concerns

1. [Reviewer] The authors use a lot of abbreviations. I would recommend removing some of the non obvious abbreviations for ease of reading.

[Author Response] We thank the reviewer for noticing this. We have replaced the following abbreviations for ease of reading: NDs, with neurodegenerative disorders; SCZ - schizophrenia, RA - rheumatoid arthritis; DNAm - DNA methylation.

2. [Reviewer] The text in all the figures is quite small. I would strongly recommend dramatically increasing the text size, especially figure 3. Also the resolution of Figure 3 is quite poor when zooming in, I can't read any of the text.

[Author Response] We thank the reviewer for pointing this out. We agree the text size in Figure 3 was too small and have now increased the font size. We have confirmed the resolution of the raw images to be of good quality when zooming in, so there may have been an issue when converting to pdf.

## **References**

1.	Marioni RE, Harris SE, Zhang Q, McRae AF, Hagenaars SP, Hill WD, Davies G, Ritchie CW, Gale CR, Starr JM, et al: **GWAS on family history of Alzheimer’s disease.** *Translational Psychiatry* 2018, **8:**99.

2.	Nalls MA, Blauwendraat C, Vallerga CL, Heilbron K, Bandres-Ciga S, Chang D, Tan M, Kia DA, Noyce AJ, Xue A, et al: **Identification of novel risk loci, causal insights, and heritable risk for Parkinson's disease: a meta-analysis of genome-wide association studies.** *The Lancet Neurology* 2019, **18:**1091-1102.

3.	Nicolas A, Kenna KP, Renton AE, Ticozzi N, Faghri F, Chia R, Dominov JA, Kenna BJ, Nalls MA, Keagle P, et al: **Genome-wide Analyses Identify KIF5A as a Novel ALS Gene.** *Neuron* 2018, **97:**1268-1283.e1266.

4.	Kunkle BW, Grenier-Boley B, Sims R, Bis JC, Damotte V, Naj AC, Boland A, Vronskaya M, van der Lee SJ, Amlie-Wolf A, et al: **Genetic meta-analysis of diagnosed Alzheimer’s disease identifies new risk loci and implicates Aβ, tau, immunity and lipid processing.** *Nature Genetics* 2019, **51:**414-430.

5.	Jansen R, Hottenga J-J, Nivard MG, Abdellaoui A, Laport B, de Geus EJ, Wright FA, Penninx BWJH, Boomsma DI: **Conditional eQTL analysis reveals allelic heterogeneity of gene expression.** *Human molecular genetics* 2017, **26:**1444-1451.

6.	Bonder MJ, Luijk R, Zhernakova DV, Moed M, Deelen P, Vermaat M, van Iterson M, van Dijk F, van Galen M, Bot J, et al: **Disease variants alter transcription factor levels and methylation of their binding sites.** *Nature Genetics* 2017, **49:**131-138.

7.	Ng B, White CC, Klein H-U, Sieberts SK, McCabe C, Patrick E, Xu J, Yu L, Gaiteri C, Bennett DA, et al: **An xQTL map integrates the genetic architecture of the human brain's transcriptome and epigenome.** *Nature neuroscience* 2017, **20:**1418-1426.

8.	Sieberts SK, Perumal T, Carrasquillo MM, Allen M, Reddy JS, Hoffman GE, Dang KK, Calley J, Ebert PJ, Eddy J, et al: **Large eQTL meta-analysis reveals differing patterns between cerebral cortical and cerebellar brain regions.** *bioRxiv* 2019**:**638544.

9.	Vallerga CL, Zhang F, Fowdar J, McRae AF, Qi T, Nabais MF, Zhang Q, Kassam I, Henders AK, Wallace L, et al: **Analysis of DNA methylation associates the cystine–glutamate antiporter SLC7A11 with risk of Parkinson’s disease.** *Nature Communications* 2020, **11:**1238.

10.	Nabais MF, Lin T, Benyamin B, Williams KL, Garton FC, Vinkhuyzen AAE, Zhang F, Vallerga CL, Restuadi R, Freydenzon A, et al: **Significant out-of-sample classification from methylation profile scoring for amyotrophic lateral sclerosis.** *npj Genomic Medicine* 2020, **5:**10.

11.	Lovestone S, Francis P, Kloszewska I, Mecocci P, Simmons A, Soininen H, Spenger C, Tsolaki M, Vellas B, Wahlund L-O, et al: **AddNeuroMed—The European Collaboration for the Discovery of Novel Biomarkers for Alzheimer's Disease.** *Annals of the New York Academy of Sciences* 2009, **1180:**36-46.

12.	Roubroeks JAY, Smith AR, Smith RG, Pishva E, Ibrahim Z, Sattlecker M, Hannon EJ, Kłoszewska I, Mecocci P, Soininen H, et al: **An epigenome-wide association study of Alzheimer’s disease blood highlights robust DNA hypermethylation in the HOXB6 gene.** *Neurobiology of Aging* 2020.

**Second round of review**

**Reviewer 1**

I thank the authors for satisfactorily clarifying my points raised. I have no further questions or concerns.
